# Profile and resistance levels of 136 integron resistance genes

**DOI:** 10.1038/s44259-023-00014-3

**Published:** 2023-10-11

**Authors:** Alberto Hipólito, Lucía García-Pastor, Ester Vergara, Thomas Jové, José Antonio Escudero

**Affiliations:** 1 Molecular Basis of Adaptation. Departamento de Sanidad Animal. Facultad de Veterinaria de la Universidad Complutense de Madrid, Madrid, Spain; 2 VISAVET Health Surveillance Centre, Universidad Complutense de Madrid, Madrid, Spain; 3 INSERM, CHU Limoges, RESINFIT, University of Limoges, Limoges, France

**Keywords:** Antimicrobial resistance, Bacterial genetics

## Abstract

Integrons have played a major role in the rise and spread of multidrug resistance in Gram-negative pathogens and are nowadays commonplace among clinical isolates. These platforms capture, stockpile, and modulate the expression of more than 170 antimicrobial resistance cassettes (ARCs) against most clinically-relevant antibiotics. Despite their importance, our knowledge on their profile and resistance levels is patchy, because data is scattered in the literature, often reported in different genetic backgrounds and sometimes extrapolated from sequence similarity alone. Here we have generated a collection of 136 ARCs against 8 antibiotic families and disinfectants. Cassettes are cloned in a vector designed to mimic the genetic environment of a class 1 integron, and transformed in *Escherichia coli*. We have measured the minimal inhibitory concentration (MIC) to the most relevant molecules from each antibiotic family. With more than 500 MIC values, we provide an exhaustive and comparable quantitation of resistance conferred by ARCs. Our data confirm known resistance trends and profiles while revealing important differences among closely related genes. We have also detected genes that do not confer the expected resistance, to the point of challenging the role of the whole family of *qac* genes in resistance against disinfectants. Our work provides a detailed characterization of integron resistance genes at-a-glance.

## Introduction

The emergence of multidrug resistant bacteria is widely regarded as one of the major global health concerns of the 21^st^ century. In 2014, it was estimated that AR would become the main cause of death in the world by 2050, with ten million casualties per year^1^. Only five years later, new estimations have revealed that we are already somewhere between 1.5 and 5 million deaths every year, suggesting that previous predictions might be reached earlier^2^. Evolution towards resistance is fostered by the dissemination of resistance determinants through horizontal gene transfer (HGT). Multidrug resistance was first observed among *Shigella* spp. isolates in Japan during the 1950’s^3^. These isolates contained a conjugative plasmid with a novel genetic element capable of stockpiling resistance genes: the integron^4^. Integrons are platforms that capture genes encoded in integron cassettes (ICs), stockpiling them in an array, and modulating their expression (for a review see refs. ^5,6^). Five classes of integron have been mobilized from the chromosomes of environmental bacteria onto conjugative plasmids, reaching our hospitals^7^. The class 1 integron is by far the most prevalent and clinically relevant^8^. It is estimated that humans and animals shed to the environment 10^23^ copies of class 1 integrons per day^9^. This way, integrons connect the genomes of clinically relevant human and animal pathogens with those of environmental bacteria, in a paradigmatic example of the One Health concept^10,11^. Indeed, since their initial appearance in *Shigella* containing two resistance genes (*aadA1* and *sul1*), integrons have brought to the clinical setting 175 other resistance genes against most clinically relevant antibiotics, proving their key role in this crisis^12,13^.

Integron cassettes generally contain promoterless genes that are expressed from a dedicated promoter (Pc) in the platform. Although some exceptions to this rule are known -such as cassettes containing promoters^14–18^ - it is generally accepted that this leads to a gradient of expression that fades with distance to the promoter, making the last cassettes of the array less expressed. Under antibiotic treatment, the integrase is expressed, allowing the reshuffling of cassettes within the array^5,19–21^, and inserting them randomly in first position, where their expression is maximal. Comparing profiles and resistance levels of integron resistance cassettes is not straightforward, since they can be found in different bacterial species, plasmid backbones, classes of integrons, and under the control of Pc promoter variants of different strengths. Furthermore, some cassettes are annotated by sequence homology, and functions are not experimentally proven. Hence, obtaining a detailed view of the function of these genes from independent reports scattered in the literature is difficult.

Here, we provide a comparative and comprehensive study of ARCs resistance profiles. We have generated a collection of 136 strains bearing specific ARCs in pMBA, a vector designed to mimic their native genetic environment -a class 1 integron. We introduced this collection in *Escherichia coli* and quantitated the resistance conferred by each ARC by measuring the minimal inhibitory concentration (MIC) of each strain in the collection against several antimicrobials. The data presented here provides a detailed guide of ARCs resistance profiles at-a-glance. Incidentally, our collection serves as a repository of resistance markers for biotechnological purposes.

## Results

### Generation of the pMBA collection

**Fig. 1:**
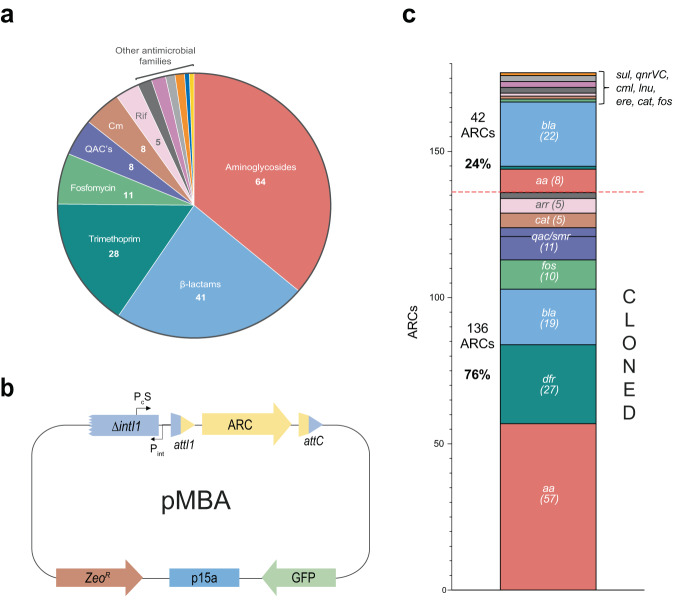
Antimicrobial resistance cassettes in integrons and the generation of the pMBA collection. **a** Distribution of ARCs families found in mobile integrons retrieved from IntegrAll database analysis (QACs quaternary ammonium compounds, Cm chloramphenicol, Rif Rifampin) (Modified from ref. ^13^). **b** Diagram of pMBA-derived vectors encoding ARCs. **c** Histogram showing the number of ARCs cloned or not against each antimicrobial family (*aa* aminoglycoside resistant gene, *dfr* dihydrofolate reductase, *bla* beta-lactamase, *fos* phosphomycin resistance gene, *qac* quaternary ammonium compounds resistance gene, *smr* small multidrug resistance gene, *cat* and *cml* chloramphenicol resistance genes, *arr* rifampicin resistance gene, *sul* sulfonamide resistance gene, *qnrVC* quinolone resistance gene, *lnu* lincosamide resistance gene, *ere* erythromycin resistance gene).

In a previous work we retrieved all ARCs from the IntegrAll database, a curated repository containing all integron cassettes found in mobile integrons. Applying a 95% sequence identity cutoff^13^, we identified 177 different integron cassettes (Fig. 1a) that we have now synthesized and cloned in pMBA, a vector that mimics the natural environment of a class 1 integron (Fig. 1b). pMBA is based on a p15a origin of replication and has a zeocin resistance marker that provides universal selection, independent of the ARC it contains. pMBA provides the genetic environment of a cassette in first position of a class 1 integron. All ARCs are cloned in the integration site (*attI1*) mimicking an integrase-mediated integration reaction. Cassettes are located in close proximity to a strong Pc promoter^22^ (PcS) encoded within the integrase gene. It is of note that the *intI1* gene in pMBA is truncated to avoid the costly and deleterious activity of the integrase. Downstream the integron there is a GFP gene that provides a second verification of plasmid presence.

All ARCs were transformed in *Escherichia coli* MG1655, and the sequence of the ARC was verified. The choice of this bacterium is supported by its key importance as opportunistic pathogen in community and hospital settings and its tendency towards antibiotic resistance (it is a member of the ESKAPEE group). Among the 177 ARCs, we were able to establish 136 pMBA with the correct sequence (Fig. 1c). The remaining 41 ARCs could, in most cases, be cloned but with mutations, and were not included in this study. The 136 ARCs cloned represent 83% of ARCs ever discovered in *E. coli*, and 76% of all ARCs described to date in class 1 integrons. To the best of our knowledge, this is the largest collection of integron ARCs to date. Notably, they are located within in their native genetic environment, making of this collection a unique tool to characterize antimicrobial resistance mediated by integrons.

### Antimicrobial resistance characterization

After establishing our library of ARCs in pMBA we sought to determine the resistance levels and profiles for all genes. In the cases of antibiotic families with structurally different molecules and generations -mainly beta-lactams and aminoglycosides- a preliminary profile was obtained using disc diffusion experiments (data is available in supplementary materials). We then measured the minimum inhibitory concentration for a subset of highly relevant molecules. Along the next lines, we provide a brief overview of the importance and mechanism of action of each antibiotic family together with resistance mechanisms. We then provide the MIC results for each ARC and antibiotic. Graphs show both the raw levels of resistance (in µg per mL), and the relative fold change compared to the empty vector control. Bars represent the mean of the three assays (dots). If available, the EUCAST clinical breakpoint is shown as a red dotted line. Gene order in the graph is the result of a hierarchical clustering tree of the proteins encoded in ARCs revealing important phenotypic differences in closely related genes.

#### Aminoglycosides resistance cassettes

Aminoglycosides are included in the WHO’s list of critically important antimicrobials for their efficacy in a wide range of bacterial infections, particularly those caused by Gram-negative bacteria^23^. They alter protein synthesis by tightly attaching to the A-site on the 16 S ribosomal RNA of the 30 S ribosome subunit^24^. Resistance to these antibiotics is commonly conferred by numerous antibiotic modifying enzymes (AMEs) that transfer acetyl-, phosphoryl-, or nucleotidyl/adenyl groups to the antibiotic molecule, lowering the affinity for its target (reviewed in^12,25,26^). Depending on their substrate specificity, these enzymes have different resistance profiles against the broad variety of molecules in the family. Another group of resistance enzymes are 16S-RNA methylases, that modify the target instead of the antibiotic. These enzymes confer extremely high levels of resistance against most aminoglycosides^27^.

Aminoglycoside resistance is the largest group of ARCs in our collection, with 54 different genes (Supplementary Table 1). Integrons encode numerous AMEs including acetyltransferases (*aacA* (31 genes), *aacC* (8) and *sat* (1)), nucleotidyl/adenyltransferases (*aadA* (14) and *aadB* (1)), and phosphotransferases (*aph* (2)), but do not encode, to date, RNA methylases. In this work, we use the nomenclature in Partridge et al.^12^ and the IntegrAll database, but another nomenclature is also used in the field (reviewed in ref. ^26^). We provide the equivalence between nomenclatures when possible, and we include the exact sequence used here for disambiguation purposes (Supplementary Table 2).

**Fig. 2:**
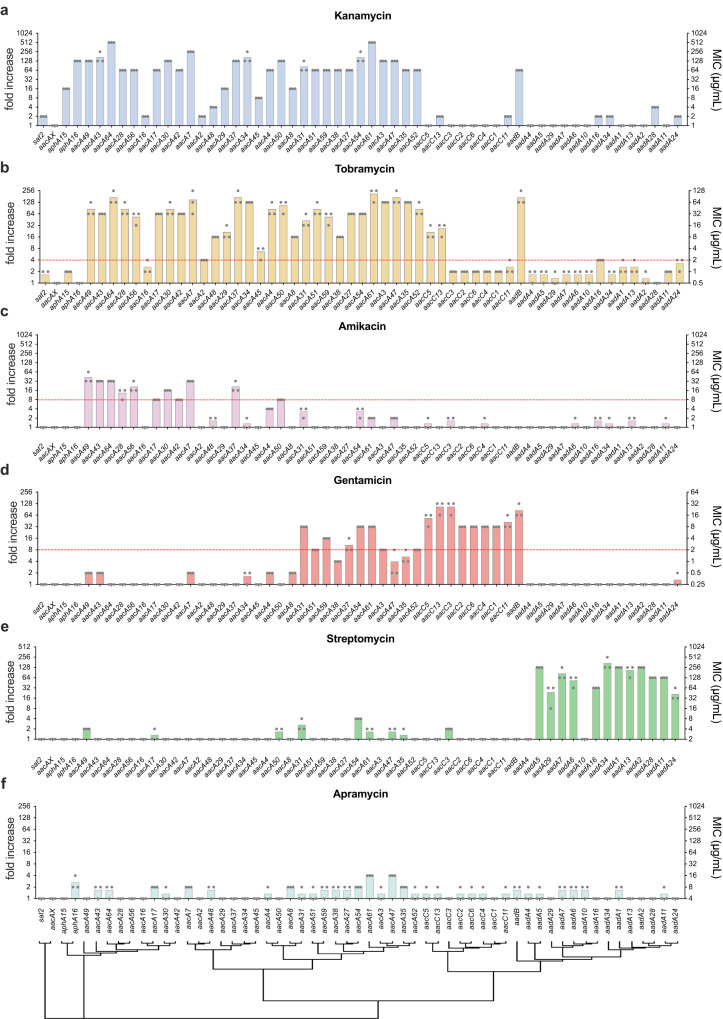
MICs of aminoglycoside resistance cassettes. Antimicrobial resistance to kanamycin (**a**), tobramycin (**b**), amikacin (**c**), gentamicin (**d**), streptomycin (**e**), and apramycin (**f**) is shown as MIC (μg/mL) in the right axis, and resistance fold increase compared to pMBA in the left axis. The MIC is the mean of three biological replicates (black dots) for each strain. A red dotted line represents the clinical breakpoint (EUCAST) for *E. coli* against this antimicrobial. A hierarchical clustering tree showing protein sequence similarity is shown under the graphs.

Given the large number of genes and molecules in this family, we first measured resistance by disc diffusion against seven structurally different aminoglycosides. These included 4,6-disubstituted deoxystreptamines (4,6-DDs) like kanamycin, gentamicin, tobramycin, and amikacin; the 4,5-DD neomycin; the 4-monosubstituted deoxystreptamine apramycin -of use in Veterinary medicine-; and the non-deoxystreptamine streptomycin (Supplementary Fig. 1). We then determined the MIC of kanamycin, tobramycin, amikacin, gentamicin, streptomycin, and apramycin for all ARCs (Fig. 2).

As a general overview, we can see the known trends in resistance where *aacAs* confer resistance against kanamycin and tobramycin; *aacCs* against gentamicin; *aadAs* against streptomycin; *aadB* against gentamicin; *aph* genes confer resistance to kanamycin and *sat2* did not confer resistance against any of the antibiotics tested (Fig. 2). In general, phenotypic differences seem to correlate with protein families. Yet, superposing a dendrogram of sequence relationships reveals known incoherencies in the branching of genes. This highlights that striking phenotypic differences can be found among closely related genes, justifying the need for a study like this.

Most *aacA* genes confer increased resistance against kanamycin (Fig. 2a) and tobramycin (Fig. 2b) often with >64-fold increases. Surprisingly, *aacA2*, *aacA16*, and *aacA45* provide very low resistance (2- to 8-fold increases) despite being closely related to genes with high resistance levels. In fact, *aacA16* does not clearly reach the clinical breakpoint to tobramycin in our setup. Among the *aacA*s, amikacin- and gentamicin- resistance is clearly ARC-dependent (Fig. 2c, d); and *aacAX* does not confer resistance to any antibiotic. On their side, *aacCs* confer clinically relevant resistance to gentamicin (MIC ≥ 2 μg/mL), with up to 128-fold increase; (Fig. 2d). Notably, *aacC5* and *aacC13*, that branch together, also confer clinical resistance against tobramycin (Fig. 2b).

Among the adenyltransferases, *aadA*s present great specificity against streptomycin, (Fig. 2e) conferring 16- to 128-fold increases in resistance. Interestingly, *aadA4* and *aadA10* do not confer increased resistance to streptomycin -or any other antibiotic. It is possible that this is specific of the alleles chosen here, and that other alleles of these genes do confer resistance. The fact that certain alleles might not represent a threat highlights the need for functional and detailed studies like this one. *aadB* shows a profile more related to *aacA*s, not showing activity against streptomycin but instead conferring resistance to kanamycin, tobramycin, and gentamicin (Fig. 2a, b, d).

Within the pMBA collection, *aphA15* and *aphA16* are the only phosphotransferase encoding cassettes (*aphs*). Both genes confer clinical resistance against kanamycin (Fig. 2a), but at different levels: *aphA16* provides 8-fold the resistance of *aphA15* (128-fold vs. 16-fold increases respectively).

*sat2* is described as an acetyl-transferase conferring resistance against streptothricin^28^. This compound has very little clinical relevance due to its toxicity^29^, and has not been tested here. Our data do not corroborate this phenotype, but at least rule out its role in resistance against other more recent or clinically relevant aminoglycosides (Fig. 2). Regarding apramycin resistance (Fig. 2f), an antibiotic of use in Veterinary medicine, it is known that *aac(3)-IV* is the only resistance gene, and it is not found in integrons. Our results generally corroborate this, yet the 4-fold increase in resistance provided by *aacA47* and *aacA61* is a call for caution.

#### Beta-lactam resistance cassettes

Beta-lactam antibiotics are likely the class of antimicrobials with the highest use to treat infectious diseases^30^, with some of its members classified as last resource antibiotics by the WHO. These molecules affect cell wall synthesis by binding to specific proteins called penicillin-binding proteins (PBPs). PBPs are transpeptidases involved in the crosslinking of the peptidoglycan^31^. Beta-lactams can be classified into penicillins, cephalosporins, carbapenems, and monocyclic beta-lactams^30^. These antibiotics are used to treat a plethora of infections in many clinical situations.

**Fig. 3:**
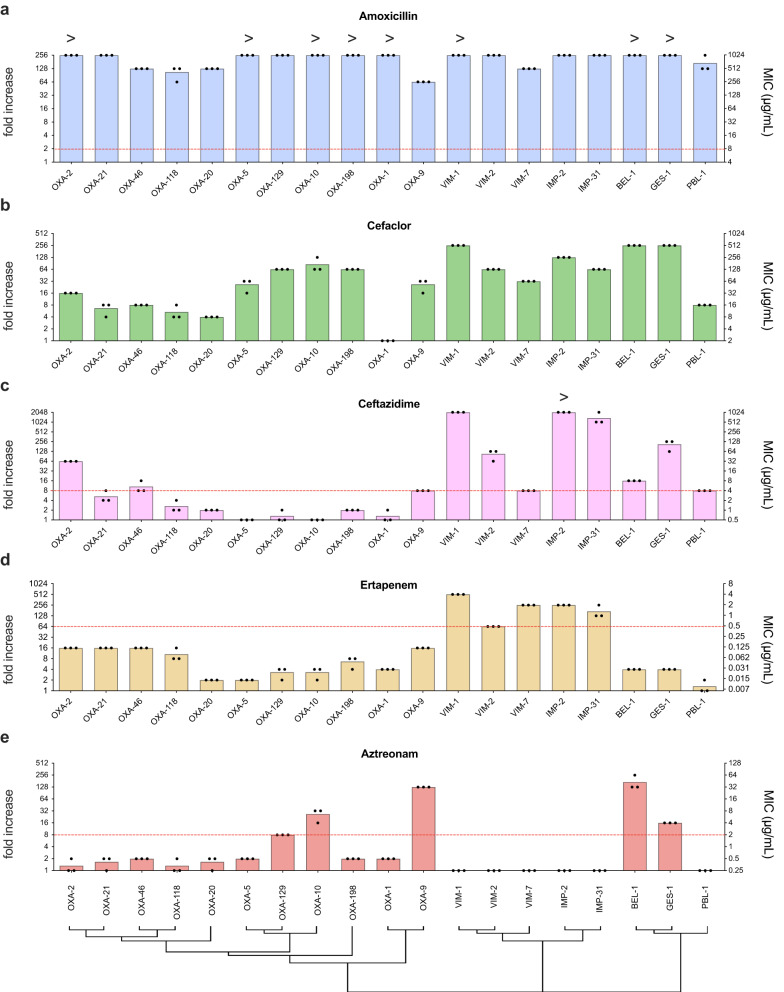
MICs of beta-lactam resistance cassettes. Antimicrobial resistance to amoxicillin (**a**), cefaclor (**b**), ceftazidime (**c**), ertapenem (**d**), and aztreonam (**e**) is shown as MIC (μg/mL) in the right axis, and resistance fold increase compared to the empty pMBA control in the left axis. The MIC is the mean of three biological replicates (black dots) for each strain. A red dotted line represents the clinical breakpoint (EUCAST) for *E. coli* against this antimicrobial. A hierarchical clustering tree showing protein sequence similarity is shown under the graphs.

In our study, we successfully cloned and characterized a total of 19 different gene cassettes, encoding most beta-lactamase classes (Supplementary Table 1). Attending to Ambler´s classification^32^, these included 11 class D enzymes, commonly referred to as oxacillinases (*bla*_OXA_); 5 class B enzymes known as metallo-ß-lactamases (*bla*_VIM_ and *bla*_IMP_ variants) and 3 cassettes encoding class A enzymes, specifically *bla*_GES_, *bla*_BEL_, and *bla*_PBL_. Applying Bush–Medeiros–Jacoby^33^ classification, we can find 14 enzymes enclosed in group II and 5 group III beta-lactamases in pMBA collection. Given the variety of antibiotics and generations within the family, we performed preliminary diffusion antibiograms to select representative antibiotics from different classes (Supplementary Table 3 and Supplementary Fig. 2). We chose a member of the most clinically-relevant beta-lactam classes for MIC determination, namely amoxicillin (an aminopenicillin), cefaclor and ceftazidime (1st and 3rd generation cephalosporins), ertapenem (a carbapenem), and aztreonam (a monobactam) (Fig. 3).

Every ß-lactamase encoding ARC confers high resistance to amoxicillin in a clinically relevant manner (MIC ≥ 8 μg/mL) (Fig. 3) and only *bla*_OXA-1_, *bla*_PBL-1_ and *bla*_BEL-1_ were inhibited by clavulanic acid (Supplementary Fig. 2).

Oxacillinases (*bla*_OXA_ genes) are the most abundant group of ß-lactamases in our collection. This is a broad family of proteins with different resistance profiles, ranging from penicillinases to carbapenemases. Despite not having a clinical breakpoint for cefaclor, fold increases in resistance were high in most cases, except for *bla*_OXA-1_; while only *bla*_OXA-2_ and *bla*_OXA-46_ showed an extended spectrum beta-lactamase (ESBL) profile, conferring resistance against 3^rd^ generation cephalosporins. Importantly, although many oxacillinases in our collection have carbapenemase activity, none reached clinical resistance against ertapenem, and only *bla*_OXA-9_*, bla*_OXA-10_, and *bla*_OXA-129_ conferred resistance against aztreonam.

*bla*_VIM_ genes are well-known carbapenemases found in integrons. Together with *bla*_IMP_ in our collection they confer the expected resistance against cephalosporins and carbapenems, but not monabactams. *bla*_BEL-1_ and *bla*_GES-1_ are ESBLs with similar resistance profiles. They were described for the first time in *Pseudomonas aeruginosa*^34^ and *Klebsiella pneumoniae*^35^ and they both confer resistance to 3^rd^ generation cephalosporins and monobactams, although at different levels. Also, *bla*_BEL-1_ and *bla*_GES-1_ are inhibited by clavulanate. *bla*_PBL1_ is also an ESBL, that reaches clinically relevant resistance to cephalosporins in our conditions, but differs from the previous ones in that it does not confer resistance to monobactams.

#### Antifolate resistance cassettes

**Fig. 4:**
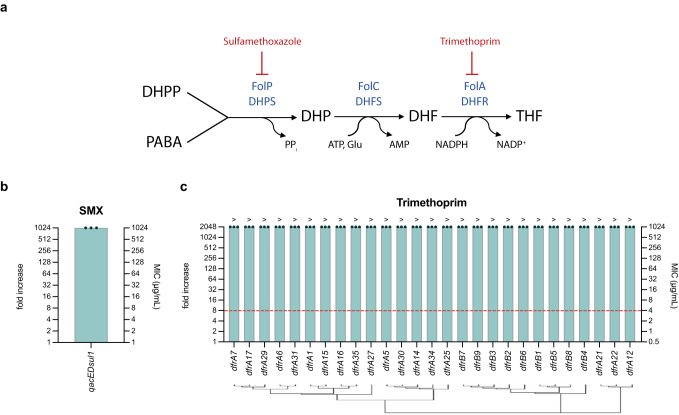
Resistance to antifolates. **a** Folate biosynthesis pathway. FolP/DHPS condenses P-aminobenzoic acid (PABA) and 6-hydroxymethyl-7,8- dihydropterin pyrophosphate (DHPP) into 7,8 dihydropteroate (DHP). FolP is inhibited by sulfonamides (PABA analogs). DHP is then converted to 7,8 dihydrofolate (DHF) by the action of FolC/DHFS; which is again modified by the action of FolA/DHFR into 5,6,7,8-tetrahydrofolate (THF). The action of FolA/DHFR can be inhibited by the drug trimethoprim. Modified from^63^ **b** and **c** MIC of *qacE∆sul1* and *dfr* cassettes against antifolate antibiotics. Antimicrobial resistance to sulfamethoxazole (SMX) (**b**) and trimethoprim (**c**) is shown as MIC (μg/mL) in the right axis, and resistance fold increase compared to the empty pMBA control in the left axis. The MIC is the mean of three biological replicates (black dots) for each strain. A red dotted line represents the clinical breakpoint (EUCAST) for *E. coli* against this antimicrobial. A hierarchical clustering tree showing protein sequence similarity is shown under the graph for trimethoprim.

Antifolates such as sulfonamides and trimethoprim, inhibit the synthesis of tetrahydrofolate at different stages and are commonly used synergistically to treat urinary, respiratory, and gastrointestinal infections. Sulfonamides inhibit the dihydropteroate synthase (DHPS) (FolP), while trimethoprim inhibits the dihydrofolate reductase (DHFR) (FolA) enzymes (Fig. 4a)^36^. Resistance genes encode homologs of these enzymes with reduced affinity to the drugs^37^. They generally confer extremely high resistance levels (beyond solubility of the antibiotic). Only exceptionally, some alleles can provide intermediate levels of resistance if expression is low enough. In integrons, several *dfr* genes conferring resistance to trimethoprim have been reported, while resistance to sulfonamides is conferred by a hybrid cassette in which *qacE* is truncated and fused to *sul1*. This cassette is immobile because it has lost its recombination site, and it is hence often found at the end of arrays, what led to the misconception of it being a conserved 3’ region of class 1 integrons.

Our collection contains 27 *dfrA/B* ARCs and 1 *qacEΔsul1* (Supplementary Table 1). As expected, all genes conferred extreme levels of resistance with MICs of 1024 μg/mL or more (Fig. 4b, c). Incidentally *qacE∆sul1* had to be tested in DH5α because of the high intrinsic resistance levels of MG1655 to sulfamethoxazole.

#### Fosfomycin resistance cassettes

**Fig. 5:**
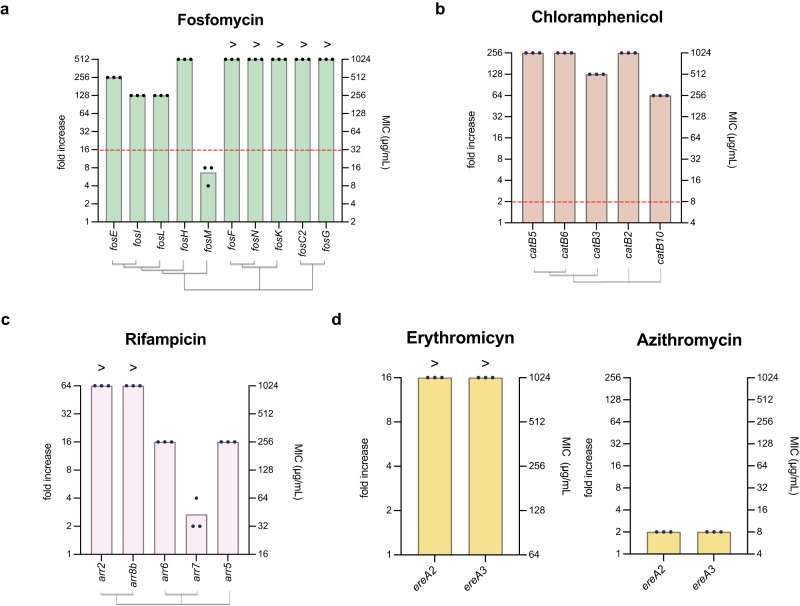
MICs of other ARCs. Antimicrobial resistance to fosfomycin (**a**), chloramphenicol (**b**), rifampicin (**c**), erythromycin, and azithromycin (**d**) is shown as MIC (μg/mL) in the right axis, and resistance fold increase compared to the empty pMBA control in the left axis. The MIC is the mean of three biological replicates (black dots) for each strain. A red dotted line represents the clinical breakpoint (EUCAST) for *E. coli* against this antimicrobial. A hierarchical clustering tree showing protein sequence similarity is shown under the graphs.

Fosfomycin is used to treat urinary tract infections, and skin and soft tissue infections^38^. It inhibits MurA, interfering with peptidoglycan biosynthesis at a step prior to that of beta-lactams. Resistance often arises through mutations in active transporters that prevent the entrance of the antibiotic; the acquisition of plasmid-encoded enzymes that degrade the antibiotic, or the modification of MurA^39^. Integrons encode Fos enzymes that inactivate fosfomycin either through hydrolysation (*fosL* and *fosM* genes), addition of glutathione (*fosC2, fosF, fosG, fosK*) or addition of bacillithiol (*fosE, fosH*, and *fosI*)^40–42^. Here we have determined the MIC to fosfomycin of 10 different *fos* ARCs (Supplementary Table 1) (Fig. 5a). Most alleles confer strong increases in resistance that are clinically relevant (MIC ≥ 32 μg/mL), with the exception of *fosM*, that only increases 8-fold the MIC of the strain, and fails to reach the clinical breakpoint.

#### Chloramphenicol resistance cassettes

Chloramphenicol is a broad-spectrum antibiotic that is now usually reserved for serious infections that have not responded to other antibiotics, due to the risk of serious side effects (for a review see ref. ^43^). This antibiotic inhibits protein synthesis by binding to the 50 S subunit of the bacterial ribosome. Chloramphenicol resistance can be mediated by the modification of the chemical structure of this compound -either by acetylation (CatB)^44^ or phosphorylation-, as well as via efflux pumps such as CmlA^15^. A number of *catB* and *cmlA* ARCs are present in mobile integrons^12^. In the pMBA collection we could only clone *catB* alleles (Supplementary Table 1) that conferred high resistance levels against chloramphenicol, that in all cases reached the clinical breakpoint (MIC ≥ 8) (Fig. 5b).

#### Rifampicin resistance cassettes

Rifampicin acts inhibiting bacterial RNA polymerase (RNAP)^45^. Resistance can be mediated by mutation in the target (the β-subunit of the RNA polymerase encoded in the *rpoB* gene), or by enzymatic modification of the antibiotic; an example of these enzymes are the ADP-ribosylating transferases (encoded in *arr* genes)^46,47^ present in integrons^48^. We have cloned and characterized 8 *arr* variants (Supplementary Table 1 and Fig. 5c). Resistance levels vary significantly across alleles, with two genes providing >64-fold increases in resistance, while *arr7* increases resistance only 2-fold.

#### Erythromycin resistance cassettes

Erythromycin is commonly used to treat skin and respiratory tract infections caused by Gram-positive bacteria. This macrolide inhibits bacterial protein synthesis by binding to the 50 S subunit of the bacterial ribosome. Erythromycin resistance can be due to ribosome methylation (*erm* genes)^49^, erythromycin esterification (*ereA* genes)^16^, and antibiotic transportation (*mef* genes)^50^. Enterobacteria are not generally susceptible to erythromycin, but can be treated in some cases with azithromycin, a broader spectrum macrolide with several therapeutical uses^51^. We have cloned two *ereA* genes in our collection (*ereA2* and *ereA3*) and characterized the resistance against both antibiotics (Fig. 5d). The two alleles increased more than 16-fold the resistance of *E. coli* to erythromycin, reaching MICs >1024 μg/mL. On the other hand, with only two-fold increases in resistance to azithromycin, neither gene is likely to confer clinical resistance to this antibiotic, for which EUCAST provides a loose estimation of the threshold above 16 µg/mL.

#### Quaternary ammonium compounds ARCs (*qac*, *smr*)

**Fig. 6:**
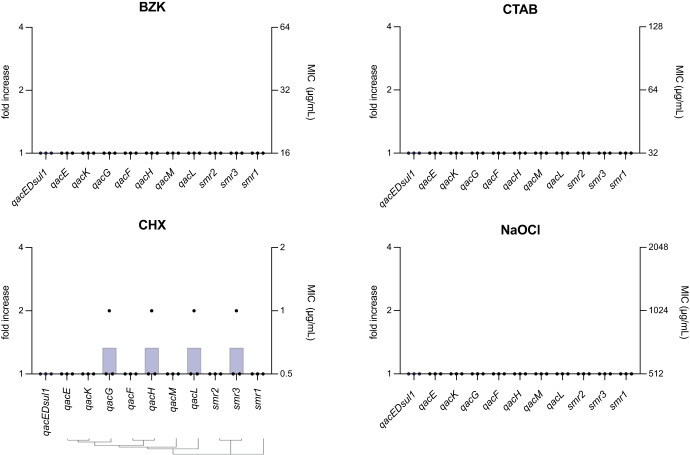
MIC characterization of *qac and smr* ARCs. Antimicrobial resistance to antiseptics chlorhexidine (CHX), benzalkonium chloride (BZK), cetyltrimethylammonium bromide (CTAB), and sodium hypochlorite (NaOCl). MIC (μg/mL) is shown in the right axis, and resistance fold increase compared to pMBA in the left axis. The MIC is the mean of three biological replicates (black dots) for each strain. A hierarchical clustering tree showing protein sequence similarity is shown under the graphs.

Quaternary ammonium compounds (QACs) and antiseptics are commonly used in healthcare settings, food processing facilities, and households to disinfect surfaces and products. QACs antiseptic potential is based on its ability to interact with and disrupt the cell membranes of bacteria, viruses, and fungi^52^. Chlorhexidine (CHX), benzalkonium chloride (BZK), cetyltrimethylammonium bromide (CTAB), and sodium hypochlorite (NaOCl) are examples of commonly used antiseptics. QAC resistance genes (*qacs*) and small multidrug resistance (*smr*) ARCs are generally considered to provide resistance against antiseptics via efflux pumps^53^. Studies characterizing resistance mediated by *qac* and *smr* ARCs show, at best, 2 to 4-fold increases^54,55^. Here we tested the resistance conferred by 8 *qac* and 3 *smr* ARCs (Supplementary Table 1) against CHX, BZK, CTAB, and NaOCl, and found no increase in MIC against any compound (Fig. 6). Only against CHX we found single replicates growing at 2-fold higher concentrations, a variability accepted in the field as not significant, and not changing the final MIC. In our hands, none of these genes confer resistance against disinfectants, a relevant finding with strong implications for co-selection phenomena.

### The genetic context can influence MIC levels in different ways

**Fig. 7:**
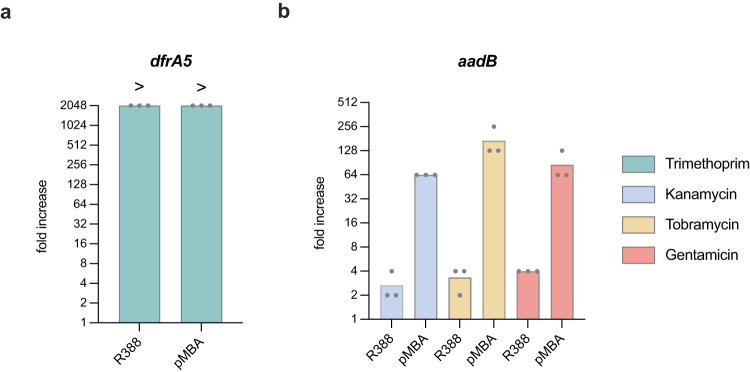
Comparison of the MIC of ARCs in different genetic contexts. Antimicrobial resistance of *dfrA5* (**a**), and *aadB* (**b**), in R388 is shown as resistance fold increase compared to MG1655 without the plasmid. The MIC is the mean of three biological replicates (black dots) for each strain. MIC values for pMBA derivatives are taken from Figs. 2 and 4 and plotted side by side for comparison.

The main aim of this work is to provide a comparative view of ARCs. As mentioned before, cassettes can be found in a variety of genetic backgrounds that influence their expression levels. Therefore it is impossible to provide a single (universal) measure of resistance for ARCs. Consequently, the reader should be aware that the set of MIC values provided here might not be directly translatable to the clinical setting. Indeed, some peculiarities of our setting need to be considered to better interpret these results. Our genetic setup mimics a class 1 integron with a strong variant of the Pc promoter, and is located on a plasmid with approximately 9 copies per cell^56^. This setting was chosen to maximize the phenotype of each gene. Yet integrons are normally borne on large conjugative plasmids with low copy number, and they can encode a variety of Pc promoters with different strengths, or even the combination of two promoters^57^. It is therefore probable that phenotypes in clinical settings are not as strong as the ones reported here, but the impact of genetic context is probably different among resistance mechanisms. Typically, antifolate genes generally have an almost digital behavior with all genes conferring extremely high resistance, while genes encoding modifying enzymes are known to display a more linear behavior with copy number^58^. It is beyond the scope of this work to provide an exhaustive view of the influence of the genetic context for all genes. Yet, to exemplify the influence of genetic context, we have investigated both types of resistance mechanisms in a low number/low expression genetic context. We have determined the MIC of *dfrA5* and *aadB* borne in the low copy plasmid R388^19^ and located in first position of an integron array with a truncated integrase -as they are in pMBA- (Fig. 7) but under the control of a weak Pc promoter (PcW, 26 to 30 times weaker than PcS).

As expected, trimethoprim resistance is not conditioned by the genetic context while aminoglycoside resistance conferred by *aadB* is clearly lower when expressed from a PcW promoter^57^ in a low copy plasmid.

## Discussion

Antimicrobial resistance is a major threat for global health. Integrons are key elements in multidrug resistance among Gram negatives, harboring more than 170 resistance determinants against most antibiotic families. This, together with their high prevalence in clinical isolates^59,60^, make of integrons key genetic elements in the fight against antimicrobial resistance.

The creation of the pMBA collection enables a comprehensive and comparable study of the resistance levels and profiles conferred by resistance genes carried by integrons. It reveals a variety of features of these genes that are not intuitive to most researchers, and for whom this guide can be useful. Also, by coupling MICs to hierarchical clustering trees, one can observe that profiles do not follow strictly the identity signal between proteins, highlighting that genes annotated automatically might not behave as predicted. Indeed, some ARCs do not follow the resistance pattern of the family, like *aadB*, that shows a resistance profile closer to that of acetylases than to other adenylases^19^. In other cases, closely related genes such as *bla*_OXA-1_ and *bla*_OXA-9_ (Fig. 3) show different resistance profiles against cephalosporins and aztreonam, similarly to what we see for *aadA4* and *aadA5* in which the latter confers high streptomycin resistance, while the former (the closest related protein) is sensitive to streptomycin. In more extreme cases, the annotation as a resistance gene seems to be mistaken, as for *aadA10* or *aacAX* (Fig. 2); or at least it is unlikely that these genes are clinically relevant, like for *aacA16* and *fosM*; or even *bla*_OXA-1_ (considering antibiotics other than aminopenicillins) (Fig. 3). The most striking case revealed by our results is that of complete families of genes that do not confer resistance against the compounds described in the literature. Such is the case of *qac* and *smr*, that did not rise the MIC against any of several quaternary ammonium compounds (Fig. 6), although we cannot rule out a stronger impact of these determinants in different conditions, such as in biofilms^55^. All this highlights the need for a detailed study like this one.

We have also shown that the genetic context of ARCs can modulate MIC levels. Because our results are obtained in expression conditions generally higher than those in clinical strains, it follows that genes for which we do not detect resistance can confidently be interpreted as not conferring resistance (at least in this species), while the clinical relevance of others could potentially be lower in other genetic backgrounds.

An interesting aspect of our results is their potential impact in biotechnology. Integron ARCs have been broadly used as resistance markers, and sometimes availability of different markers is a limiting factor when delivering strains with multiple genetic modifications. Our results provide researchers with a useful guide with options that are counterintuitive for many, like the possibility of using erythromycin resistance genes in *E. coli* or the possibility of selecting 5 consecutive markers within a single family of antibiotics: for example, *aacC11* and *aadA5* and can be independently selected with gentamicin and streptomycin. Then, respecting the order, *aphA16* can be selected with kanamycin; *aacA34* with tobramycin; and *aacA49* with amikacin.

**Fig. 8:**
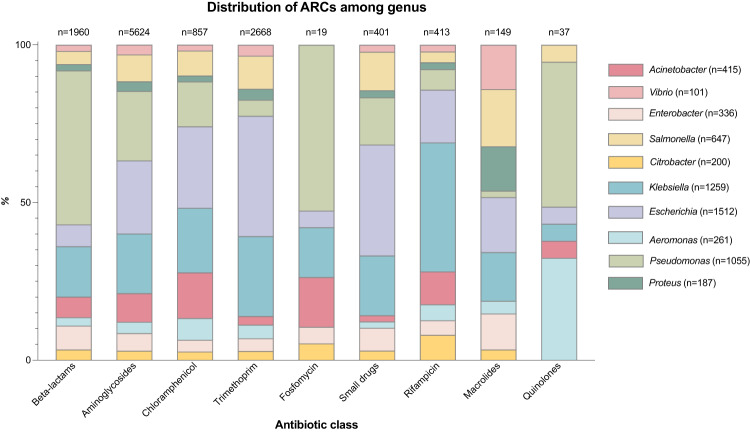
Distribution of ARCs by antibiotic class among different bacterial genus. Each column represents the whole set of reports of ARCs against a given antimicrobial family available in IntegrAll (each ARC can be reported more than once). Colors represent the bacterial genus in which it was found.

A relevant limitation of our work is that we could not clone all ARCs found in mobile integrons (only 136 out of 177). ß-lactamases were the family of ARCs with the lowest cloning success. We suspect there could be biological reasons underlying this. For instance, when looking at the distribution of ARCs among bacterial species, *bla* ARCs represent a low percentage of all ARCs in *E. coli* (4,1%) in comparison with other species as *Pseudomonas spp*. (34,4%). Analyzing the distribution by resistance mechanism shows a similar pattern, with almost 50% of *bla*’s in ARCs being found in *Pseudomonas spp*., while only 7% are found in *E. coli* (Fig. 8). This highlights the possibility that there might be barriers to horizontal gene transfer mediated by integron resistance cassettes. Interestingly, *bla*_VEB-1_ had been successfully cloned in R388 under a PcW^19^ but not in pMBA, suggesting that, in the case of certain genes, high expression levels might be too costly. In this sense, it is also noteworthy that R388 carrying *aadB* in first position confers significantly higher resistance levels to gentamicin in *P. aeruginosa* (64-fold)^19^, compared to *E. coli* (4-fold).

This work provides a detailed guide of the resistance profiles conferred by each ARC, providing a much-needed comparable overview of integron-mediated antibiotic resistance. Our data confirm known relationships between ARC families and antibiotic resistance specificities, but also highlight several exceptions and specific cases of clinical relevance. This specific and individualized information will hopefully help understand the differential prevalence of each ARC in clinics and to a further extent, fighting against antimicrobial resistance.

## Methods

### Bacterial strains, plasmids, and culture conditions

*Escherichia coli* MG1655 and DH5α were used as recipients for all plasmid constructions in this study (Supplementary Table 1). Bacterial strains were cultured at 37 °C in Müller Hinton (MH; Oxoid, UK) liquid medium and MH agar (1.5%) solid medium (BD, France). Zeocin (Invivogen, USA) was added at 100 μg/mL to maintain pMBA plasmid collection in *E. coli*. Liquid cultures were incubated in an Infors Multitron shaker at 200 rpm (Infors HT, Swiss) unless otherwise stated.

### Generation of the pMBA Collection

Plasmids used in this study (Supplementary Table 1) derive from pMBA^13^; a vector that mimics the genetic environment of a class I integron with our cassette of interest in the first position of the array. ARCs retrieved from IntegrAll database as stated in Hipólito et al.^13^, were synthesized in vitro (IDT, USA) adding homology regions with pMBA vector in both 5′ and 3′ ends. To generate pMBA collection we cloned each ARC in the first position of the array (*attI* site) using Gibson Assembly^61^.

Briefly, pMBA vector was linearized and amplified by PCR using Int R bb and GFP F bb primers. Also, each ARC was amplified using gBlock F and gBlock R primers (Supplementary Table 4 and Supplementary Figure 3). Gibson assembly reaction was performed in a final volume of 4 μl containing 1 μl of the linearized vector, 1 μl of the insert (each AR cassette), and 2 μl of the 2X Gibson Assembly Buffer (5X ISOBuffer, 10,000, 147 u/mL T5 exonuclease, 2000 u/mL Phusion polymerase, 40,000 u/mL Taq ligase, dH2O). The mix was incubated for 30 min at 50 °C before being transformed into *E. coli* MG1655/DH5α competent cells. All construction sequences were verified after transformation by Sanger sequencing (Macrogen, South Korea) using the following primers: Int F, GFP R and GFP 2.0 R (Supplementary Table 4 and Supplementary Fig. 3).

### Antimicrobial resistance characterization by agar diffusion test

We first assessed the antimicrobial resistance profile of each strain in pMBA collection using disc diffusion tests in MH-agar broth. Overnight cultures of pMBA-derived strains were adjusted to a 0.5 in the McFarland scale using saline solution (NaCl 0.9%). The resulting solutions were diluted 1:200 and plated on MH agar plates. Antibiotic discs (Supplementary Table 3) (Oxoid and BioRad) were then deposited on top of the bacterial lawn and plates were incubated overnight at 37 °C overnight. The resulting inhibitory halos around the antibiotic discs were then measured to determine the antibiotic resistance of each strain.

### Minimal inhibitory concentration determination

The MIC of pMBA collection strains against selected antimicrobial molecules (for a list of compounds see Supplementary Table 5) was determined for each pMBA derivate. MIC determination was performed according to the CLSI guidelines^62^. Briefly, 10^5^ colony-forming units (CFUs) were inoculated in 200 μl of fresh MH with doubling dilutions of each selected antibiotic in 96-well plates (Nunc) and incubated overnight at 37 °C in static conditions. The MIC was established as the lowest concentration in which growth could not be observed. MICs were measured at least in three biological replicates for each antibiotic and strain. Because *E. coli* MG1655 is intrinsically resistant to sulfamethoxazole (MIC = 1024 μg/mL), pMBA and pMBA carrying *qacEΔsul1* were introduced in *E. coli* DH5α, and MICs were determined in this genetic background.

### Reporting summary

Further information on research design is available in the Nature Research Reporting Summary linked to this article.

## Supplementary information

Supplementary Material

Reporting Summary

## Data Availability

All data reported in the manuscript are either represented in the figures or in Supplementary Material.
