# Supplementary material for: Profile and resistance levels of 136 integron resistance genes
Source: NPJ Antimicrob Resist. 2023 Oct 11;1:13. doi: 10.1038/s44259-023-00014-3 (PMC11721406; doi:10.1038/s44259-023-00014-3)

Supplementary Material.

Supplementary Table 1. Strains and plasmids used in this study.

All strains constructed for this work were built in an *E. coli* MG1655 or DH5α backgrounds (references A072 and A093 in lab collection).

Strain	Genetic background	Plasmid	ARC cloned	Source
A249	MG1655	pMBA empty vector	-	This study
C381	DH5α	pMBA empty vector	-	This study
A223	MG1655	pMBA *dfrA1*	*dfrA1*	This study
A224	MG1655	pMBA *dfrA5*	*dfrA5*	This study
A225	MG1655	pMBA *dfrA6*	*dfrA6*	This study
A226	MG1655	pMBA *dfrA7*	*dfrA7*	This study
A241	MG1655	pMBA *dfrA12*	*dfrA12*	This study
A227	MG1655	pMBA *dfrA14*	*dfrA14*	This study
A228	MG1655	pMBA *dfrA15*	*dfrA15*	This study
A229	MG1655	pMBA *dfrA16*	*dfrA16*	This study
A230	MG1655	pMBA *dfrA17*	*dfrA17*	This study
A231	MG1655	pMBA *dfrA21*	*dfrA21*	This study
A500	MG1655	pMBA *dfrA22*	*dfrA22*	This study
A232	MG1655	pMBA *dfrA25*	*dfrA25*	This study
A233	MG1655	pMBA *dfrA27*	*dfrA27*	This study
A613	MG1655	pMBA *dfrA29*	*dfrA29*	This study
A248	MG1655	pMBA *dfrA30*	*dfrA30*	This study
A234	MG1655	pMBA *dfrA31*	*dfrA31*	This study
A235	MG1655	pMBA *dfrA34*	*dfrA34*	This study
A242	MG1655	pMBA *dfrA35*	*dfrA35*	This study
A236	MG1655	pMBA *dfrB1*	*dfrB1*	This study
A245	MG1655	pMBA *dfrB2*	*dfrB2*	This study
A246	MG1655	pMBA *dfrB3*	*dfrB3*	This study
A237	MG1655	pMBA *dfrB4*	*dfrB4*	This study
A238	MG1655	pMBA *dfrB5*	*dfrB5*	This study
A239	MG1655	pMBA *dfrB6*	*dfrB6*	This study
A240	MG1655	pMBA *dfrB7*	*dfrB7*	This study
A247	MG1655	pMBA *dfrB8*	*dfrB8*	This study
A243	MG1655	pMBA *dfrB9*	*dfrB9*	This study
A390	MG1655	pMBA *bla*BEL-1	*bla*$_{BEL-1}$	This study
A380	MG1655	pMBA *bla*GES-1	*bla*$_{GES-1}$	This study
A387	MG1655	pMBA *bla*IMP-2	*bla*$_{IMP-2}$	This study
A383	MG1655	pMBA *bla*IMP-31	*bla*$_{IMP-31}$	This study
A441	MG1655	pMBA *bla*OXA-1	*bla*$_{OXA-1}$	This study
A373	MG1655	pMBA *bla*OXA-2	*bla*$_{OXA-2}$	This study
A381	MG1655	pMBA *bla*OXA-5	*bla*$_{OXA-5}$	This study
A384	MG1655	pMBA *bla*OXA-9	*bla*$_{OXA-9}$	This study
A374	MG1655	pMBA *bla*OXA-10	*bla*$_{OXA-10}$	This study
A385	MG1655	pMBA *bla*OXA-20	*bla*$_{OXA-20}$	This study

A382	MG1655	pMBA *bla*OXA-21	*bla*OXA-21	This study
A395	MG1655	pMBA *bla*OXA-46	*bla*OXA-46	This study
A375	MG1655	pMBA *bla*OXA-118	*bla*OXA-118	This study
A391	MG1655	pMBA *bla*OXA-129	*bla*OXA-129	This study
A376	MG1655	pMBA *bla*OXA-198	*bla*OXA-198	This study
A803	MG1655	pMBA *bla*PBL-1	*bla*PBL-1	This study
A388	MG1655	pMBA *bla*VIM-1	*bla*VIM-1	This study
A396	MG1655	pMBA *bla*VIM-2	*bla*VIM-2	This study
A389	MG1655	pMBA *bla*VIM-7	*bla*VIM-7	This study
A327	MG1655	pMBA *aacA2*	*aacA2*	This study
A266	MG1655	pMBA *aacA3*	*aacA3*	This study
A328	MG1655	pMBA *aacA4*	*aacA4*	This study
A267	MG1655	pMBA *aacA7*	*aacA7*	This study
A268	MG1655	pMBA *aacA8*	*aacA8*	This study
A263	MG1655	pMBA *aacA16*	*aacA16*	This study
A543	MG1655	pMBA *aacA17*	*aacA17*	This study
A264	MG1655	pMBA *aacA27*	*aacA27*	This study
A256	MG1655	pMBA *aacA28*	*aacA28*	This study
A257	MG1655	pMBA *aacA29*	*aacA29*	This study
A270	MG1655	pMBA *aacA30*	*aacA30*	This study
B657	MG1655	pMBA *aacA31*	*aacA31*	This study
A272	MG1655	pMBA *aacA34*	*aacA34*	This study
A329	MG1655	pMBA *aacA35*	*aacA35*	This study
A258	MG1655	pMBA *aacA37*	*aacA37*	This study
A603	MG1655	pMBA *aacA38*	*aacA38*	This study
A273	MG1655	pMBA *aacA42*	*aacA42*	This study
C117	MG1655	pMBA *aacA43*	*aacA43*	This study
A265	MG1655	pMBA *aacA45*	*aacA45*	This study
A274	MG1655	pMBA *aacA47*	*aacA47*	This study
A259	MG1655	pMBA *aacA48*	*aacA48*	This study
B656	MG1655	pMBA *aacA49*	*aacA49*	This study
A260	MG1655	pMBA *aacA50*	*aacA50*	This study
A261	MG1655	pMBA *aacA51*	*aacA51*	This study
A326	MG1655	pMBA *aacA52*	*aacA52*	This study
B655	MG1655	pMBA *aacA54*	*aacA54*	This study
A262	MG1655	pMBA *aacA56*	*aacA56*	This study
A303	MG1655	pMBA *aacA59*	*aacA59*	This study
A302	MG1655	pMBA *aacA61*	*aacA61*	This study
A566	MG1655	pMBA *aacA64*	*aacA64*	This study
A331	MG1655	pMBA *aacAX*	*aacAX*	This study
A308	MG1655	pMBA *aacC1*	*aacC1*	This study
A304	MG1655	pMBA *aacC2*	*aacC2*	This study
B625	MG1655	pMBA *aacC3*	*aacC3*	This study
A320	MG1655	pMBA *aacC4*	*aacC4*	This study
A305	MG1655	pMBA *aacC5*	*aacC5*	This study
A306	MG1655	pMBA *aacC6*	*aacC6*	This study
A332	MG1655	pMBA *aacC11*	*aacC11*	This study
A309	MG1655	pMBA *aacC13*	*aacC13*	This study
A311	MG1655	pMBA *aadA1*	*aadA1*	This study
A333	MG1655	pMBA *aadA2*	*aadA2*	This study
A319	MG1655	pMBA *aadA4*	*aadA4*	This study

A313	MG1655	pMBA $_{aadA5}$	*aadA5*	This study
A321	MG1655	pMBA $_{aadA6}$	*aadA6*	This study
A397	MG1655	pMBA $_{aadA7}$	*aadA7*	This study
A315	MG1655	pMBA $_{aadA10}$	*aadA10*	This study
A312	MG1655	pMBA $_{aadA11}$	*aadA11*	This study
A322	MG1655	pMBA $_{aadA13}$	*aadA13*	This study
A316	MG1655	pMBA $_{aadA16}$	*aadA16*	This study
A318	MG1655	pMBA $_{aadA24}$	*aadA24*	This study
A604	MG1655	pMBA $_{aadA28}$	*aadA28*	This study
A314	MG1655	pMBA $_{aadA29}$	*aadA29*	This study
A398	MG1655	pMBA $_{aadA34}$	*aadA34*	This study
A292	MG1655	pMBA $_{aadB}$	*aadB*	This study
A422	MG1655	pMBA $_{aphA15}$	*aphA15*	This study
C116	MG1655	pMBA $_{aphA16}$	*aphA16*	This study
A293	MG1655	pMBA $_{sat2}$	*sat2*	This study
A368	MG1655	pMBA $_{arr2}$	*arr2*	This study
A334	MG1655	pMBA $_{arr5}$	*arr5*	This study
A363	MG1655	pMBA $_{arr6}$	*arr6*	This study
A440	MG1655	pMBA $_{arr7}$	*arr7*	This study
A364	MG1655	pMBA $_{arr8b}$	*arr8b*	This study
A335	MG1655	pMBA $_{catB2}$	*catB2*	This study
A336	MG1655	pMBA $_{catB3}$	*catB3*	This study
A365	MG1655	pMBA $_{catB5}$	*catB5*	This study
A625	MG1655	pMBA $_{catB6}$	*catB6*	This study
A393	MG1655	pMBA $_{catB10}$	*catB10*	This study
A338	MG1655	pMBA $_{ereA2}$	*ereA2*	This study
A423	MG1655	pMBA $_{ereA3}$	*ereA3*	This study
A657	MG1655	pMBA $_{fosC2}$	*fosC2*	This study
A339	MG1655	pMBA $_{fosE}$	*fosE*	This study
A624	MG1655	pMBA $_{fosF}$	*fosF*	This study
A354	MG1655	pMBA $_{fosG}$	*fosG*	This study
A355	MG1655	pMBA $_{fosH}$	*fosH*	This study
A356	MG1655	pMBA $_{fosI}$	*fosI*	This study
A626	MG1655	pMBA $_{fosK}$	*fosK*	This study
A621	MG1655	pMBA $_{fosL}$	*fosL*	This study
A622	MG1655	pMBA $_{fosM}$	*fosM*	This study
A627	MG1655	pMBA $_{fosN}$	*fosN*	This study
A337	MG1655	pMBA $_{smr1}$	*smr1*	This study
A359	MG1655	pMBA $_{smr2}$	*smr2*	This study
A366	MG1655	pMBA $_{smr3}$	*smr3*	This study
A367	MG1655	pMBA $_{qacE}$	*qacE*	This study
B091	MG1655	pMBA $_{qacE\Delta\text{-}sul1}$	*qacEΔ-sul1*	This study
C382	DH5α	pMBA $_{qacE\Delta\text{-}sul1}$	*qacEΔ-sul1*	This study
A323	MG1655	pMBA $_{qacF}$	*qacF*	This study
A357	MG1655	pMBA $_{qacG}$	*qacG*	This study
A324	MG1655	pMBA $_{qacH}$	*qacH*	This study
A623	MG1655	pMBA $_{qacK}$	*qacK*	This study
A325	MG1655	pMBA $_{qacL}$	*qacL*	This study
A358	MG1655	pMBA $_{qacM}$	*qacM*	This study
A086	MG1655	R388$_{dfrA5\text{-}aadB\text{-}bla_{VEB\text{-}1}}$	*dfrA5-aadB-bla*$_{VEB\text{-}1}$	*Souque et al*, 2021
A078	MG1655	R388$_{aadB\text{-}dfrA5\text{-}bla_{VEB\text{-}1}}$	*aadB-dfrA5-bla*$_{VEB\text{-}1}$	*Souque et al*, 2021

Supplementary Table 2. Antimicrobial resistance cassettes (ARC) sequences.
In silico predicted main ORFs of each antimicrobial gene are underlined. ARCs described in *E. coli* in the data bases are also underlined.

ARC	Sequence (5´ → 3´)
dfrA1	TTAACCTCTGAGGAAGAATTGTGAAACTATCACTAATGGTAGCTATATCGAAGAATGGAGTTATCGGGAATGGCCCTGAT<br>ATTCCATGGAGTGCCAAAGGTGAACAGCTCCTGTTTAAAGCTATTACCTATAACCAATGGCTGTTGGTTGGACGCAAGAC<br>TTTTGAATCAATGGGAGCATTACCCAACCGAAAGTATGCGGTCGTAACACGTTCAAGTTTTACATCTGACAATGAGAACG<br>TATTGATCTTTCCATCAATTAAAGATGCTTTAACCAACCTAAAGAAAATAACGGATCATGTCATTGTTTCAGGTGGTGGGG<br>AGATATACAAAAGCCTGATCGATCAAGTAGATACACTACATATATCTACAATAGACATCGAGCCGGAAGGTGATGTTTAC<br>TTTCCTGAAATCCCCAGCAATTTTAGGCCAGTTTTTACCCAAGACTTCGCCTCTAACATAAATTATAGTTACCAAATCTGG<br>CAAAAGGGTTAACAAGTGGCAGCAACGGATTCGCAAACCTGTCACGCCTTTTGTGCCAAAAGCCGCGCCAGGTTTGCGAT<br>CCGCTGTGCCAGGCG
dfrA5	TTAACCCGGAACCAAAATTGTGAAAGTATCATTAATGGCTGCAAAAGCGAAAAACGGAGTGATTGGTTGCGGTCCACAC<br>ATACCCTGGTCCGCGAAAGGAGAGCAGCTACTCTTTAAAGCCTTGACGTACAACCAGTGGCTTTTGGTGGGCCGCAAGAC<br>GTTCGAATCTATGGGAGCACTCCCTAATAGGAAATACGCGGTCGTTACTCGCTCAGCCTGGACGGCCGATAATGACAACG<br>TAATAGTATTCCCGTCGATCGAAGAGGCCATGTACGGGCTGGCTGAACTCACCGATCACGTTATAGTGTCTGGTGGCGGG<br>GAGATTTACAGAGAAACATTGCCCATGGCCTCTACGCTCCATATATCGACGATTGATATTGAGCCGGAAGGAGATGTTTT<br>CTTTCCGAATATTCCCAATACCTTCGAAGTTGTTTTTGAGCAACACTTTAGCTCAAACATTAACTATTGCTATCAAATTTGG<br>CAAAAGGGTTAACAAAGCTATGCAATTGACGGTAAAAAGCTTCGTTCGCTTCGCTTGCTACGCTTCTTACCGCAATTGATA<br>ACGGCG
dfrA6	TTAGCCCTCAGGAGGAAAAATGAAAATATCTCTTATGGCAGCTGTTTCCGAGAATGGAGTAATTGGCTCTGGATTGGATA<br>TACCTTGGCATGTACAAGGCGAGCAGCTCCTATTCAAAGCCATGACTTACAATCAATGGCTTCTAGTTGGTCGTAAAACCT<br>TCGACTCAATGGGTAAACTTCCGAATAGAAAATATGCAGTGGTTACTCGTTCTAAAATTATCTCGAATGACCCTGATGTTG<br>TGTATTTCGCAAGTGTTGAATCGGCATTAGCTTACCTAAACAATGCGACAGCACATATCTTTGTTTCTGGTGGTGGTGAAA<br>TATATAAAGCTTTAATCGATCAAGCAGATGTTATCCATCTTTCAGTGATTCACAAGCATATCTCTGGCGATGTGTTTTTTCC<br>TCCAGTTCCACAGGGCTTCAAGCAAACATTTGAGCAAAGTTTCAGTTCAAATATTGATTACACGTACCAAATTTGGGCAA<br>AGGGCTAACAATCTGTTTAAGAGTGATTCGCAACGCGTGGAATTTTTACTATGCGTTGCGTTTAGTGTTTAAGGTGGTATG<br>CGGAGGCTTCGGTATTGCGTTGCTCACACCTTAACAGGGCG
dfrA7	TTAGCCATTACGGGGGTTGAATTGAAAATTTCATTGATTTCTGCAACGTCAGAAAATGGCGTAATCGGTAATGGCCCTGAT<br>ATCCCATGGTCAGCAAAAGGTGAGCAGTTACTCTTTAAAGCGCTCACATATAATCAGTGGCTCCTTGTTGGAAGGAAAAC<br>ATTTGACTCTATGGGTGTTCTTCCAAATCGAAAATATGCAGTAGTGTCGAGGAAAGGAATTTCAAGCTCAAATGAAAATG<br>TATTAGTCTTTCCTTCAATAGAAATCGCTTTGCAAGAACTATCGAAAATTACAGATCATTTATATGTCTCTGGTGGCGGTC<br>AAATCTACAATAGTCTTATTGAAAAAGCAGATATAATTCATTTGTCTACTGTTCACGTTGAGGTTGAAGGTGATATCAATT<br>TTCCTAAAATTCCAGAGAATTTCAATTTGGTTTTTGAGCAGTTTTTTTTGTCTAATATAAATTACACATATCAGATTTGGAA<br>AAAAGGCTAACAAGTCGTTCCAGCACCAGTCGCTGCGCTCCTTGGACAGTTTTTTAAGTCGCGGTTTTATGGTTTTGCTGC<br>GCAAAAGTATTCCATAAAACCACAACTTAAAAACCGCCGCTGAACTCGGCG
dfrA12	TTAGCCATATGAACTCGGAATCAGTACGCATTTATCTCGTTGCTGCGATGGGAGCCAATCGGGTTATTGGCAATGGTCCTA<br>ATATCCCCTGGAAAATTCCGGGTGAGCAGAAGATTTTTCGCAGACTCACTGAGGGAAAAGTCGTTGTCATGGGGCGAAAG<br>ACCTTTGAGTCTATCGGCAAGCCTCTACCGAACCGTCACACATTGGTAATCTCACGCCAAGCTAACTACCGCGCCACTGG<br>CTGCGTAGTTGTTTCAACGCTGTCGCACGCTATCGCTTTGGCATCCGAACTCGGCAATGAACTCTACGTCGCGGGCGGAGC<br>TGAGATATACACTCTGGCACTACCTCACGCCCACGGCGTGTTTCTATCTGAGGTACATCAAACCTTCGAGGGTGACGCCTT<br>CTTCCCAATGCTCAACGAAACAGAATTCGAGCTTGTCTCAACCGAAACCATTCAAGCTGTAATTCCGTACACCCACTCCGT<br>TTATGCGCGTCGAAACGGCTAACCATTCCGTCAACGGGACGCCAAAATGCTGCGCATTTTGGTTCCCTCCGCTGCGCTCCG<br>GCTCTCGTTACGTCCAACG
dfrA14	TTAACCCAGGATGAGAACCTTGAAAGTATCATTGATAGCTGCGAAAGCGAAAAACGGCGTGATTGGTTGCGGTCCAGAC<br>ATACCGTGGTCCGCGAAAGGGGAGCAGCTACTTTTTAAAGCATTGACCTACAATCAGTGTCTTCTGGTGGGTCGCAAGAC<br>GTTTGAATCTATGGGCGCACTCCCCAATAGGAAATACGCGGTCGTTACCCGCTCAGGTTGGACATCAAATGATGACAATG<br>TAGTTGTATTTCAGTCAATCGAAGAGGCCATGGACAGGCTAGCTGAATTCACCGGTCACGTTATAGTGTCTGGTGGCGGA<br>GAAATTTACCGAGAAACATTACCCATGGCCTCTACGCTCCACTTATCGACGATCGACATCGAGCCAGAGGGGGATGTTTT<br>CTTCCCGAGTATTCCAAATACCTTCGAAGTTGTTTTTGAGCAACACTTTACTTCAAACATTAACTATTGCTATCAAATTTGG<br>AAAAAGGGTTAACAAAGCTATGCAATCGACGGCAAAAAGCTTCGTTCGCTTCGCGCACTACGCTTTTTCCGCGATTGATA<br>GCGACG
dfrA15	TTAACCCCTAAGGAAGTATCGTGAAACTATCACTAATGGCAGCAATTTCGAAGAATGGAGTTATCGGAAATGGCCCAGAT<br>ATTCCATGGAGTGCCAAAGGGGAACAATTACTCTTCAAAGCGATTACCTATAATCAGTGGCTTTTGGTAGGCCGAAAGAC<br>TTTCGAGTCAATGGGGGCTTTACCCAACCGAAAATATGCCGTTGTAACTCGTTCAAGCTTCACTTCCAGTGATGAGAATGT<br>ATTGGTATTTCCATCTATCGATGAAGCGCTAAATCATCTGAAGACGATAACGGATCATGTGATTGTGTCTGGTGGTGGTGA<br>AATATACAAAAGCCTGATCGATAAAGTTGATACTTTACATATTTCAACAATCGACATTGAGCCAGAAGGTGATGTCTATTT<br>TCCAGAAATCCCCAGTAGTTTTAGGCCAGTTTTTAGCCAAGACTTCGTGTCTAACATAAATTATAGTTACCAAATCTGGCA<br>AAAGGGTTAACAAGTGGCAGCAACTGACCGCCAAAAGTGTCACTTGTTTTGCCAAAAAGCCGGCAAAACAAGCGCCAAT<br>TTTGTCGGCAGCTGTGCCAGGCG
dfrA16	TTAACTCGAGGGAGAAATCGTGAAGTTATCACTAATGGCTGCCAAGTCGAAGAACGGTATTATCGGTAATGGACCAGATA<br>TTCCATGGAGCGCCAAAGGCGAGCAACTTCTATTTAAGGCAATTACATATAATCAATGGCTTTTAGTTGGACGCAAAACT<br>TTTGAGTCAATGGGCGCTCTCCCAAATCGAAAGTATGCAGTTGTAACTCGCTCTAATTTTTCTACGAATGATGAGGGTGTA<br>ATGGTTTTCTCCTCAATTCAGGATGCCTTAATAAATTTAGAGGAAATCACGGATCATGTTATCGTTTCTGGTGGTGGTGAA<br>ATATACAAAAGCTTGATTTCCAAAGTAGATACTTTGCATATTTCAACAGTCGACATCGAGCGAGATGGAGACATAGTTTTT<br>CCTGAAATCCCAGATACATTCAAGTTGGTATTTGAGCAAGATTTCGAGTCTAACATTAACTATTGTTATCAAATCTGGCAA<br>AAGAGTTAACAAGCGCCTGCAATCTGACCTCCGGTTACTGTCACCTTTTTTTGCGGTGGAGCTGCAAAAAAGGCGCCATT<br>AACCTCCGGCAGTTGAGGCGGGCG
dfrA17	TTAGCCATTAAGGGAGTTAAATTGAAAATATCATTGATTTCTGCAGTGTCAGAAAATGGCGTAATCGGTAGTGGTCCTGA<br>TATCCCGTGGTCAGTAAAAGGTGAGCAACTACTCTTTAAAGCGCTCACATATAATCAATGGCTCCTTGTCGGAAGAAAAA<br>CATTTGACTCTATGGGTGTTCTTCCAAATCGCAAATATGCAGTAGTGTCAAAGAACGGAATTTCAAGCTCAAATGAAAAC<br>GTCCTAGTTTTTCCTTCAATAGAAAATGCTTTGAAAGAGCTATCAAAAGTTACAGATCATGTATATGTCTCTGGCGGGGGT<br>CAAATCTATAATAGCCTTATTGAAAAAGCAGATATAATTCATTTGTCTACTGTTCACGTTGAAGTCGAAGGTGATATCAAA<br>TTCCCTATAATGCCTGAGAATTTCAATTTGGTTTTTGAACAGTTTTTTATGTCTAATATAAATTATACATACCAGATTTGGA<br>AAAAAGGCTAACAATGCGTTGCAGCACCAGTCGCTTCGCTCCTTGGACAGCTTTTAAGTCGCGTCTTTGTGGTTTTGCTGC<br>GCAAAAGTATTCCACAAAGCCGCAACTTAAAAGCTGCCGCTGAACTTAACG
dfrA21	TTAGCCGTATGAACCCGGAATCGGTCCGCATTTATCTGGTCGCTGCCATGGGTGCCAATCGGGTTATTGGCAATGGTCCCG<br>ATATCCCCTGGAAAATCCCAGGTGAGCAGAAGATTTTTCGCAGGCTCACCGAGAGCAAAGTGGTCGTTATGGGCCGCAAG<br>ACATTTGAGTCCATAGGCAAGCCCTTACCAAACCGCCACACAGTGGTGCTCTCGCGCCAAGCTCGTTATAGCGCTCCTGG<br>TTGTGCAGTTGTTTCAACGCTGTCACAGGCTATCGCCATCGCAGCCGAACACGGCAAAGAACTCTACGTAGCCGGCGGAG<br>CCGAGGTATATGCGCTGGCGCTACCGCATGCCAACGGCGTCTTTCTATCTGAGGTACATCAAACCTTTGAGGGTGACGCCT

	TCTTCCCAGTGCTTAACGCAGCAGAATTCGAGGTTGTCTCATCCGAAACCATTCAAGGCACAATCACGTACACGCACTCC<br>GTCTATGCGCGTCGTAACGGCTAACAAGTCCGTCAACGGGACACCCAAATGCTGCGCATTTGGGTTCCCTTGGCTGCGCC<br>TCGGCGCCCGTTACGTCCAACG
dfrA22	TTAGCCGTATGAACCCGGAATTGGTCCGCATTTATCTGGTCGCTGCCATGGGTGCCAATCGGGTTATTGGCAATGGCCCCG<br>ATATTCCCTGGAAAATCCCGGGTGAGCAAAAGATCTTTCGCAGGCTCACCGAGGGCAAAGTGGTCGTTATGGGCCGCAAG<br>ACGTTTGAGTCCATAGGCAAGCCCTTACCAAACCGCCGCACAGTGGTGCTCTCGCGCCAAGCCAGTTATAGCGCTGCTGG<br>TTGTGCAGTTGTTTCAACGCTGTCGCAGGCTATTGCCATCGCAGCCGAACACGGCAAAGAGCTCTACGTGGCCGGCGGAG<br>CCGAGGTATATGCACTGGCACTACCTCGTGCCGACGGCGTCTTTCTATATGAGGTACATCAAACCTTCGAGGGTGACGCCT<br>TCTTCCCTGTGCTCGACGAAGCAGAATTCGAGGTTGTCTCAGCCGAAACCGTTCAAGCCACAATCACGTACACGCACTCC<br>GTCTATGCACGTCGTAACGGCTAACAAGTCCGTCAACGGGACACCCAAATGCTGCGCATTTGGGGTCCCTCGGCTGCGCC<br>TCGGTGCCCGTTACGTCCAACG
dfrA25	TTAACCCAGGACGAGTACCTTGAAAGTATCATTGATGGCTGCAAGAGCGAAAAATGGCGTAATCGGTTGCGGTCCTGACA<br>TTCCTTGGTCTGCCAAAGGGGAACAGCTTCTTTTCAAAGCACTGACCTATAACCAATGGCTTTTGGTAGGGCGCAAAACAT<br>TTGAGTCTATGGGGCCGCTGCCCAATAGGAAATACGCGGTTGTTACCCGCTCAAACTGGACAGCGGCTAATGAAAACGTA<br>GTGGTTTTCCCGTCGATTGACGAAGCGATGGGTAGATTAGGCGAGATCACTGACCATGTCATCGTCGCCGGTGGTGGAGA<br>AATCTACCATGAAACGATACCCATGGCCTCTACTCTGCATGTGTCGACAATCGACGTTGAGCCAGAGGGAGACGTTTTCTT<br>TCCGAACATTCCTGGGAAGTTTGATGTCGTTTTTGAGCAACAATTTACATCAAACATTAACTATTGCTATCAAATCTGGCA<br>AAAGGGTTAACAAAGCTATGCAATTGACGGCAAAAAGCTTCGTTCGCTTCACTCACTACGCAATTTGCCGCACTTGATAG<br>CGGCG
dfrA27	TTAACCCAAAGGAGTATCGTGAAAATATCACTAATGGCTGCAAAAGCAAGAAATGGGGTTATTGGCTGCGGCTCGGATAT<br>CCCGTGGAACGCTAAAGGTGAGCAGCTGCTTTTTAAAGCAATAACTTACAATCAATGGCTCTTAGTCGGCCGTAAAACAT<br>TTGAGGCAATGGGGGCTCTCCCAAATAGAAAGTATGCAGTTGTCAGCCGCTCAGGATCGGTAGCTACTAACGATGATGTG<br>GTTGTGTTTCCATCTATAGAAGCAGCAATGAGGGAGCTAAAGACTCTTACGAACCATGTTGTTGTTTCTGGTGGTGGAGA<br>GATCTACAAGAGTCTGATCGCCCATGCCGACACGCTACATATCTCGACAATAGATTCCGAGCCAGAGGGCAATGTTTTCT<br>TTCCGGAAATCCCCAAAGAGTTCAATGTGGTGTTCGAGCAGGAATTTCATTCAAATATAAATTATCGCTATCAAATCTGGC<br>AAAGGGGTTAACCATCCAAGCCATCGGACACATTTTGCTTCGCTGCGCTCAAAACGCAAAATGTGCCGCTGCTTAGCGGC<br>G
dfrA29	TTAACCATTACGGGAATTTAATTGAAAATTTCTTTAATCGCAGCTCAGTCAGAAAACGGTGTTATTGGTAATGGCCCAGAT<br>ATTCCATGGTCAGCAAAAGGGGAGCAGTTACTTTTCAAAGCGCTAACATATAATCAGTGGCTTCTTGTCGGAAGAAAAAC<br>ATTTGAGTCAATGGGTATTCTTCCTAATCGAAAGTATGCTGTTCTTTCAAAAAATGGAATTTCACACCTTCCTGAAAACGT<br>ACTAGTTTTTCGTCTATAGAAAATGCATTATATGAACTGGCTAAGGTAACAGACCATTTATATATTTCTGGCGGTGGTCA<br>AATATATAATAGTCTTATTGAAAGTGCTGATACCATCCACTTATCTATCATCCACAAAGAGGTAGAAGGTGAAGTAAGGT<br>TTCCCAAAATACCTCCTAATTACAAGTTGGTATTTGAGCAATATTATTCTTCAAATATTAATTACACTTATCAAATTTGGCA<br>AAAAGGTTAACAAGTCGCTCAAGCACCAGTCGCTTCGCTCCTTGGACAGTTTTTAAGTCGCAGTTTTGTGGTTTTGCTGCG<br>CAAAAAGTTTCCACAAAACCACAACTTAAAAACTGCCGCTTAGCTCGGCG
dfrA30	TTAACCCGGGACCAAAATTGTGAAAGTATCATTAATGGCTGCAAGAGCGAGAAACGGAGTGATCGGTTGCGGTCCACAC<br>ATACCCTGGTCCGCGAAAGGAGAGCAGCTACTCTTTAAAGCCCTGACGTACAACCAGTGGCTTTTGGTTGGCCGCAAGAC<br>GTTCGAATCAATGGGGGCGCTCCCCAACAGGAAATACGCGGTCGTTACTCGCTCAGCCTGGACGGCCAATAATGACAACG<br>TAGTAGTATTCCCGTCGATCGAAGAGGCCATGGGCGGTCTAGCTAAACTCAACGGTCACGTTATAGTGTCTGGTGGCGGG<br>GAGATTTACAGAGAAACGTTGCCCATGGCCTCTACGCTCCATGTATCGACGATCGACATTGAGCCAGAAGGGGATGTTTT<br>CTTCCCGAATATTCCCAACTTCTTCGAAGTTGTTTTTGAGCAACATTTTAGTTCAAACATTAACTATTGCTATCAAATTTGG<br>AAAAAGGGTTAACAAAGCTATGCAATTGACGGCAAAAAGCTTCGTTCGCCGCGCTCACTACGCTTTTACCGCAATTGAT<br>AGCGGCG
dfrA31	TTAGCCCTCAGGAGGAAAAATGAAAATATCCATTATGGCAGCAGTTTCTGAGAATGGAGTAATTGGCTCTGGATTGGATA<br>TACCTTGGCATGTACAAGGTGAGCAGCTCCTGTTCAAAGCTATGACTTACAATCATTGGCTTTTAGTCGGTCGTAAAACTT<br>TCGACTCAATGGGTAAACTTCCCAATAGGAAATATGCTGTGGTTACTCGCTCAGAAATGGTCTCGAATGATCCAGATGTT<br>ATTTATTTCACCAGCATTGAATCGGCATTATCTTACTTAGACAATACGACAACACATGTCTTTGTTTCTGGTGGTGGTGAA<br>ATTTACAAAGCATTAATCGAACAAGCAGATGTTATCCATCTTTCAGTGATTCATAAGCACATCTCTGGCGACGTGTTTTTC<br>CCTTCAGTTCCACAGAGTTTCAAACAAACATTTGAGCAAAGTTTCAGTTCAAATATTGATTACACGTACCAAATTTGGGCA<br>AAGGGCTAACAAACTGTTTAAGAGGGATTCACAACGCGTGGCATTTTTACTATGCGTTGGTTTTAGTGGTTAAGGCGGTAT<br>GCGGAAGCTTCGGTATTGCGTTGCTCACCCCTTAACAGGGCG
dfrA32	TTAGCCATATCGGGAGTTAAATTGAAAATTTCATTGATTTCTGCAGTGTCAGAAAATGGCGTAATCGGTAGTGGTCCTGAT<br>ATTCCGTGGTCAGCAAAAGGTGAGCAGCTAATCTTTAAGGCGCTCACATACAATCAGTGGCTTCTTGTTGGAAGGAAAAC<br>ATTTGACTCTATGGGAGTTCTTCCAAATCGCAAATATGCAGTAGTGTCAAAGAATGGAATTTCAGGGTCAAATGAAAACG<br>TCTTGGTTTTTCCTTCAATAGAAAATGCTTTGCAAGAACTATCTAAAATTACAGATCATGTATATATTTCGGGTGGGGGGC<br>AAATCTATGAAAGCCTTATTGAAAAAGCAGATATAATTCATCTATCTACTATTCATGTTGAGGTTGAAGGTGATATTAAAT<br>TCCCTATATTACCTGAAGGTTTCAACTTGGTTTTTGAACAGTTTTTTGTGTCTAATATAAATTATACATATCAAATTTGGAA<br>AAAAGGCTAACAAGTCGTTGCAGCACCAGTCGCTCCGCTCCTTGGACAGTTTTTAAGTTGTGGTTTTATGGTTTTGCTGCG<br>CAAAAATATTCCATAAAACCACAACTTAAAAACTGCCGCTGAACTCGGCG
dfrA34	TTAACCCAGGACCAAAACCTTGAAAGTATCATTAATGGCTGCAAGAGCGAAAAACGGAGTGATCGGTTGTGGTCCAGAC<br>ATACCCTGGTCCGCGAAAGGAGAGCAGCTACTTTTCAAAGCCTTGACGTACAATCAGTGGCTTTTGGTGGGCCGCAAGAC<br>GTTCGAATCTATCGGGGCGCTCCCTAATAGGAAGTACGCGGTCGTTACCCGCTCAGACTGGACAATGGATCGTGACGATG<br>TAGTAGTATTCCGGTCAATCGAAGAGGCGATGGACGGGCTAGCTAAACACACCAGTCACGTTATAGTGTCTGGTGGTGGG<br>GAGATTTACAGAGAAACCTTGCCGATAGCCTCTACCCTCCACGTATCGACAATCGACATTGAGCCAGAGGGAGACGTTTT<br>CTTCCCGAATATTCCGAATACCTTCGACGTTGTTTTTGAGCAACACTTTACTTCAAATATTAATTACTGCTATCAAATTTGG<br>CAAAAAGGGTTAACAAAGCTATGCAATTGACGGCAAAAAGCTTCGTTCGCTTCACTCACTACGCCTTTTGCCGCAATTGA<br>TAGCGGCG
dfrA35	TTAACTAGGAGAAATCGTTAAACTATCACTAATGGCAGCAATATCGAAGAATGGAGTCATCGGAAATGGCCCAGATATTC<br>CATGGAGTGCCAAAGGGGAACAATTACTGTTTAAGGCGATTACCTATAATCAATGGCTTTTGGTAGGTCGAAAGACTTTC<br>GAATCTATGGGAGCTTTACCCAATCGAAAGTATGCCGTTGTAACTCGTTCAAGCTTCACATCAAATGAAGAGAATGTATT<br>GGTATTTCCATCTATCGAAGATGCGCTAAACCAATTAGAGAAAATAACGGATCATGTGATTGTGTCTGGTGGTGGTGAAA<br>TATACAAAAGCCTAATATCTAAAGTAGATACATTACATATCTCTACCATTGATATTGAGCCTGAAGGTGATGTTGTTTTTC<br>CTAAAATTCCAGATTCATTTAAGTTAGTATTTAGCCAGGAATTCGAGTCTAACATTAATTATTGTTACCAAATCTGGCAGA<br>TGGGTTAACAAGGGCCTGCAATCTGACCTCCGGCCACTGTCACCTTTTGTGCAAAAAGCCGCACAAAAGGCGCCATTGGC<br>CTCCGGCAGTTGAGGCGGGCG
dfrB1	TTGGGCGTGACCAATGCAGCGTGTCGTCGGGCCACATAGAACACCTAGAAGTTCACAAGAAAGGTCGGAAATGGAACGA<br>AGTAGCAATGAAGTCAGTAATCCAGTTGCTGGCAATTTTGTATTCCCATCGAACGCCACGTTTGGTATGGGAGATCGCGT<br>GCGCAAGAAATCCGGCGCCGCCTGGCAAGGTCAGATTGTCGGGTGGTACTGCACAAATTTGACCCCCGAAGGCTACGCC<br>GTCGAGTCTGAGGCTCACCCAGGCTCAGTACAGATTTATCCTGTTGCGGCGCTTGAACGCATCAACTGAGTTCAAGGTTG<br>AAGTGCGCCGCTCAACGGTCAAACCGGGCATCACCGACTACAGCCCAACTTGTCGCTCCAGCGGACGGCTTCGCCGCCCG<br>CTGAGCTAATTCG
dfrB2	TTAGGCAGCCGTTGTGCTGGTGCTTTCTGATAGTTGTTGTGGGGTAGGCAGTCAGAGTTCGATTTGCTTGTCGCCATAATA<br>GATTCACAAGAAGGATTCGACATGGGTCAAAGTAGCGATGAAGCCAACGCTCCCGTTGCAGGGCAGTTTGCGCTTCCCCT<br>GAGTGCCACCTTTGGCTTAGGGGATCGCGTACGCAAGAAATCTGGTGCCGCTTGGCAGGGTCAAGTCGTCGGTTGGTATT<br>GCACAAAACTCACTCCTGAAGGCTATGCGGTCGAGTCCGAATCCCACCCAGGCTCAGTGCAAATTTATCCTGTGGCTGCA<br>CTTGAACGTGTGGCCTAACAATTCGCTCCAGCGGACGGCTTCGCCGCCCGCTGAGCTTCATCG

dfrB3	TTGGGCTTCACCGACGCAGCGTTGTCGGGCTAGCAAGCATCTCAAGAAATTCACAAGAAAGGTCAGATATGGACCAACAC<br>AACAATGGAGTCAGTACTCTAGTTGCTGGCCAGTTTGCGCTCCCATCGCACGCCACGTTTGGCCTGGGAGATCGCGTGCG<br>CAAGAAATCTGGCGCCGCTTGGCAGGGTCAAGTTGTCGGGTGGTACTGCACAAAACTGACCCCTGAAGGCTATGCCGTCG<br>AGTCCGAGTCTCACCCCGGTTCAGTACAGATTTACCCTGTGGCTGCGCTTGAACGCGTGGCCTGAATTCACGGTTGAGTGC<br>ACCGCTCAGCGCTCAAGCCGTGCAGTACCGCCCACAGCCCAACTGGTCGCTCCAGCGGACGGCTTCGCCGCCCGCTGAGC<br>TAGAGCG
dfrB4	TTGGGCTTCACCAGAGTATCAAGTTGCCGAGCTGGCGATAACCTAAAACATTAAAAAGAGGTTTGAAATGAATGAAGGA<br>AAAAATGAGGTCAGTACTTCAGCTGCTGGCCGGTTCGCATTCCCATCAAACGCCACGTTTGCCTTGGGGGATCGCGTACG<br>CAAGAAGTCTGGCGCTGCTTGGCAGGGGCGCATTGTCGGGTGGTACTGCACAACACTTACCCCTGAAGGCTACGCCGTCG<br>AGTCCGAATCTCACCCAGGCTCAGTCCAGATTTATCCCATGACTGCGCTTGAACGGGTGGCCTGAAGTCACGGTTGAAAT<br>GCACCGCCTGACGGCCAAATCATGCGGCACCGTCCACAGCCCAACTAGTCGCTCCAGCGGATGGCTTCGCCGCCCGCTGA<br>GCTAATTCG
dfrB5	TTGGGCGTGACGATTGCAGCGTGTTGTCGGGCTACGCAGCAACCCTAGAAATTCAAAAGAAGGGTCATAAATGGACCAA<br>GGCAGAAGTGAAGTCAGTAATCCAGTTGCTGGCCAGTTTGCGTTCCCTTCAAACGCCGCGTTCGGAATGGGAGATCGCGT<br>GCGCAAGAAATCTGGCGCCGCTTGGCAAGGCCAGATTGTCGGGTGGTACTGCACAAAATTGACCCCTGAAGGGTACGCTG<br>TCGAGTCTGAGGCTCACCCTGGCTCGGTACAGATTTATCCTGTTGCGGCACTGGAACGCATCAACTGAGTTCAAGATTGA<br>AGTGCTCCACTCAACGGCCATACCGTGCATCACCGCCCACGGCCCAACTTGTCGCTCCAGCGGACGGCTTCGCCGCCCAC<br>TGAGCTAATTCG
dfrB6	TTGGGCGTGACCACAGCTGCGTGTTGTCCGGCTACATAGCACCCCTAAAATTCACAAGAAAGGTCAGAAATGGACCAAGG<br>TAGCAATGAAGTCATTAATCCAGTCGCTGGCCAGTTTGCGTCCCCATCGAACGCCACGTTTGGTATGGGAGATCGCGTGC<br>GCAAGAAATCTGGCGCCGCCTGGCAAGGTCAGATTGTCGGGTGGTACAGCACAAAGTTGACCCCTGAAGGCTACGCTGTC<br>GAGTCTGAGGCTCACCCTGGCTCGGTGCAGATTTATCCTGTTGCCGCGCTTGAACGCGTCAACTGAGTTCAAGGTTGAAGT<br>GCGCCACTCAAGGGCCAAACCGTGCATCACCGCCAAAGGCCCAACTTGTCGCTCCAGCGGACGGCTTCGCCGCCCGCTGA<br>GCTAATTCG
dfrB7	TTAGGCCCCGCAGGGGAGAGTCGGCGCTTTGAACTTCTTAGCCTGCGCAGAAAGTTGTAGTTCCCACGCCTAGCGCCGCA<br>GTTCCTGGCAGCTCACCGCGGATGCAACCAACGCTCACCCAGTCAAATGGTAGATATGGATCACCAGATCCCCTCAAGGC<br>ATCCACAAGAAAGGTTAGGTATGGATCAAAGTAGTAAGGAAGTCAGTTCTCCAGCTACTGACCAGTTTGCGCTCCCATTC<br>CGCGCCACGTTTGGCCTGGGAGATCGCGTACGCAAGAAATCTGGCGCCGCTTGGCAGGGTCAAGTTGTCGGCTGGTACAG<br>CACAAAACTAACCCCAGAAGGCTATGCCGTCGAGTCCGAGTCTCATCCGGGCTCTGTACAAATCTATCCTGTTGCCGCGC<br>TTGAACGCGTGGCCTAACCCTTCCATCGAGAGGACGTCACAAGGGCTACGCCCTTGCGCCGCCTCTCATGTCAAACG
dfrB8	TTGGGCATGACCACCGCAGCGTGTTGTCGGGCGATATTGCACCCCTAGAAATTCACAAGAGAGGTCAGAAATGGACCAA<br>GGTAGCAATGAAGTCGGTAATCCAGTTGCGGGCCAGTTTTCGTTCCCATCGAACGCCGCGTTTAGTATGGGAGATCGCGT<br>GCGCAAGAAATCGGGCGCCGCTTGGCAAGGTCAGATTGTCGGGTGGTACTGCACAAAGTTGACCCCTGAAGGCTACGCTG<br>TCGAGTCTGAGGCTCACCCTGGCTCGGTACAGATTTATCCTGTTGCGGCGCTTGAACGCATCAACGGAGTTCAAGGTTGA<br>AGTGCGCCACTCAACGGCCAAACCGTGCATCACCGCCCACGGCCCAACCTGTCGCTCCAGCGGACGGCTTCGCCGTCCGC<br>TGAGCTAATTCG
dfrB9	TTAGGCCCCGCAATGCGAACCTCTCGTGGTGCCAGACGATTTCGCGCATCCCGTCAGGAATTGAACAGTCGCCGGTGCCT<br>GAATTCAATCTCCCTACGACATTCAACGAGGCTGCCTGCCCACCACGCAGACCCCAGTGGATCATGGGCGAAAGACTTCA<br>AGGTCATCGACCAATCACGTAGTAAATGGACCAGTCGAAGGTCGCAAGACGATCACAAGAAAGGTTATGTATGAATCAA<br>AGTAGCAATTGCATCAGCACTCCAGTTGTTGGACAGTTTGCGCTGCCATTTCAACCCACGTTCGGCCTGGGAGATCGCGTA<br>CGCAAGAAGTCTGGCGCCGCTTGGCAAGGTAAAGTTGTCGGCTGGTACTGCACAAAATTAACCCCTGAAGGCTACGCGGT<br>CGAGTCCGAAGCTCATCCAGGCTCAGTGCAGATTTATCCTGTGGCTGCGCTTGAACGCGTGGCCTAACCCCTCAGTCGAG<br>GCGACAAATTGCAGCAAGCTGCAATTTGCGCCTCACCTCGAACG
bla$_{BEL-1}$	TTAGACGTAAGCCTATAATCTCTAGTATTTTGAAAATCGAGATCAAGATGAAACTGCTGCTCTACCCGTTATTGCTGTTCC<br>TTGTCATTCCAGCCTTTGCCCAGGCGGACTTTGAACATGCCATTTCAGATCTTGAGGCGCACAATCAAGCCAAGATCGGA<br>GTGGCCCTAGTTAGTGAAAATGGCAACCTGATTCAAGGGTATCGTGCGAATGAAAGGTTCGCGATGTGCTCAACTTTCAA<br>GTTGCCGTTGGCCGCTCTTGTTCTGAGTCGCATTGACGCTGGGGAAGAGAATCCTGAGCGCAAGCTTCATTACGATTCCGC<br>GTTCCTTGAAGAGTACGCCCCAGCCGCAAAACGGTATGTGGCAACTGGATATATGACTGTAACTGAGGCAATTCAATCCG<br>CCCTCCAACTCAGCGACAATGCCGCAGCTAACCTGCTGTTAAAAGAGGTTGGCGGCCCACCTTTATTGACAAAGTATTTC<br>CGTAGCCTGGGTGATAAAGTAAGTCGCCTTGATCGTATTGAACCGACTTTGAACACCAATACGCCCGGCGATGAAAGAGA<br>TACAACAACGCCCATGTCCATGGCACAGACTGTGTCAAAGCTGATTTTTGGAGACACGTTGACATATAAATCCAAGGGGC<br>AGCTAAGGCGATTACTCATCGGCAATCAGACCGGGGACAAAACCATTCGAGCTGGCTTGCCTGATTCATGGGTAACGGGT<br>GACAAGACAGGCTCGTGTGCGAATGGCGGCCGTAACGATGTGGCGTTTTTATAACCACTGCCGGAAAAAAATATGTTCT<br>TTCTGTATATACCAATGCACCTGAATTGCAAGGCGAGGAAAGGGCGTTATTAATTGCTTCTGTAGCAAAGTTAGCACGTC<br>AATATGTTGTTCACTGAATCCGCATACGTCTAACAATTCGCCTAAGCCGGACGCTCGCATGGCTCGCGCCGCTTGGCT
bla$_{BIM-1}$	TTAGAAAGGAATAAAAATGAGAATATCGTTAGCTTTATGCTTGCTTAGTATTTTAAATTTTGCTGTTGCAGAAGAGGTATT<br>GCCAGAAATAAAAGTTGAAAAGCTAGAAGAAGGAATCTATCTTCACACATCTTATCTAGAGTATCAGGGAAATATTTATG<br>AAAAACATGGGTTGGTAGTTATTGATGATCGTAAGGCATATATAATTGATACGCCAGTTTTGGCTATAGATACTGAGCGG<br>CTAGTAAAGTGGTTTGAAGAGCGTAACTTTACTATAGGGGCTAGTTTCTCAACACATTTTCATAGTGACAGTTCCGGCGGC<br>ATAGAATGGCTAAATAAAAATTCTATTCCTACATATGCATCTGAATTAACAAATGAACTTCTAAAAAAAGACGGCAAGGC<br>GCAAGCTAAAAACTCTTTTAATGCAGTTAGTTTTTGGCTAGTTAAAAACAAAATTGAAGTTTTTATCCGGGGCCAGGGCA<br>TACACAGGATAACGAGGTCGTGTGGATACCTAGTAAGAAAATATTATTCGGTGGTTGTTTTGTTAAGCCAGACGGTCTTG<br>GGTATTTGGGTGACGCAAATTTAGAAGCTTGGCCAAATTCTGCTAGAAAGTTAATGTCTAAATATAGCAACGCAAAACTG<br>GTTATTCCAAGCCATAGTGAAATAGGAAATGCATCACTCTTGAAGCGTACATGGGAGCAGGCTGTTAAAGGGCTAAATGA<br>CAGTAAAAAAACATCACAGCTCAGCAAATAAATTTCTAACAAGTCGCTCAAGCACCAGTCGCTACGCTCCTTGGACAGTT<br>TTTAAGTCGCAGTTTTGTGGTTTTGCTGCGCAAAAGTATTCCACAAAACCACAACTTAAAAACTGCCGCTTAGCTCGGCG
bla$_{CARB-1}$	TTAGCCATATTATGGAGCCTCATGCTTTTATATAAAATGTGTGACAATCAAAATTATGGGGTTACTTACATGAAGTTTTTA<br>TTGGCATTTTCGCTTTTAATACCATCCGTGGTTTTTGCAAGTAGTTCAAAGTTTCAGCAAGTTGAACAAGACGTTAAGGCA<br>ATTGAAGTTTCTCTTTCTGCTCGTATAGGTGTTTCCGTTCTTGATACTCAAAATGGAGAATATTGGGATTACAATGGCAAT<br>CAGCGCTTCCCGTTAACAAGTACTTTTAAAACAATAGCTTGCGCTAAATTACTATATGATGCTGAGCAAGGAAAAGTTAA<br>TCCCAATAGTACAGTCGAGATTAAGAAAGCAGATCTTGTGACCTATTCCCCTGTAATAGAAAAGCAAGTAGGGCAGGCAA<br>TCACACTCGATGATGCGTGCTTCGCAACTATGACTACAAGTGATAATACTGCGGCAAATATCATCCTAAGTGCTGTAGGT<br>GGCCCCAAAGGCGTTACTGATTTTTTAAGACAAATTGGGGACAAAGAGACTCGTCTAGACCGTATTGAGCCTGATTTAAA<br>TGAAGGTAAGCTCGGTGATTTGAGGGATACGACAACTCCTAAGGCAATAGCCAGTACTTTGAATAAATTTTTATTTGGTTC<br>CGCGCTATCTGAAATGAACCAGAAAAAATTAGAGTCTTGGATGGTGAACAATCAAGTCACTGGTAATTTACTACGTTCAG<br>TATTGCCGGCGGGATGGAACATTGCGGATCGCTCAGGTGCTGGCGGATTTGGTGCTCGGAGTATTACAGCAGTTGTGTGG<br>AGTGAGCATCAAGCCCCAATTATTGTGAGCATCTATCTAGCTCAAACACAGGCTTCAATGGAAGAGCGAAATGATGCGAT<br>TGTTAAAATTGGTCATTCAATTTTTGACGTTTATACATCACAGTCGCGCTGATAAGGCTAACAAGGCCATCAAGTTGACGG<br>CTTTTCCGTCGCTTGTTTTGTGGTTTAACGCTACGCTACCACAAAACAATCAACTCCAAAGCCGCAACTTATGGCGGCG
bla$_{CARB-4}$	TTAGCCTTATTTGAATACTTCACACTCTGATATAAGATGTAAGCCAATCAAAATGATGAGGTTATTACATGAAGCTTTTAC<br>TGGTATTTTCGCTTTTAATACCGTCTATGGTGTTTGCAAATAGTTCAAAGTTTCAACAGGTTGAACAAGATGCTAAGGTAA<br>TTGAAGCATCTCTTTCTGCGCATATAGGGATTTCTGTTCTTGATACTCAAACTGGAGAGTATTGGGATTACAATGGCAATC<br>AGCGTTTTCCTTTGACAAGTACTTTTAAAACAATAGCTTGTGCTAAATTATTATATGATGCTGAGCAAGGGGAAATAAACC<br>CTAAGAGTACAATTGAGATCAAAAAAGCAGATCTTGTGACCTATTCTCCCGTAATAGAAAAGCAAGTAGGACAAGCAAT<br>AACGCTCGATGATGCGTGTTTTGCAACTATGACGACAAGTGATAATGCAGCAGCAAATATCATCCTAAATGCCCTAGGAG

	GTCCTGAAAGCGTGACGGATTTTCTAAGACAAATCGGAGATAAAGAAACCCGTCTAGACCGTATTGAACCTGAATTAAAT<br>GAAGGCAAGCTTGGTGATTTGAGGGATACGACAACTCCTAATGCAATAGTGAATACTTTAAATGAATTATTATTTGGTTCC<br>ACATTGTCTCAAGATGGCCAGAAAAAATTAGAGTATTGGATGGTGAATAATCAAGTCACTGGTAATTTATTGCGGTCAGT<br>ATTGCCAGAGGGATGGAATATTGCGGATCGTTCAGGTGCTGGCGGATTTGGTGCTCGGAGTATTACAGCCGTTGTTTGGA<br>GTGAAGCTCAATCCCCAATCATAGTTAGTATCTATCTAGCGCAAACAGAGGCTTCAATAGCAGATCGAAATGATGCAATT<br>GTTAAAATTGGTCGTTCAATTTTTGAAGTTTATTCATCACAATCGCGTTGATAAGGCTAACAAAGCATTTAAGAGGGACAG<br>CCAACGCGTGGCACTTTTATTATGCGTTGGTTTTTGTGGTTACGGTGTTATGCGGTAAGTTGGTAGTAGCGTTGGCTGCCC<br>CTTAATGCGGCG
bla$_{\text{DIM-1}}$	TTAGAGGGAAAAATCGAATGAGAACACATTTTACAGCGTTATTACTTCTATTCAGCTTGTCTTCGCTTGCTAACGACGAGG<br>TACCTGAGCTAAGAATCGAGAAAGTAAAAGAGAACATCTTTTTGCACACATCATACAGTCGTGTGAATGGGTTTGGTTTG<br>GTCAGTTCAAACGGCCTTGTTGTCATAGATAAGGGTAATGCTTTCATTGTTGATACACCTTGGTCAGACCGAGATACAGAA<br>ACGCTCGTACATTGGATTCGTAAAAATGGTTATGAGCTACTGGGGAGTGTTTCTACTCATTGGCATGAGGATAGAACCGC<br>AGGAATTAAATGGCTTAATGACCAATCAATTTCTACGTATGCCACGACTTCAACCAACCATCTCTTGAAAGAAAATAAAA<br>AAGAGCCAGCGAAATACACCTTGAAAGGAAATGAGTCCACATTGGTTGACGGCCTTATCGAAGTATTTTATCCAGGAGGT<br>GGTCATACAATAGACAACGTAGTGGTGTGGTTGCCAAAGTCGAAAATCTTATTTGGCGGCTGTTTTGTGCGTAGCCTTGAT<br>TCCGAGGGGTTAGGCTACACTGGTGAAGCCCATATTGATCAATGGTCCCGATCAGCTCAGAATGCTCTGTCTAGGTACTC<br>AGAAGCCCAGATAGTAATTCCTGGCCATGGGAAAATCGGGGATATAGCGCTGTTAAAACACACCAAAAGTCTGGCTGAG<br>ACAGCCTCTAACAAATCAATCCAGCCGAACGCTAACGCGTCGGCTGATTGAGGCG
bla$_{\text{GES-1}}$	TTAGACGGGCGTACAAAGATAATTTCCATCTCAAGGGATCACCATGCGCTTCATTCACGCACTATTACTGGCAGGGATCG<br>CTCACTCTGCATATGCGTCGGAAAAATTAACCTTCAAGACCGATCTTGAGAAGCTAGAGCGCGAAAAAGCAGCTCAGATC<br>GGTGTTGCGATCGTCGATCCCCAAGGAGAGATCGTCGCGGGCCACCGAATGGCGCAGCGTTTTGCAATGTGCTCAACGTT<br>CAAGTTTCCGCTAGCCGCGCTGGTCTTTGAAAGAATTGACTCAGGCACCGAGCGGGGGGATCGAAAACTTTCATATGGGC<br>CGGACATGATCGTCGAATGGTCTCCTGCCACGGAGCGGTTTCTAGCATCGGGACACATGACGGTTCTCGAGGCAGCGCAA<br>GCTGCGGTGCAGCTTAGCGACAATGGGGCTACTAACCTCTTACTGAGAGAAATTGGCGGACCTGCTGCAATGACGCAGTA<br>TTTTCGTAAAATTGGCGACTCTGTGAGTCGGCTAGACCGGAAAGAGCCGGAGATGGGCGACAACACACCTGGCGACCTCA<br>GAGATACAACTACGCCTATTGCTATGGCACGTACTGTGGCTAAAGTCCTCTATGGCGGCGCACTGACGTCCACCTCGACC<br>CACACCATTGAGAGGTGGCTGATCGGAAACCAAACGGGAGACGCGACACTACGAGCGGGTTTTCCTAAAGATTGGGTTG<br>TTGGAGAGAAAACTGGTACCTGCGCCAACGGGGGCCGGAACGACATTGGTTTTTTAAAGCCCAGGAGAGAGATTACGCT<br>GTAGCGGTGTATACAACGGCCCCGAAACTATCGGCCGTAGAACGTGACGAATTAGTTGCCTCTGTCGGTCAAGTTATTAC<br>ACAACTCATCCTGAGCACGGACAAATAGTTGACGCCCGTCTAACAATTCGTTCAAGCCGACGTTGCTTCGTGGCGGCGCT<br>TGCGTGCTACGCTAAGCTTCGCACGCCGCTTGCCACTGCGCACCGCGGCTTAACTCAGGCG
bla$_{\text{GIM-1}}$	TTAGAAGGATGATTTCCAAATGAAAAATGTATTAGTGTTTTTAATATTACTTGTAGCGTTGCCAGCTTTAGCTCAGGGTCA<br>TAAACCGCTAGAAGTTATAAAAATTGAAGATGGAGTATATCTTCATACCTCCTTTAAGAATATTGAAGGCTATGGGTTAG<br>TTGATTCGAATGGGTTGGTAGTTCTGGATAATAATCAAGCCTATATTATCGACACACCTTGGTCTGAAGAAGACACGAAG<br>TTGTTATTATCCTGGGCGACTGACAGGGGATACCAGGTTATGGCTAGCATCTCAACTCATTCTCATGAAGATCGCACTGCT<br>GGTATCAAGTTGCTAAATTCAAAGTCAATTCCTACATACACATCAGAGTTAACTAAAAAGCTTCTTGCCCGTGAAGGAAA<br>GCCGGTTCCTACCCACTACTTTAAAGACGACGAATTCACACTGGGAAATGGGCTTATAGAGCTCTACTATCCAGGTGCTG<br>GGCATACAGAGGATAATATTGTTGCTTGGTTACCCAAAAGCAAAATACTATTTGGTGGCTGCCTCGTGAGGAGTCATGAG<br>TGGGAAGGCTTAGGTTACGTAGGCGACGCCTCAATTAGCTCTTGGGCTGACTCAATTAAAAATATTGTATCGAAAAAATA<br>TCCCATTCAAATGGTCGTTCCGGGGCATGGCAAAGTTGGAAGTTCAGATATATTAGATCACACCATTGATCTTGCTGAATC<br>AGCTTCTAACAAATTAATGCAACCGACCGCTGAAGCGTCGGCTGATTAAGGCG
bla$_{\text{IMP-1}}$	TTAGAAAAGGAAAAGTATGAGCAAGTTATCTGTATTCTTTATATTTTTGTTTTGCAGCATTGCTACCGCAGCAGAGTCTTT<br>GCCAGATTTAAAAATTGAAAAGCTTGATGAAGGCGTTTATGTTCATACTTCGTTTGAAGAAGTTAACGGGTGGGGCGTTG<br>TTCCTAAACATGGTTTGGTGGTTCTTGTAAATGCTGAGGCTTACCTAATTGACACTCCATTTACGGCTAAAGATACTGAAA<br>AGTTAGTCACTTGGTTTGTGGAGCGTGGCTATAAAATAAAAGGCAGCATTTCCTCTCATTTTCATAGCGACAGCACGGGC<br>GGAATAGAGTGGCTTAATTCTCGATCTATCCCCACGTATGCATCTGAATTAACAAATGAACTGCTTAAAAAAGACGGTAA<br>GGTTCAAGCCACAAATTCATTTAGCGGAGTTAACTATTGGCTAGTTAAAAATAAAATTGAAGTTTTTATCCAGGCCCGGG<br>ACACACTCCAGATAACGTAGTGGTTTGGTTGCCTGAAAGGAAAATATTATTCGGTGGTTGTTTTATTAAACCGTACGGTTT<br>AGGCAATTTGGGTGACGCAAATATAGAAGCTTGGCCAAAGTCCGCCAAATTATTAAAGTCCAAATATGGTAAGGCAAAA<br>CTGGTTGTTCCAAGTCACAGTGAAGTTGGAGACGCATCACTCTTGAAACTTACATTAGAGCAGGCGGTTAAAGGGTTAAA<br>CGAAAGTAAAAAACCATCAAAACCAAGCAACTAAATTTCTAACAAGTCGTTGCAGCACGCCACTACGTGGCTGGACAGTT<br>TGTAAGTTGCGCTTTTGTGGTTTGCTTCGCAAAGTATTCCACAACGCGCAACTTACAAACTGCCGCTGAACTTAGCG
bla$_{\text{IMP-2}}$	TTAGAAAAGGGCAAGTATGAAGAAATTATTTGTTTTATGTGTATGCTTCCTTTGTAGCATTACTGCCGCGGGAGCGCGTTT<br>GCCTGATTTAAAAATCGAGAAGCTTGAAGAAGGTGTTTATGTTCATACATCGTTCGAAGAAGTTAACGGTTGGGGTGTTG<br>TTTCTAAACACGGTTTGGTGGTTCTTGTAAACACTGACGCCTATCTGATTGACACTCCATTTACTGCTACAGATACTGAAA<br>AGTTAGTCAATTGGTTTGTGGAGCGCGGCTATAAAATCAAAGGCACTATTTCCTCACATTTCCATAGCGACAGCACAGGG<br>GGAATAGAGTGGCTTAATTCTCAATCTATTCCCACGTATGCATCTGAATTAACAAATGAACTTCTTAAAAAAGACGGTAA<br>GGTGCAAGCTAAAAACTCATTTAGCGGAGTTAGTTATTGGCTAGTTAAAAATAAAATTGAAGTTTTTATCCCGGCCCGG<br>GGCACACTCAAGATAACGTAGTGGTTTGGTTACCTGAAAAGAAAATTTTATTCGGTGGTTGTTTTGTTAAACCGGACGGTC<br>TTGGTAATTTGGGTGACGCAAATTTAGAAGCTTGGCCAAAGTCCGCCAAAATATTAATGTCTAAATATGTTAAAGCAAAA<br>CTGGTTGTTTCAAGTCATAGTGAAATTGGGGACGCATCACTCTTGAAACGTACATGGGAACAGGCTGTTAAAGGGCTAAA<br>TGAAAGTAAAAAACCATCACAGCCAAGTAACTAAATTTCTAACAAGGCGGTGAAACTCATTCCGCTTCGCTTCACTGGAC<br>GCCGCAAGCGGCGCCGTTTACCTTCGCG
bla$_{\text{IMP-4}}$	TTAGAAAAGGGAAAGTATGAGCAAGTTATCTGTATTCTTTATATTTTTGTTTTGTAGCATTGCTACCGCAGCAGAGCCTTT<br>GCCAGATTTAAAAATTGAAAAACTTGATGAAGGCGTTTATGTTCATACTTCGTTTGAAGAAGTTAACGGGTGGGGCGTTG<br>TTCCTAAACATGGTTTGGTTGTTCTTGTAGATGCTGAAGCTTATCTAATTGACACTCCATTTACGGCTAAAGATACTGAAA<br>AGTTAGTCACTTGGTTTGTGGAACGTGGCTATAAAATAAAAGGCAGTATTTCCTCTCATTTTCATAGTGACAGCACGGGCG<br>GAATAGAGTGGCTTAATTCTCAATCCATCCCCACGTATGCGTCTGAATTAACTAATGAGCTGCTTAAAAAAGACGGTAAG<br>GTTCAAGCTAAAAATTCATTTGGCGGGGTTAACTATTGGCTAGTTAAAAATAAAATTGAAGTTTTTATCCAGGCCCAGGA<br>CACACTCCAGATAACCTAGTAGTTTGGCTGCCTGAAAGGAAAATATTATTCGGTGGTTGTTTTATTAAACCGTACGGTCTA<br>GGTAATTTGGGTGACGCAAATTTAGAAGCTTGGCCAAAGTCCGCTAAATTATTAATATCCAAATATGGTAAGGCAAAACT<br>GGTTGTTCCAAGTCACAGTGAAGCTGGAGACGCATCACTCTTGAAACTTACATTAGAGCAGGCGGTTAAAGGGTTAAACG<br>AAAGTAAAAAACCATCAAAACTAAGCAACTAAATTTCTAACAAGTCGTTGCAGCATCGTGCGCTGCGCGCACTGGACAGT<br>TTTTAAGTCGCGGTTTTATGGTTTTGCTTCGCAAAAATATTCCATAAAACCACAACTTAAAAACTGCCGCTGAACTCGGCG
bla$_{\text{IMP-5}}$	TTAGAAAAGGGAAAGTATGAGCAAGTTATTTGTATTCTTTATGTTTTTGTTTTGTAGCATTACTGCCGCAGCAGAGTCTTT<br>GCCAGATTTAAAAATTGAGAAGCTTGACGAAGGCGTTTATGTTCATACTTCGTTTGAAGAAGTTAACGGTTGGGGTGTTGT<br>TCCTAAACACGGCTTGGTGGTTCTTGTAAATACTGAGGCCTATCTGATTGACACTCCATTTACGGCTAAAGATACTGAAAA<br>GTTAGTCACTTGGTTTGTGGAACGCGGCTATAAAATAAAAGGCAGTATTTCCTCTCATTTTCATAGCGACAGCACGGGCG<br>GAATAGAGTGGCTTAATTCTCAATCTATCCCCACGTATGCATCTGAATTAACAAATGAACTTCTTAAAAAAGACGGTAAA<br>GTACAAGCTAAAAATTCATTTAGCGGAGCTAGCTATTGGCTAGTTAAGAAAAAGATTGAAGTTTTTATCCTGGTCCAGG<br>GCACACTCCAGATAACGTAGTGGTTTGGCTACCTGAAAATAGAGTTTTGTTCGGTGGTTGTTTTGTTAAACCGTACGGTCT<br>AGGTAATTTGGGTGACGCAAATGTAGAAGCTTGGCCAAAGTCCGCCAAATTATTAATGTCCAAATATGGTAAGGCAAAAC<br>TGGTAGTTCCAAGTCACAGTGAAGTTGGAGACGCATCACTCTTGAAACGTACGTTAGAACAGGCGGTTAAAGGGTTAAAC<br>GAAAGTAAAAAACCATCAAAACCAAGTAACTAAATTTCTAACAAGTCGTTGCAGCACCGTTCCGCTTCGCTGCACTGGAC<br>AGTTTTTAAGTTGCAGTTTTATGGTTTGCTGCGCAAATATTTCCATAAAACCACAACTTAAAAACTGCCGCTGAACTCGGC<br>G

bla$_{IMP-9}$	TTAGAAAAAGGGAAAGTATGAGCAAGTTATTTGTATTCTTTATGTTTTTGTTTTGTAGCATTACTGCCGCAGGAGAGTCTT<br>TGCCAGATTTAAAAATTGAGAAGCTTGACGAAGGCGTTTATGTTCATACTTCGTTTGAAGAAGTTAACGGTTGGGGTGTTA<br>TTCCTAAACACGGCTTGGTGGTTCTTGTAAATACTGATGCCTATCTGATAGACACTCCATTTACTGCTAAAGATACTGAAA<br>ATTTAGTTAATTGGTTTGTTGAGCGCGGCTATAGAATAAAAGGCAGTATTTCCTCACATTTCCATAGCGACAGCACGGGTG<br>GAATAGAGTGGCTTAATTCTCAATCTATCCCCACGTATGCATCTGAATTAACAAATGAACTTCTTAAAAAAGACGGTAAG<br>GTACAAGCTAAATATTCATTTAGCGGAGTTAGCTATTGGCTAGTTAAGAAAAAGATTGAAGTTTTTTATCCTGGTCCAGGG<br>CACGCTCCAGATAACGTAGTGGTTTGGCTGCCTGAAAATAGAGTTTTGTTCGGTGGTTGTTTTGTTAAACCCTACGGTCTA<br>GGTAATTTGGGTGACGCAAATTTAGAAGCTTGGCCAAAATCCGCCAAATTATTAATGTCAAAATATAGTAAGGCAAAACT<br>GGTTGTACCAAGTCATAGTGACATAGGAGATTCGTCGCTCTTGAAGCTTACATGGGAGCAGACGGTAAAAGGATTCAATG<br>AAAGCAAAAAAAGTACCACTGCACATTAACCAAATTTCTAACAAGTCGCTCAAGCATCGCACCTTCGGTGCTGGACAGTT<br>TTTAAGTCGCGCTTTTGTGGTTTTGCTACGCAAAAGGTTTCCACAAAATCACAACTTAAAAACTGCCGCTTAGCTCGGCG
bla$_{IMP-11}$	TTAGAAAGGAGTAAGTATGAAAAAACTATTTGTTTTATGTATATTTTTGTTTTGTAGCATTACTGCCGCAGGAGCGTCTTT<br>GCCTGATTTAAAAATTGAGAAGCTTGAAGAGGGTGTTTATGTTCATACATCGTTTGAAGAAGTTAACGGCTGGGGTGTTG<br>TTTCTAAACACGGTTTGGTGGTTCTTGTAAATACTGACGCCTATCTGATTGACACTCCATTTACTGCTAAAGATACTGAAA<br>AGTTAGTCAATTGGTTTGTGGAGCGCGGCTATAAAATCAAAGGCAGTATTTCCTCACATTTCCATAGCGACAGCACGGGT<br>GGAATAGAGTGGCTTAATTCTCAATCTATTCCCACGTATGCATCTGTATTAACAAATGAACTTCTCAAAAAAGACGGTAA<br>GGTGCAAGCTAAAAACTCATTTAGCGGAGTTAGCTATTGGCTAGTTAAAAATAAAATTGAAGTTTTTTATCCAGGCCCAG<br>GGCACACTCAAGATAACGTAGTGGTTTGGCTACCTAAAAATAAAATCTTATTTGGTGGTTGTTTTGTTAAACCATATGGTC<br>TTGGTAATCTAGATGACGCAAATGTTGAAGCATGGCCACATTCGGCTGAAAAATTAATATCTAAGTATGGTAATGCAAAA<br>CTGGTTGTTCCAAGCCATAGTGACATAGGAGATGCGTCGCTCTTGAAGCTTACGTGGGAACAGGCGGTAAAAGGGCTTAA<br>TGAAAGCAAAAAAAGTAACACTGTTCATTAACCAAATTTCTAACAAGTCGCTGAAGCATCGCACCTTCGGTGCTGGACAG<br>TTTTTAAGTCGCGCTTTTGTGGTTTTGCTACGCAAAAGGTTTCCACAAAATCACAACTTAAAAACTGCCGCTTAGCTCGGC<br>G
bla$_{IMP-12}$	TTAGAAAAGGGAAAGTATGAAGAAATTATTTGTTTTATGCATTTTTTTGTTTTTAAGTATTACTGCCTCAGGTGAGGTTTTG<br>CCTGATTTGAAAATTGAGAAGCTTGAAGAGGGTGTTTATCTTCATACATCTTTTGAAGAGGTTAGCGGTTGGGGTGTTGTT<br>ACTAAACATGGTTTGGTAGTTCTTGTAAATAATGACGCCTATCTAATTGACACTCCATTTACAAATAAAGATACTGAAAAA<br>TTAGTTGCTTGGTTTGTAGGGCGCGGCTTTACAATAAAGGGAAGTGTTTCCTCACATTTTCATAGCGACAGTACGGGTGGA<br>ATAGAGTGGCTTAATTCTCAATCTATTCCCACGTATGCATCTGAGTTAACAAATGAACTTCTGAAAAAGAACGGTAAGGT<br>GCAAGCTACAAATTCATTTAGCGGGGTTAGTTATTGGCTAGTTAAAAATAAAATTGAAATTTTTTATCCCGGCCCAGGACA<br>TACTCAAGATAACGTAGTGGTTTGGCTACCTGAAAACAAAATTTTATTCGGTGGTTGTTTTGTTAAACCGGACGGTCTTGG<br>TAATTTGGATGACGCAAATTTAAAAGCTTGGCCAAAGTCCGCAAAAATATTAATGTCTAAATATGGTAAAGCAAAGTTAG<br>TTGTTTCAGGTCATAGTGAAATTGGGAACGCATCACTCTTGAAACTTACTTGGGAGCAGGCTGTTAAAGGGCTAAAAGAA<br>AGTAAAAAACCATTACTGCCAAGTAACTAATTTTCTAACAAGTCGTTGCAGCACCGTTCCTTCGCTCCACTGGACAGTTTT<br>TAAGTCGCGGTTTTATGGTTTGTGCGCAAAAGTATTTCATAAAACCACAACTTAAAAACTGCCGCTGAACTCGGCG
bla$_{IMP-13}$	TTAGAAAAGGAAAGGTATGAAGAAATTATTTGTTTTATGTGTATGCTTCTTTTGTAGCATTACTGCCGCAGGAGCGGCTTT<br>ACCTGATTTAAAAATCGAGAAGCTTGAAGAAGGTGTTTTTGTTCATACATCGTTCGAAGAGGTTAACGGTTGGGGGGTTG<br>TTACTAAACACGGTTTAGTGGTGCTTGTAAACACAGACGCCTATCTAATTGACACTCCATTTACTGCTACAGACACTGAAA<br>AATTAGTCAATTGGTTTGTGGAGCGCGGCTATGAAATCAAAGGCACTATTTCATCACATTTCCATAGCGACAGCACAGGA<br>GGAATAGAGTGGCTTAATTCTCAATCTATTCCCACGTATGCATCTGAATTAACAAATGAACTTTTGAAAAAATCCGGTAA<br>GGTACAAGCTAAATATTCATTTAGCGAAGTTAGCTATTGGCTAGTTAAAAATAAAATTGAAGTTTTCTACCCTGGCCCAGG<br>TCACACTCAAGATAACCTAGTGGTTTGGTTGCCTGAAAGTAAAATTTTATTCGGTGGTTGCTTTATTAAACCTCACGGTCT<br>TGGCAATTTAGGTGACGCAAATTTAGAAGCTTGGCCAAAGTCCGCCAAAATATTAATGTCTAAATATGGCAAAGCAAAGC<br>TTGTTGTTTCAAGTCATAGTGAAAAAGGGGACGCATCACTAATGAAACGTACATGGGAACAAGCCCTTAAAGGGCTTAAA<br>GAAAGTAAAAAAACATCATCACCAAGTAACTAAATTTCTAACAAGTCGTTGCAGCACCGCGCACTTCGTGCGCTGGACAG<br>TTTTTAAGTCGCGGCTTTATGGTTTTGCTGCGCAAAAGTATTCCATAAAACCACAACTTAAAAACTGCCGCTGAACTCGGC<br>G
bla$_{IMP-14}$	TTAGAAAAGGATAAGTATGAAAAAATTATTTGTTTTATGTGTATTCTTCTTCTGCAACATTGCAGTTGCAGAAGAATCTTT<br>GCCTGATTTAAAAATTGAGAAGCTTGAAGAAGGCGTTTATGTTCATACTTCGTTTGAAGAAGTTAAAGGTTGGAGTGTGG<br>TCACTAAACACGGTTTGGTGGTTCTTGTGAAAAATGACGCCTATCTGATTGATACTCCAATTACTGCTAAAGATACTGAAA<br>AATTAGTCAATTGGTTTGTTGAGCGGGGCTATAAAATCAAAGGCAGTATTTCAACACATTTCCATGGTGACAGTACGGCT<br>GGAATAGAGTGGCTTAATTCTCAATCTATCCCCACATATGCTTCTGAATTAACAAATGAACTTCTTAAAAAAGACAATAA<br>GGTACAAGCTAAACACTCTTTTAATGGGGTTAGTTATTCACTAATTAAAAACAAAATTGAAGTTTTTTATCCAGGCCCAGG<br>GCACACTCAAGATAACGTAGTGGTTTGGTTACCTGAAAAGAAAATTTTATTCGGTGGTTGCTTTGTTAAACCGGACGGTCT<br>TGGCTATTTGGGGGACGCAAATTTAGAAGCTTGGCCAAAGTCCGCTAAAATATTAATGTCTAAATATGGTAAAGCAAAAC<br>TAGTTGTGTCGAGTCATAGTGATATTGGAGATGTATCACTCTTGAAACGTACATGGGAGCAGGCTGTTAAAGGGCTGAAT<br>GAAAGTAAAAAATCATCACAGCCAAGCGACTAAATTTCTAACAAGGCGCTTCAGCACCGCGCACTTCGTGCGCTCGACAG<br>TTCGTAAGCCGCTTTTTTGTGGTTTTGCTACGCAAAAGGTTTCCACAAAAAATCAACTTACAAACTGCGGCTGAGCTTAAC<br>G
bla$_{IMP-15}$	TTAGAAAAGGTAAAGTATGAACAAGTTATCTGTATTCTTTATGTTTATGTTTTGTAGCATTACTGCCGCAGGAGAGTCTTT<br>GCCAGATTTAAAAATTGAGAAGCTTGACGAAGGTGTTTATGTTCATACTTCGTTTGAAGAAGTTAACGGTTGGGGTGTTGT<br>TCCTAAACACGGCTTGGTGGTTCTTGTAAATACTGAGGCCTATCTGATTGACACTCCATTTACGGCAAAAGATACTGAAAA<br>GTTAGTCACTTGGTTTGTGGAGCGCGGCTATAAAATAAAAGGCAGTATTTCCTCTCATTTTCATAGCGACAGCACGGGCG<br>GAATAGAGTGGCTTAATTCTCAATCTATCCCCACGTATGCATCTGAATTAACAAATGAACTTCTTAAAAAAGACGGTAAG<br>GTACAAGCTAAAAATTCATTTAGCGGAGGTAGCTATTGGCTAGTTAATAATAAGATTGAAGTTTTTTATCCTGGTCCAGGG<br>CACACTCCAGATAACGTAGTGGTTTGGCTACCTGAAAATAGAGTTTTGTTCGGTGGTTGTTTTGTTAAACCGTACGGTCTT<br>GGTAATTTGGGTGACGCAAATTTAGAAGCTTGGCCAAAGTCCGCCAAAATATTAATGTCTAAATATGGTAAAGCAAAGTT<br>GGTTGTTTCAAGTCATAGTGAAACTGGGAACGCATCACTCTTGAAACTTACTTGGGAGCAGGCTGTTAAAGGGCTAAAAG<br>AAAGTAAAAAACCATCACTGCCAAGTAACTAATTTTCTAACAAGTCGTTGCAGCACCGCTCCGGCACTTCGTGCCTGCGC<br>TGGACAGTTTTTAAGTCGCAGTTTTATGGTTTTGCTGCGCAAAAGTATTCCATAAAACCACAACTTAAAAACTGCCGCTGA<br>ACTCAGCG
bla$_{IMP-16}$	TTAGAAAAGGGCGAGTATGAAAAAATTATTTGTTTTATGTATCTTTTTGTTTTGTAGCATTACTGCCGCAGGAGAGTCTTT<br>GCCTGATTTAAAAATTGAGAAGCTTGAAGACGGTGTTTATGTTCATACATCGTTTGAAGAAGTTAACGGTTGGGGTGTTGT<br>TACTAAACACGGTTTGGTGTTTCTTGTAAACACAGACGCCTATCTGATTGACACTCCATTTGCTGCTAAAGACACTGAAAA<br>GTTAGTAAATTGGTTTGTGGAGCGCGGTTATAAAATAAAAGGCAGTATTTCCTCACATTTTCATAGCGACAGCTCGGGTG<br>GAATAGAATGGCTTAACTCTCAATCTATTCCCACGTATGCATCTGAATTAACAAACGAACTTCTTAAAAAGAACGGTAAG<br>GTGCAAGCTAAAAACTCATTTAGCGGAGTTAGTTATTGGCTACTTAAAAATAAAATTGAAATTTTTTATCCGGGCCCTGGG<br>CACACTCAAGATAACGTAGTGGTTTGGTTGCCTGAAAAGAAAATTTTATTTGGTGGGTGTTTTGTTAAACCGTACGGTCTT<br>GGAAATCTCGATGATGCAAATGTTGAAGCGTGGCCACATTCTGCTGAAATATTAATGTCTAGGTATGGTAATGCAAAACT<br>GGTTGTTCCAAGCCATAGTGACGTCGGAGATGCGTCGCTCTTGAAGCTTACATGGGAGCAGGCTGTTAAAGGGCTAAAAG<br>AAAGTAAAAAACCATCACAGCCAAGTAACTAATTTTCTAACAAGTCGCTCAAGCATCGCGCACTTCGTGCGCTGGACAGT<br>TTTTAAGTCGCAGTTTTGTGGTTTTGCTGCGCAAAAGTATTCCACAAAACTACAACTTAAAAACTGCCGCTTAGCTCGGCG
bla$_{IMP-18}$	TTAGAAAAGGGTAAGTATGAAAAAATTATTTGTTTTATGTGTATTCTTCCTTTGCAACATTGCTGCTGCAGATGATTCTTTG<br>CCTGATTTAAAAATTGAGAAGCTTGAAAAAGGCGTTTATGTTCATACTTCGTTTGAAGAAGTTAAAGGTTGGGGTGTAGT<br>CACAAAACACGGTTTAGTGGTTCTTGTAAAGAATGATGCTTATCTGATAGATACTCCAATTACCGCTAAAGATACTGAAA<br>AATTAGTTAATTGGTTTATTGAGCACGGCTATAGAATCAAAGGCAGTATTTCCACACATTTCCATGGCGACAGTACGGCTG<br>GAATAGAGTGGCTTAATTCTCAATCTATCTCCACGTATGCCTCTGAATTAACAAATGAACTTCTAAAAAAAGACAATAAG

	GTGCAAGCTACAAATTCTTTTAGTGGAGTTAGTTATTCACTTATCAAAAACAAAATTGAAGTTTTCTATCCAGGTCCAGGA<br>CACACTCAAGATAACGTAGTGGTTTGGTTACCTGAAAAGAAAATTTTATTCGGTGGTTGCTTTGTTAAACCGGACGGTCTT<br>GGAAATTTAGGGGATGCAAATTTAGAAGCTTGGCCAAAGTCCGCTAAAATATTAATGTCTAAATATGGTAAAGCAAAACT<br>GGTTGTTTCAAGTCATAGTGAAATTGGAAACGCATCACTCTTGCAGCGCACATGGGAGCAGGCTGTTAAAGGGTTAAATG<br>AAAGTAAAAAACCGTTACAGCCAAGTAGCTAAATTTCTAACAAGTCGTTGCAGCATCGTTCCGCTTTGCTCCACTGGACG<br>GGTTTTAAGTCGCATTTTTTGTGGCTCGCTGGCGCTCACTTTATCACAAAAATACAACTTAAAACCCGCCGCTGAACTCGG<br>CG
$\textit{bla}_{\text{IMP-22}}$	TTAGAAAAGGGGAAAGTATGAAGAAATTATTTGTTTTATGTGTGTTTTTGTTTTGTAGCATTACTGCCGCAGGAGAGTCTT<br>TGCCCGATTTAAAAATTGAAAAGCTTGAAGAAGGTGTTTATGTTCATACATCGTTTGAAGAAGTTAATGGTTGGGGCGTT<br>GTTTCTAAACACGGTTTGGTTATTCTTGTGAATACTGACGCCTATCTGATTGACACTCCATTCACGGCTAAAGATACTGAA<br>AAGTTAGTCACCTGGTTTGTGGAGCGCGGCTATAAAATCAAAGGTAGCATTTCCTCACATTTCCATAGCGACAGCACGGG<br>TGGAATAGAGTGGCTTAATTCTCAATCAATTCCCACGTATGCATCTGAATTAACAAATGACCTTCTTAAACAAAACGGTA<br>AGGTACAAGCTAAAAACTCATTTAGCGGAGTTAGTTATTGGTTAGTTAAAAATAAAATTGAAGTTTTCTATCCCGGCCCCG<br>GGCACACTCAAGATAACGTAGTGGTTTGGTTGCCTGAAAAGAAAATTTTATTTGGTGGGTGCTTTGTTAAACCGTACGGTC<br>TTGGAAATCTCGATGACGCAAATGTTGTAGCATGGCCACATTCTGCTGAAATATTAATGTCTAGGTATGGTAATGCAAAA<br>CTGGTTGTTCCAAGCCATAGTGACATCGGAGATGCGTCGCTCTTGAAGCTTACATGGGAGCAGGCTGTTAAAGGGCTAAA<br>AGAAAGTAAAAAACCATCAGAGCCAAGTAACTAATTTCTAACAAGTCGCTTAAGCATCAGTCGCTACGCTCCTTGGACAG<br>CTTTTAAGTCGCAGTTTTGTGGTTTTGCTGCGCAAAAGTATTCCACAAAACCGCAACTTAAAAACTGCCGCTTAGCTCGGC<br>G
$\textit{bla}_{\text{IMP-31}}$	TTAGAAAATGGTGCGTATGAAAAAAATATTTGTGTTATTTGTATTTTTGTTTTGCAGTATTACTGCCGCCGGAGAGTCTTTG<br>CCTGATATAAAAATTGAGAAACTTGACGAAGATGTTTATGTTCATACTTCTTTTGAAAAGATAACCGGCTGGGGTGTTATT<br>ACTAAACACGGCTTGGTGGTTCTTGTAAATACTGATGCCTATATAATTGACACTCCATTTACAGCTAAAGATACTGAAAAA<br>TTAGTCCGCTGGTTTGTGGGGCGTGGTTATAAAATCAAAGGCAGTATTTCCTCACATTTTCATAGCGATAGCGCAGGTGGA<br>ATTGAGTGGCTTAATTCTCAATCTATCCCCACATATGCATCTAAATTAACAAATGAGCTTCTTAAAAAGAACGGTAATGCG<br>CAAGCCGAAAACTCATTTAGTGGCGTTAGCTATTGGCTAGTTAAACATAAAATTGAAGTTTTCTATCCAGGACCAGGGCA<br>CACTCAGGATAATGTAGTGGTTTGGTTGCCTGAAAAGAAAATTTTATTTGGCGGTTGTTTTATTAAGCCGGACGGTCTTGG<br>TTATTTGGGAGACGCAAATCTAGAAGCATGGCCTAAGTCCGCAGAAACATTAATGTCTAAGTATGGTAATGCAAAACTGG<br>TTGTTTCGAGTCATAGTGAAATTGGGGGCGCATCACTATTGAAGCGCACTTGGGAGCAGGCTGTTAAGGGGCTAAAAGAA<br>AGTAAAAACCATCACAGCCCCAAATAACTAATTTCTAACAAGTCTCTCAAGCGGGACTGCCCCAACCGGCGCTGCTTTC<br>AGCATAGCGCCGGTTGCGTACGTTTCGCGCTGCGCGCTCCACTCAGCCCCTTAGCTCTGCG
$\textit{bla}_{\text{OXA-1}}$	TTGGGCGAACCCGGAGCCTCATTAATTGTTAGCCGTTAAAATTAAGCCCTTTACCAAACCAATACTTATTATGAAAAACAC<br>AATACATATCAACTTCGCTATTTTTTAATAATTGCAAATATTATCTACAGCAGCGCCAGTGCATCAACAGATATCTCTAC<br>TGTTGCATCTCCATTATTTGAAGGAACTGAAGGTTGTTTTTTACTTTACGATGCATCCACAAACGCTGAAATTGCTCAATTC<br>AATAAAGCAAAGTGTGCAACGCAAATGGCACCAGATTCAACTTTCAAGATCGCATTATCACTTATGGCATTTGATGCGGA<br>AATAATAGATCAGAAAACCATATTCAAATGGGATAAAACCCCCAAAGGAATGGAGATCTGGAACAGCAATCATACACCA<br>AAGACGTGGATGCAATTTTCTGTTGTTTGGGTTTCGCAAGAAATAACCCAAAAAATTGGATTAAATAAAATCAAGAATTA<br>TCTCAAAGATTTTGATTATGGAAATCAAGACTTCTCTGGAGATAAAGAAAGAAACAACGGATTAACAGAAGCATGGCTCG<br>AAAGTAGCTTAAAAATTTCACCAGAAGAACAAATTCAATTCCTGCGTAAAATTATTAATCACAATCTCCCAGTTAAAAAC<br>TCAGCCATAGAAAACACCATAGAGAACATGTATCTACAAGATCTGGATAATAGTACAAAACTGTATGGGAAAACTGGTG<br>CAGGATTCACAGCAAATAGAACCTTACAAAACGGATGGTTTGAAGGGTTTATTATAAGCAAATCAGGACATAAATATGTT<br>TTTGTGTCCGCACTTACAGGAAACTTGGGGTCGAATTTAACATCAAGCATAAAAGCCAAGAAAAATGCGATCACCATTCT<br>AAACACACTAAATTTATAAAAAATCTAATGGCAAAATCGCCCAACCCTTCAATCAAGTCGGGACGGCCAAAAGCAAGCTT<br>TTGGCTCCCCTCGCTGGCGCTCGGCGCCCCTTATTTCAAACG
$\textit{bla}_{\text{OXA-2}}$	TTGGGCATTAAGGAAAAGTTAATGGCAATCCGAATCTTCGCGATACTTTTCTCCATTTTTTCTCTTGCCACTTTCGCGCATG<br>CGCAAGAAGGCACGCTAGAACGTTCTGACTGGAGGAAGTTTTTCAGCGAATTTCAAGCCAAAGGCACGATAGTTGTGGCA<br>GACGAACGCCAAGCGGATCGTGCCATGTTGGTTTTTGATCCTGTGCGATCGAAGAAACGCTACTCGCCTGCATCGACATT<br>CAAGATACCTCATACACTTTTTGCACTTGATGCAGGCGCTGTTCGTGATGAGTTCCAGATTTTTCGATGGGACGGCGTTAA<br>CAGGGGCTTTGCAGGCCACAATCAAGACCAAGATTTGCGATCAGCAATGCGGAATTCTACTGTTTGGGTGTATGAGCTAT<br>TTGCAAAGGAAATTGGTGATGACAAAGCTCGGCGCTATTTGAAGAAAATCGACTATGGCAACGCCGATCCTTCGACAAGT<br>AATGGCGATTACTGGATAGAAGGCAGCCTTGCAATCTCGGCGCAGGAGCAAATTGCATTTCTCAGGAAGCTCTATCGTAA<br>CGAGCTGCCCTTTCGGGTAGAACATCAGCGCTTGGTCAAGGATCTCATGATTGTGGAAGCCGGTCGCAACTGGATACTGC<br>GTGCAAAGACGGGCTGGGAAGGCCGTATGGGTTGGTGGGTAGGATGGGTTGAGTGGCCGACTGGCTCCGTATTCTTCGCA<br>CTGAATATTGATACGCCAAACAGAATGGATGATCTTTTCAAGAGGGAGGCAATCGTGCGGGCAATCCTTCGCTCTATTGA<br>AGCGTTACCGCCCAACCCGGCAGTCAACTCGGACGCTGCGCGATAAAACCGCGCAGCGCCGGTTACTTCAACG
$\textit{bla}_{\text{OXA-5}}$	TTAGCCACCAAGGTACCATGAAAACCATAGCCGCATATTTAGTTCTAGTTTTTATGCAAGCACCGCGCTCTCAGAGTCTA<br>TTTCTGAAAATTTGGCGTGGAATAAAGAATTTTCTAGTGAATCCGTACATGGCGTTTTTGTACTTTGTAAAAGTAGTAGCA<br>ATTCCTGTACTACAAATAATGCGGCACGTGCATCTACAGCCTATATTCCAGCATCAACATTCAAAATTCCTAATGCTCTAA<br>TAGGTCTTGAAACCGGCGCCATAAAAGATGAACGGCAGGTTTTCAAATGGGACGGCAAGCCCAGAGCCATGAAGCAATG<br>GGAAAAAGACTTAAAGCTAAGGGGCGCTATACAGGTTTCTGCTGTTCCGGTATTTCAACAAATTGCCAGAGAAGTTGGCG<br>AAATAAGAATGCAAAAATACCTTAACCTGTTTTCATACGGCAACGCCAATATAGGGGGAGGCATTGACAAATTCTGGCTA<br>GAAGGTCAGCTTAGAATCTCAGCATTCAATCAAGTTAAATTTTTAGAGTCGCTCTACCTGAATAATTTGCCAGCATCAAAA<br>GCAAACCAACTAATAGTAAAAGAGGCAATAGTTACAGAAGCAACTCCAGAATATATAGTTCATTCAAAAACTGGGTATTC<br>CGGTGTTGGCACAGAATCAAGTCCTGGTGTCGCTTGGTGGGTTGGTTGGGTAGAGAAAGGAACTGAGGTTTACTTTTTGC<br>TTTTAACATGGACATAGACAATGAGAGTAAATTGCCGTCAAGAAAATCCATTTCAACGAAAATCATGGCAAGTGAAGGC<br>ATCATCATTGGTGGCTAACAAGGCGCTCAAGGCTCGGTTCGCCACTGGACGTCTCCAATCGGCGCATCGTTCGCATTTTAC<br>GCGCCGCTTGTAGCCGCCCCTTAGCTTTGCG
$\textit{bla}_{\text{OXA-9}}$	TTATGCACCTATTAAGCGCACAGCGGAGCAATGAAGGATACCTTGATGAAAAAAATTTTGCTGCTGCATATGTTGGTGTT<br>CGTTTCCGCCACTCTCCCAATCAGTTCCGTGGCTTCTGATGAGGTTGAAACGCTTAAATGCACCATCATCGCAGACGCCAT<br>TACCGGAAATACCTTATATGAGACCGGAGAATGTGCCCGTCGTGTGTCTCCGTGCTCGTCTTTTAAACTTCCATTGGCAAT<br>CATGGGGTTTGATAGTGGAATCTTGCAGTCGCCAAAATCACCTACGTGGGAATTGAAGCCGGAATACAACCCGTCTCCGA<br>GAGATCGCACATACAAACAAGTCTATCCGGCGCTATGGCAAAGCGACTCTGTTGTCTGGTTCTCGCAGCAATTAACAAGC<br>CGTCTGGGAGTTGATCGGTTCACGGAATACGTAAAGAAATTTGAGTACGGTAATCAAGATGTTTCCGGTGACTCGGGGAA<br>GCATAACGGCTTGACCCAGTCATGGCTGATGTCGTCGCTCACCATATCTCCCAAGGAGCAAATTCAGTTTCTTCTACGCTT<br>TGTCGCGCATAAGCTGCCTGTATCCGAAGCGGCTTATGACATGGCGTATGCCACAATCCCGCAGTACCAGGCAGCCGAAG<br>GATGGGCTGTACATGGAAAAAGCGGCAGCGGCTGGCTTCGGGACAATAACGGCAAGATAAATGAAAGTCGTCCGCAGGG<br>CTGGTTCGTGGGCTGGGCTGAAAAAAACGGACGGCAAGTTGTTTTCGCCCGATTGGAAATAGGAAAGGAAAAGTCCGAT<br>ATTCCCGGCGGGTCTAAAGCACGAGAGGATATTCTCGTGGAATTACCCGTGTTGATGGGTAACAAATGATATGTGGCGTC<br>ATCGAGAGCAGATGCATAACCCTGCGCTCGAGCGGACCTCGCGCATAAAGCCGCGCGAGTCCGCTCACCTTGAACG
$\textit{bla}_{\text{OXA-10}}$	TTAGCCACCAAGAAGGTGCCATGAAAACATTTGCCGCATATGTAATTATCGCGTGTCTTTCGAGTACGGCATTAGCTGGTT<br>CAATTACAGAAAATACGTCTTGGAACAAAGAGTTCTCTGCCGAAGCCGTCAATGGTGTCTTCGTGCTTTGTAAAAGTAGC<br>AGTAAATCCTGCGCTACCAATGACTTAGCTCGTGCATCAAAGGAATATCTTCCAGCATCAACATTTAAGATCCCCAACGC<br>AATTATCGGCCTAGAAACTGGTGTCATAAAGAATGAGCATCAGGTTTTCAAATGGGACGGAAAGCCAAGAGCCATGAAG<br>CAATGGGAAAGAGACTTGACCTTAAGAGGGGCAATACAAGTTTCAGCTGTTCCCGTATTTCAACAAATCGCCAGAGAAGT<br>TGGCGAAGTAAGAATGCAGAAATACCTTAAAAAATTTTCCTATGGCAACCAGAATATCAGTGGTGGCATTGACAAATTCT<br>GGTTGGAAGGCCAGCTTAGAATTTCCGCAGTTAATCAAGTGGAGTTTCTAGAGTCTCTATATTTAAATAAATTGTCAGCAT<br>CTAAAGAAAACCAGCTAATAGTAAAAGAGGCTTTGGTAACGGAGGCGGCACCTGAATATCTAGTGCATTCAAAAACTGG

	TTTTTCTGGTGTGGGAACTGAGTCAAATCCTGGTGTCGCATGGTGGGTTGGGTGGGTTGAGAAGGAGACAGAGGTTTACT<br>TTTTCGCCTTTAACATGGATATAGACAACGAAAGTAAGTTGCCGCTAAGAAAATCCATTCCCACCAAAATCATGGAAAGT<br>GAGGGCATCATTGGTGGCTAACAAGTCGCTCAAGGTCGCTCCCTGCGGTCGCTGGACAGTCCCAGTCGGCGCATGCTTCG<br>CATTTTATGCGCCGCCTGTGCCTGCCCCTTAGCTCCAACG
bla$_{\text{OXA-20}}$	TTAGGCACCAAAGGAGGCTCCTTGATAATCCGATTTCTAGCACTGCTTTTCTCAGCTGTTGTACTTGTCTCTCTTGGTCATG<br>CACAAGAAAAAACGCATGAGAGCTCTAATTGGGGGAAATACTTTAGTGATTTCAACGCTAAAGGTACAATAGTTGTAGTA<br>GATGAACGCACAAACGGTAATTCCACATCGGTTTATAATGAATCCCGGGCTCAGCAGCGCTATTCGCCTGCGTCCACATT<br>CAAGATTCCGCATACCCTTTTTGCGCTGGATGCAGGGGCGGTTCGCGATGAGTTTCATGTTTTTCGATGGGACGGCGCTAA<br>AAGAAGCTTTGCAGGTCACAATCAAGACCAAAACCTACGATCGGCAATGCGCAATTCTACCGTTTGGGTCTATCAACTAT<br>TCGCAAAAGAAATAGGCGAAAACAAAGCACGAAGCTACCTAGAAAAATTAAACTACGGCAATGCAGACCCCTCGACCAA<br>GAGCGGTGACTACTGGATAGATGGAAATCTTGCAATTTCAGCAAATGAACAAATTTCCATCCTAAAGAAGCTTTATCGAA<br>ATGAGCTTCCTTTTAGGGTAGAGCACCAACGCTTGGTTAAAGACTTGATGATTGTCGAAGCCAAACGCGATTGGATACTA<br>CGTGCCAAAACAGGCTGGGATGGTCAAATGGGTTGGTGGGTCGGTTGGGTAGAGTGGCCTACAGGCCCAGTATTTTTGC<br>GTTAAATATCGACACGCCAAACAGGATGGAAGACCTTCATAAACGAGAGGCAATTGCGCGTGCTATTCTTCAATCCGTCA<br>ATGCTTTGCCACCCAACTAGCAGCCCAAACCCCCTGTTGTGCCTAACAAGGCGCTCAAGTCGGACAGCCCAAACCGGCGC<br>ATGCTTCGCATTATGCGCGCCGGTTCGGTACGTTGCGCGCTTCGCGCTCCACTCTGCCGCTTAGCTTGGCG
bla$_{\text{OXA-21}}$	TTGGGCGTCAAGGAAAACTTAATGGCAATCCGAATCTTCGCAATACTTTTCTCCACTTTTGTTTTTGGCACGTTCGCGCAT<br>GCACAAGAAGGCATGCGCGAACGTTCTGACTGGCGGAAGTTTTTCAGCGAATTTCAAGCCAAAGGCACGATAGTTGTGGC<br>AGACGAACGCCAAACAGATCGTGTCATATTGGTTTTTGATCAGGTGCGGTCAGAGAAACGCTACTCGCCGGCCTCGACAT<br>TCAAGATTCCACATACACTTTTTGCACTTGACGCAGGCGCTGCACGTGATGAGTTTCAAGTTTTCCGATGGGACGGCATCA<br>AAAGAAGCTTTGCAGCTCACAACCAAGACCAAGACTTGCGATCAGCAATGCGGAATTCTACTGTCTGGATTTATGAGCTA<br>TTTGCAAAAGAGATCGGTGAAGACAAGGCTCGACGCTATTTGAAGCAAATCGACTATGGCAACGCCGATCCTTCGACAAG<br>TAATGGCGATTACTGGATAGATGGCAATCTTGCTATCGCGGCACAAGAACAGATTGCATTTCTCAGGAAGCTCTATCATA<br>ACGAGTTGCCCTTTCGGGTAGAACATCAGCGCTTGGTCAAGGACCTCATGATTGTGGAAGCCGGTCGCAACTGGATACTG<br>CGCGCAAAGACGGGCTGGGAAGGCCGCATGGGTTGGTGGGTAGGATGGGTTGAGTGGCCGACTGGCCCCGTATTCTTCGC<br>ACTGAATATTGATACGCCAAACAGGATGGATGACCTTTTCAAAAGGGAGGCAATAGTGCGGGCAATCCTTCGCTCTATCG<br>AAGCGTTGCCGCCCAACCCGGCAGTCAACTCGGACGCAGCGCGATAAAGCCGCGCAGCGCCGGTTACTTCTACG
bla$_{\text{OXA-46}}$	TTGGGCATCAAAGGAAATTTAATGGCAATCCGATTCTTCACCATACTGCTATCCACCTTCTTTCTTACCTCATTCGTGTATG<br>CGCAAGAACATGTGGTAATCCGTTCGGACTGGAAAAAGTTCTTCAGCGACCTCCAGGCCGAAGGTGCAATCGTTATTGCA<br>GACGAACGTCAAGCGAAGCATACTTTATCGGTTTTTGATCAAGAGCGAGCGGCAAAGCGTTACTCGCCAGCTTCAACCTT<br>CAAGATACCCCACACACTTTTTGCACTTGATGCAGACGCCGTTCGTGATGAGTTCCAGGTTTTTCGATGGGACGGCGTTAA<br>CCGAAGCTTTGCAGGTCACAATCAAGACCAAGATTTGCGATCAGCGATGCGAAATTCTACGGTTTGGGTTTATGAGCTGT<br>TTGCAAAAGATATCGGAGAGGACAAAGCAAGACGTTATTTAAAGCAAATTGATTATGGCAACGTCGATCCTTCGACAATC<br>AAGGGCGATTACTGGATAGATGGAAATCTTAAAATCTCAGCGCACGAACAGATTTTGTTTCTCAGAAAACTCTATCGAAA<br>TCAGTTACCATTTAAGGTGGAGCACCAGCGCTTGGTGAAAGATCTCATGATTACGGAAGCCGGGCGCAGTTGGATACTAC<br>GCGCAAAGACCGGCTGGGAAGGCAGGTTTGGCTGGTGGGTAGGGTGGATTGAATGGCCAACAGGCCCCGTATTCTTTGCG<br>CTGAATATTGATACGCCAAACAGAACGGACGATCTTTTCAAAAGAGAGGCCATCGCACGGGCAATCCTTCGTTCTATTGA<br>CGCATTGCCACCCAACTAACCAATCCAGCCGACGCCTTCGACGCGGCTGATTTCAAACG
bla$_{\text{OXA-53}}$	TTGGGCATCAAGGAAAAATTAATGGCAATCCAAATCTTCGCAATACTTTTCTCCACTTTTGTTCTTGCCACTTTTGCACATG<br>CGCAAGATGGCACGCTGGAACGTTCTGACTGGGGGAAATTTTTCAGCGATTTTCAGGCCAAAGGTACGATAGTTGTGGCA<br>GACGAACGCCAAGCGGATCATGCGATATTGGTTTTTGATCAAGCACGGTCAATGAAACGCTACTCGCCTGCGTCGACATT<br>CAAGATTCCACATACACTTTTTGCACTTGATGCAGGCGCCGTTCGCGATGAGTTTCAGATTTTCCGCTGGGACGGCGTCAA<br>AAGGAGCTTTGCAGGTCACAATAAAGACCAAGATTTGCGATCAGCAATGCGAAATTCTACTGTCTGGGTTTATGAGCTAT<br>TTGCAAAGGAAATCGGTGATGGCAAGGCTCGACGCTATTTGAAGCAAATCGGCTATGGCAACGCCGATCCTTCGACAAGT<br>CATGGCGATTACTGGATAGAAGGCAGCCTTGCAATCTCAGCACAGGAACAGATCGCGTTTCTCAGAAAGCTCTATCAAAA<br>CGATCTGCCCTTTAGGGTGGAACATCAGCGCTTGGTCAAGGATCTGATGATTGTGGAAGCGGGACGCAACTGGATTCTGC<br>GCGCGAAGACGGGCTGGGAAGGCAGCATGGGTTGGTGGGTGGGGTGGGTTGAATGGCCAACCGGTCCCGTATTCTTTGCC<br>TTGAATATCGATACGCCAAACAGAATGGACGATCTTTTCAAGAGGGAAGCAATAGCGCGAGCGATACTTCTCTCTATCGA<br>AGCGTTGCCGCCCAACCCGGCAGTCCACTCGGACGCTGCGCGATGAGGCAGCGCAGCGCCGGTAACTTCTACG
bla$_{\text{OXA-118}}$	TTGGGCGTCAAAGGAAACTTAATGGCAATCCGATTCCTCACCATACTGCTATCTACTTTTTTTCTTACCTCATTCGTGCATG<br>CGCAAGAACACGTGCTAGAGCGTTCTGACTGGAAGAAGTTCTTCAGCGACCTCCGGGCCGAAGGTGCAATCGTTATTTCA<br>GACGAACGTCAAGCGGAGCATGCTTTATTGGTTTTTGGTCAAGAGCGAGCAGCAAAGCGTTACTCGCCTGCTTCAACCTT<br>CAAGCTTCCACACACACTTTTTGCACTCGATGCAGACGCCGTTCGTGATGAGTTCCAGGTTTTTCGATGGGACGGCGTTAA<br>ACGGAGCTTTGCGGGCCATAATCAAGACCAAGACTTGCGATCAGCGATGCGAAATTCTGCGGTCTGGGTTTATGAGCTAT<br>TTGCAAAAGAGATCGGAAAGGACAAAGCAAGACACTATTTAAAGCAAATTGATTATGGCAACGCCGACCCTTCGACAAT<br>CAAGGGCGATTACTGGATAGATGGCAATCTTGAAATCTCAGCGCACGAACAGATTTCGTTTCTCAGAAAACTCTATCGAA<br>ATCAGCTGCCATTTCAGGTGGAACATCAGCGCTTGGTCAAAGATCTCATGATTACGGAAGCCGGGCGCAACTGGATACTA<br>CGCGCAAAGACCGGCTGGGAAGGCAGGTTTGGCTGGTGGGTAGGGTGGGTGGAGTGGCCAACCGGTCCCGTATTCTTCGC<br>GCTGAATATTGATACGCCAAACAGAACGGATGATCTTTTCAAAAGAGAGGCAATCGCGCGGGCAATCCTTCGCTCTATCG<br>ACGCATTGCCGCCCAACTAATCAATCCAGCGGACGCCTTCGGCGCCGCTGATTTCAACG
bla$_{\text{OXA-129}}$	TTAGCCACCAAGGTACCATGAAAACCATAGCCGCATATTTAGTTCTAGTATTTTTTGCAGGCACTGCACTTTCAGAGTCTA<br>TTTCTGAAAATTTAGCTTGGAATAAAGAATTTTCCAGTGAATCAGTGCATGGTGTTTTTGTACTTTGTAAAAGCAGTAGTA<br>ATTCCTGTACAACAAATAATGCAACACGTGCATCTACGGCCTATATTCCAGCATCAACATTCAAAATTCCCAATGCTCTCA<br>TAGGCCTTGAAACCGGCGCCATAAAAGATGCGCGGCAGGTTTTCAAATGGGACGGCAAGCCCAGAGCCATGAAGCAATG<br>GGAAAAAGACTTAACGCTAAGGGGCGCTATACAAGTTTCTGCTGTTCCGGTATTTCAACAAATTGCCAGAGACATTGGCA<br>AAAAAAGAATGCAAAAATACCTTAACCTTTTTTCATATGGCAACGCCAATATAGGCGGAGGCATTGACAAATTTTGGCTA<br>GAAGGTCAGCTTAGAATCTCAGCAGTCAATCAAGTTAAATTTTTAGAGTCGCTTTACCTAAATAATTTGCCAGCATCTAAA<br>GCAAACCAACTTATAGTAAAAGAGGCAATAGTTACAGAAGCAACTCCAGAATATATAGTGCATTCAAAAACCGGGTATT<br>CCGGTGTGGGCACAGAATCAAATCCTGGTGTCGCTTGGTGGGTTGGTTGGGTAGAAAAAGGAACTGAGGTTTACTTTTTT<br>GCATTTAACATGGACATAGACAATGAGAGTAAGTTGCCGTCAAGAAAATCCATTCCAACGAAAATCATGGCAAGTGAAG<br>GTATCATCATTGGTGGCTAACAAGGCGCTCAAGGCCGTGGCTGCGCCACTGGACGTCTCCAATCGGCGCATGCTTCGCAT<br>TTTGCGCGCCGCTTGTAGCCGCCCCTTAGCTTTGCG
bla$_{\text{OXA-198}}$	TTATGCATAAACACATGAGTAAGCTCTTCATCGCTTTTTTAGCCTTTCTGCTGTCGGTGCCAGCAGCCGCTGAAGACCAGA<br>CACTTGCCGAGCTCTTTGCCCAACAAGGCATTGACGGGACTATAGTGATTTCGTCGCTACACAACGGAAAGACATTTATC<br>CACAACGATCCCCGCGCAAAACAGAGATTCTCGACAGCATCCACGTTCAAGATACTGAACACGCTGATCTCGCTCGAAGA<br>AAAAGCCATCTCTGGAAAAGACGATGTGCTGAAATGGGACGGGCATATTTACGATTTTCCAGATTGGAATCGTGACCAGA<br>CGCTAGAAAGTGCGTTCAAGGTTTCCTGTGTCTGGTGTTATCAGGCGCTTGCACGCCAGGTCGGCGCGGAGAAGTATCGA<br>AATTATTTACGCAAGTCAGTTTACGGAGAATTACGCGAGCCTTTTGAGGAAACAACATTCTGGCTTGATGGTTCACTTCAA<br>ATCAGCGCAATTGAACAAGTGAATTTCCTCAAGAAAGTTCATCTGCGCACTCTCCCATTCAGTGCATCGTCCTACGAAACG<br>CTACGACAAATCATGCTTATCGAGCAAACGCCGGCTTTTACGCTGCGGGCCAAGACAGGCTGGGCAACAAGAGTAAAGC<br>CGCAAGTTGGCTGGTATGTGGGCCATGTCGAAACTCCAACGGATGTATGGTTCTTTGCCACGAATATTGAAGTCCGTGAC<br>GAAAAAGACTTGCCCTTACGTCAGAAGCTAACGCGAAAAGCATTACAAGCAAAGGGGATCATCGAATAATGCATAACAT<br>GGCGCTCAAGCGGGACGCGGCAAAATGCCGCCGCGCCCCTTAGCTCTACG
bla$_{\text{PBL-1}}$	TTAGGCCGCACAATCAGCACATCATGTGACCTTATGTTTTCTTGAAACAAACATATGCACGTCATATCGCGGTGTTCATAG<br>ACAATCATTAAAGGAAGGAGATAGGAATGGTTACAAGGCGAGATTTTTTGATTGCTTCAGCGGCAACGGCCGCTCTTCTA<br>ACTTTGCCAGCTACTGCAAAAGTTCCCTCGCGTCGCTTTGACGCATCAGCGAAGCTTCAGTCGCTTGAAGCGGGACAGGC

	GCGCCTTGGTGTTTGCTTCCTCGATACGGTTACTGGTGAAGTCAGCGGTAACCGTATCGAGGAGCGCTTTGCAATGTGCTC<br>GACGGTCAAGCTGGCAATAGTTGCCGCCTGCTTGCGCGAGGCAGATAAGGCGCGCTTGAATCTCGAGGAGATACTGACCT<br>ATTCAGAGGCGGATCTTCTTCCTTGGGCGCCAGTGACGCGCAAGAATCTCGCCAAAGGCGGTCTGAGTATTTCCGCTCTG<br>GCGCAAGCGGCTCAAGAAATGAGCGATGGTGTTGCCGCTAACCTTTTAATCAAACGCCTAGGCGGTCCTGCCGCCGTCAC<br>CGCGAAGTTCCGAGAAATGGGCGATTCGGTGACCCGCCTTGATCGTTATGAGCCAGACTTAGGGTTAGTTCTTTCAGCCG<br>ATGTTCGCGACACCACCACGCCCCTTGCTTATGCGCAACTTGTCCGTCGAATCACGACTGGCCGTGTTTTGTCGCACGGAT<br>CGCGGGAACAACTTTTAGAGTGGATGCGAAATACAGTCACAGGCGCCAGTCGGCTGCGAGCCGGCCTCCCCACTGAATG<br>GCGTACTGGGAACAAGACAGGGACTGGGCGCGACGAAGGGACGACCAACAAGTGCAATGACGTCGCTATTACTTTTCCG<br>CCAAGCAAGAACCCAATCATCATTGCAGCCTATTTCGACAGCGGCGAATACACAGAAAAGGTAGAGGCGAGGCATGAGG<br>CTGTCCTCGCTGAAGTGGGAAAGATTGCTGCCGAATGGGGCGAGAGTTAGTGTGCGAACTACACAATCACTTTATTTAAA<br>AAATTAGAATCTAGGTAGGTACTTACGTCTTTTGAAGAATTTCTAATTTTTGTCATGAAAAAGGGATTAAAAAAAAGAAG<br>CCTAACAAATCGCTCAAGCACCGCACACTTCGTGTGCGGGACAGTTTTTAAGTCGCAGTTTTGTGGTTTTGCTGCGCAAAA<br>TTATTCCACAAAACCGCAACTTAAAAACTGCCGCTTAGCTCGGCG
bla$_{\text{SIM-1}}$	TTAGAGGTAGTTAAAAATGAGAACTTTATTGATTTTATGTTTATTCGGCACTTTAAATACCGCGTTTGCGGAAGAAGCCCA<br>GCCAGATTTAAAAATTGAAAAAATAGAAGAAGGGATCTATCTTCATACATCTTTTCAAGAGTACAAGGGATTCGGCATCG<br>TTAAAAAACAAGGCTTAGTAGTTCTTGACAATCACAAGGCATATCTCATCGACACTCCAGCTTCCGCAGGAGATACTGAA<br>AAGCTAGTAAACTGGCTCGAAAAAAATGATTTCACTGTCAATGGAAGCATTTCAACACATTTCCACGACGACAGTACTGC<br>TGGGATAGAGTGGCTTAATACAAAGTCCATCCCCACATATGCATCTAAATTGACAAATGAATTGCTAAATAAAAATGGCA<br>AAACTCAAGCCAAGCACTCTTTTGATAAAGAGAGCTTTTGGTTGGTCAAAAATAAAATTGAAATTTTTTATCCAGGCCCA<br>GGACACACTCAAGATAACGAAGTTGTCTGGATACCTAATAAAAAAATCCTATTCGGGGGCTGTTTTATAAAACCGAATGG<br>CCTTGGCAATCTAAGTGACGCAAATTTGGAAGCTTGGCCAGGCTCCGCAAAAAAAATGATATCAAAATACAGTAAGGCA<br>AAACTTGTTATCCCAAGCCACAGTGAAATCGGAGACGCATCACTATTGAAACTCACATGGGAACAGGCCATTAAAGGTTT<br>AAATGAAAGCAAATCAAAACCGCCGCTCATTAATTAACCCCCCCAAGGTCATTACGAAAACTCTGAGTGCCTCTAACAAT<br>GCGCTAAACACCGCTCGCTTCGCTCACTGGACTCGCAAAAGCTGCGCTTTTGCTCGCCTGTTAGCTTAATCG
bla$_{\text{TMB-1}}$	TTAGAGGAATAATGGAATGCGACCATTTTTATTTTTAATAATTTTTATCAGTCATTTCGCTTTTGCCAACGAAGAAATACC<br>CGGATTGGAAGTTGAGGAAATTGACAACGGCGTTTTTTTGCACAAGTCATACAGCCGGGTGGAAGGCTGGGGCCTGGTAA<br>GTTCAAACGGACTTGTTGTCATCAGCGGCGGAAAAGCATTCATTATTGACACTCCATGGTCGGAATCAGATACAGAAAAG<br>CTTGTAGATTGGATACGATCAAAAAAGTATGAGCTGGCGGGAAGCATTTCTACACATTCACACGAAGACAAGACTGCCGG<br>TATAAAATGGCTAAACGGCAAATCCATTACTACATATGCCTCAGCGCTGACTAATGAAATTCTAAAAAGAGAGGGTAAGG<br>AGCAGGCAAGGAGCTCATTCAAAGGTAATGAATTTTCGCTGATGGACGGTTTTCTAGAAGTCTATTATCCCGGAGGCGGC<br>CATACTATTGATAACTTAGTGGTATGGATCCCTAGTTCAAAAATATTGTATGGCGGCTGTTTCATACGTAGCTTGGAATCC<br>AGTGGGCTAGGTTACACTGGTGAAGCTAAAATTGATCAGTGGCCACAATCCGCTAGAAATACAATTTCGAAGTATCCTGA<br>AGCTAAGATTGTGGTGCCTGGTCATGGAAAAATTGGCGATTTCGAGTTGTTAAAACATACCAAGGTCCTTGCAGAAAAGG<br>CCTCTAACAAGGCCAATCACGGCGACCGCTGACGCGGCGCGTGTTGGCATAG
bla$_{\text{VEB-1}}$	TTAGCGGTAATTTAACCAGATAGGAGTACAGACATATGAAAATCGTAAAAAGGATATTATTAGTATTGTTAAGTTTATTTT<br>TTACAATTGTGTATTCAAATGCTCAAACTGACAACTTAACTTTGAAAATTGAGAATGTTTTAAAGGCAAAAAATGCCAGA<br>ATAGGAGTAGCAATATTCAACAGCAATGAGAAGGATACTTTGAAGATTAATAACGACTTCCATTTCCCGATGCAAAGCGT<br>TATGAAATTTCCGATTGCTTTAGCCGTTTTGTCTGAGATAGATAAAGGGAATCTTTCTTTTGAACAAAAAATAGAGATTAC<br>CCCTCAAGACCTTTTGCCTAAAACGTGGAGTCCGATTAAAGAGGAATTCCCTAATGGAACAACTTTGACGATTGAACAAA<br>TACTAAATTATACAGTATCAGAGAGCGACAATATTGGTTGTGATATTTTGCTAAAATTAATCGGAGGAACTGATTCTGTTC<br>AAAAATTCTTGAATGCTAATCATTTCACTGATATTTCAATCAAAGCAAACGAAGAACAAATGCACAAGGATTGGAATACC<br>CAATATCAAAATTGGGCAACCCCAACAGCGATGAACAAACTGTTAATAGATACTTATAATAATAAGAACCAATTACTTTC<br>TAAAAAAAGTTATGATTTTATTTGGAAAATTATGAGAGAAACAACAACAGGAAGTAACCGATTAAAAGGACAATTACCA<br>AAGAATACAATTGTTGCTCATAAAACAGGGACTTCCGGAATAAATAATGGAATTGCAGCAGCCACTAATGATGTTGGGGT<br>AATTACTTTACCGAATGGACAATTAATTTTTATAAGCGTATTTGTTGCAGAGTCCAAAGAAACTTCGGAAATTAATGAAA<br>AGATTATTTCAGACATTGCAAAAATAACGTGGAATTACTATTTGAATAAATAAAAAACTACCGCTAACACTGGCTCATAG<br>GCAATGGCGGGTTGAAGTGCAATTTGCAAAGTCGGTAGCCCGCCCGAGCGTTTTCTCGGTTTGACAGGAAAGGCTCACGC<br>AAACCGCCACTGCCCATAGCCCAAACCG
bla$_{\text{VIM-1}}$	TTATGCCGCACCCACCCCTATGGAGTCTTGATGTTAAAAGTTATTAGTAGTTTATTGGTCTACATGACCGCGTCTGTCATG<br>GCTGTCGCAAGTCCGTTAGCCCATTCCGGGGAGCCGAGTGGTGAGTATCCGACAGTCAACGAAATTCCGGTCGGAGAGGT<br>CCGACTTTACCAGATTGCCGATGGTGTTTGGTCGCATATCGCAACGCAGTCGTTTGATGGCGCGGTCTACCCGTCCAATGG<br>TCTCATTGTCCGTGATGGTGATGAGTTGCTTTTGATTGATACAGCGTGGGGTGCGAAAAACACAGCGGCACTTCTCGCGG<br>AGATTGAAAAGCAAATTGGACTTCCCGTAACGCGTGCAGTCTCCACGCACTTTCATGACGACCGCGTCGGCGGCGTTGAT<br>GTCCTTCGGGCGGCTGGGGTGGCAACGTACGCATCACCGTCGACACGCCGGCTAGCCGAGGCAGAGGGGAACGAGATTC<br>CCACGCATTCTCTAGAAGGACTCTCATCGAGCGGGGACGCAGTGCGCTTCGGTCCAGTAGAGCTCTTCTATCCTGGTGCTG<br>CGCATTCGACCGACAATCTGGTTGTATACGTCCCGTCAGCGAACGTGCTATACGGTGGTTGTGCCGTTCATGAGTTGTCAA<br>GCACGTCTGCGGGGAACGTGGCCGATGCCGATCTGGCTGAATGGCCCACCTCCGTTGAGCGGATTCAAAAACACTACCCG<br>GAAGCAGAGGTCGTCATTCCCGGGCACGGTCTACCGGGCGGTCTAGACTTGCTCCAGCACACAGCGAACGTTGTCAAAGC<br>ACACAAAAATCGCTCAGTCGCCGAGTAGCAGATGCGGCATAACAAATCGTTGGAGCGGGACTTTTGCTACGCAGGCTGCG<br>CCTACTCCGCAAAAGCCCCTCAACTCAGGCG
bla$_{\text{VIM-2}}$	TTATGCCGCACTCACCCCCATGGAGTTTTGATGTTCAAACTTTTGAGTAAGTTATTGGTCTATTTGACCGCGTCTATCATGG<br>CTATTGCGAGTCCGCTCGCTTTTTCCGTAGATTCTAGCGGTGAGTATCCGACAGTCAGCGAAATTCCGGTCGGGGAGGTCC<br>GGCTTTACCAGATTGCCGATGGTGTTTGGTCGCATATCGCAACGCAGTCGTTTGATGGCGCAGTCTACCCGTCCAATGGTC<br>TCATTGTCCGTGATGGTGATGAGTTGCTTTTGATTGATACAGCGTGGGGTGCGAAAAACACAGCGGCACTTCTCGCGGAG<br>ATTGAGAAGCAAATTGGACTTCCTGTAACGCGTGCAGTCTCCACGCACTTTCATGACGACCGCGTCGGCGGCGTTGATGT<br>CCTTCGGGCGGCTGGGGTGGCAACGTACGCATCACCGTCGACACGCCGGCTAGCCGAGGTAGAGGGGAACGAGATTCCC<br>ACGCACTCTCTAGAAGGACTCTCATCGAGCGGGGACGCAGTGCGCTTCGGTCCAGTAGAACTCTTCTATCCTGGTGCTGC<br>GCATTCGACCGACAACTTAGTTGTGTACGTCCCGTCTGCGAGTGTGCTCTATGGTGGTTGTGCGATTTATGAGTTGTCACG<br>CACGTCTGCGGGGAACGTGGCCGATGCCGATCTGGCTGAATGGCCCACCTCCATTGAGCGGATTCAACAACACTACCCGG<br>AAGCACAGTTCGTCATTCCGGGGCACGGCCTGCCGGGCGGTCTAGACTTGCTCAAGCACACAACGAATGTTGTAAAAGCG<br>CACACAAATCGCTCAGTCGTTGAGTAGCAGGCAGATGCGGCATAACATGAAGTTGCAGCCGACCATCACTCCGCTGCGCT<br>CCGTTCTGGCGGCTGAACTTCGGCG
bla$_{\text{VIM-7}}$	TTATCGCAGTCGGCCCCCGAGGAGTATTGATGTTTCAAATTCGCAGCTTTCTGGTTGGTATCAGTGCATTCGTCATGGCCG<br>TACTTGGATCAGCAGCATATTCCGCACAGCCTGGCGGTGAATATCCGACAGTAGATGACATACCGGTAGGGGAAGTTCGG<br>CTGTACAAGATTGGCGATGGCGTTTGGTCGCATATCGCAACTCAGAAACTCGGTGACACGGTGTACTCGTCTAATGGACT<br>TATCGTCCGCGATGCTGATGAGTTGCTTCTTATTGATACAGCGTGGGGGGCGAAGAACACGGTAGCCCTTCTCGCGGAGA<br>TTGAAAAGCAAATTGGACTTCCAGTAACGCGCTCAATTTCTACGCACTTCCATGACGATCGAGTCGGTGGAGTTGATGTCC<br>TCCGGGCGGCTGGAGTGGCAACGTACACCTCACCCTTGACACGCCAGCTGGCCGAAGCGGCGGGAAACGAGGTGCCTGC<br>GCACTCTCTAAAAGCGCTCTCCTCTAGTGGAGATGTGGTGCGCTTCGGTCCCGTAGAGGTTTTCTATCCTGGTGCTGCGCA<br>TTCGGGCGACAATCTTGTGGTATACGTGCCGGCCGTGCGCGTACTGTTTGGTGGCTGTGCAGTTCATGAGGCGTCACGCGA<br>ATCCGCGGGTAATGTTGCCGATGCCAATTTGGCAGAATGGCCTGCTACCATTAAACGAATTCAACAGCGGTATCCGGAAG<br>CAGAGGTCGTCATCCCCGGCCACGGTCTACCGGGCGGTCTGGAATTGCTCCAACACACAACTAACGTTGTCAAAACGCAC<br>AAAGTACGCCCGGTGGCCGAGTAACAAATGCGGCATAACAACGCCAAGGAGGGGGACGCATTTTCCACTACGCCCTTCG<br>GGCTTCCCTCCAAAAGCGCCCCTCATGGCGGGCG
bla$_{\text{VIM-13}}$	TTATGCCGCACCCACCCCTATGGAGTTTTGATGTTAAAAGTTATTAGTAGTTTATTGTTCTACATGACCGCCTCTCTAATGG<br>CTGTAGCTAGTCCGTTAGCCCATTCCGGGGAGTCGAGAGGTGAGTATCCGACAGTCAGCGAAATTCCGGTCGGAGAAGTT

	CGGCTGTACCAGATTGACGATGGTGTTTGGTCGCATATCGCAACGCATACGTTTGATGGCGTGGTGTACCCGTCCAATGGT<br>CTCATTGTCCGTGATGGCGATGAGTTGCTTTTGATTGATACAGCTTGGGGTACGAAAAACACAGTGGCCCTTCTCGCGGAG<br>ATTGAGAAGCAAATTGGACTTCCCGTAACGCGTTCAGTCTCCACGCACTTTCATGACGACCGCGTCGGCGGAGTTGATGC<br>CCTTAGGGCGGCTGGAGTGGCGACGTACGCATCGCCCTCGACACGCCGTCTAGCCGAGGCAGAGGGGAACGAGGTTCCC<br>ACACACTCTCTAGAAGGGCTCTCATCGAGTGGGGACGCAGTGCGTTTCGGTCCAGTAGAGCTCTTCTATCCTGGTGCTGCG<br>CATTCGACCGACAATCTGGTTGTATACGTCCCGTCAGCGAACGTGCTATACGGTGGTTGTGCCGTTCTGGAATTGTCACGC<br>ACATCCGCGGGAAACGTGGCCGATGCCGACCTGGCTGAATGGCCCGGTTCCGTTGAGCGGATTCAACAACATTACCCAGA<br>AGCAGAGGTGGTCATTCCCGGGCACGGTCTACCGGGCGGTCTAGACTTGCTCCAGCACACAGCGAACGTTGTCAAAGCAC<br>ACACAAATCGCTCAGTCGCCGAGTAGCAGATGCGGCATAACAAATCGCTGCAGTTGACCGCCCAACCCGCTGTCGCGGCT<br>TGGGTTCCCTCCGCGCTTCGCGCTCCGGCGGCAACTGAGCTCAGGCG
aacA1<br>*AAC(6′)-Ia*	TTAGGGCGACGCCGCTATTGCGGCGCGAATACAAAGAGGAAGAGATGAATTATCAAATTGTGAATATTGCGGAATGCAG<br>CAATTATCAGTTAGAAGCAGCAAATATACTAACAGAAGCGTTCAATGATCTTGGTAACAATTCATGGCCAGATATGACGA<br>GTGCAACAAAAGAAGTAAAAGAATGTATTGAGAGTCCAAACCTTTGTTTCGGTCTGCTAATAAATAACTCCTTAGTTGGC<br>TGGATAGGCTTAAGGCCAATGTACAAGGAAACCTGGGAATTGCATCCATTGGTTGTCAGACCAGATTATCAAAATAAAGG<br>TATTGGCAAGATCCTGCTTAAGGAATTAGAAAACAGAGCTAGAGAGCAAGGTATTATTGGAATCGCTTTAGGAACAGATG<br>ATGAATACTATAGAACAAGTCTCTCTTTAATAACTATAACAGAAGATAATATATTTGATTCAATAAAAAATATTAAAAAT<br>ATTAATAAACATCCATATGAGTTTTATCAGAAGAATGGTTATTATATTGTTGGAATAATTCCAAATGCCAATGGTAAAAAC<br>AAACCAGATATTTGGATGTGGAAAAGTTTAATCAAAGAGTAAAACAATGGAACAGATTAATAACATAGAACTTGTAGAT<br>CCATCTATCTACCCAACAGATGAAATCTTAAAAAGAATACTTGGAAAATCGTTTAGTGTATATTTAAAATTACTACGACTC<br>TATGAAAATTATAGTTTAATACCAGAGTGGAAGTATTATAAAGATGGAAAAGCTTGGTTATGTAAAGTTATAAAGGGAAA<br>GAAAACGATTGTTTGGATGTCTGCATGGAAGAACTACATAAAAGCTACTATATATCTTCCAGAAAAACATATTAACGGTG<br>TATTAGTATTAGATATTCATGAGATTACAAAGAAAGCATTTATAGAGACAAATAATATTGGAAGATCAAGGCCTTGCATG<br>TTTGAATTAAAAGAAGAAAATATATTAGAAGATTTCATAAAGGTAATGCAGTTCAAAATGACATTAAAATGATCAACCGA<br>AATTTGCCCTAACAAATGCTTCAACCTGACAATTCCTGTTGTCATGGTTTGTGCTGTCGCTTTGCTCAGCACAAACCACGC<br>CAAGCCCTTCGGGCCGGAATTGCAGGTTAAGCAAATG
aacA2<br>*AAC(6′)-Id*	TTAGGCGTCATGATCGAAGCGTGTCACTCCGTCGAATGCCCTGGCTGGCTGCAACTTCGTTTTCTCCTCTGGCCGCAAGAT<br>AGCGCCGACGAACACCTTGCTGAAATGGCAATCTTCGTGGCTGAGCCAAATCGGTTCGCGCAGTTCATCGCTTACGACGA<br>AGCGAACAAACCACTAGGCTTCGTTGAGGCTGCGCTCCGATCTGACTACGTAAACGGAACCAATTCGTCCCCAGTAGCGT<br>TTCTTGAAGGGGTCTATGTCTTACCGGAAGCACGGCGTCGCGGCATCGCTCATGCCTTGGTCGGTGCAGTCGAGATATGG<br>GCTCGTAATCGGGCCTGTACCGAGTTCGCATCCGATGCATCAACTGACAACCCGGAAAGCCATAGATTTCACCAGTCACT<br>TGGGTTCAAAGAAACCGAGCGTGTCGTGTACTTCAGGAAGATGCTTGCGCCAGAATGACGCCTAACCCTTCCATCGAGGG<br>GATGCCCAAAAGGCTGCGCCTTCTGTGCACCCCTCATGTCAAACG
aacA3<br>*AAC(6′)-IIa*	TTAGGCAGCACAGAGCGACCATTTCATGTCCGCGAGCACCCCCCCCATAACTCTTCGCCTCATGACCGAGCGCGACCTGC<br>CGATGCTCCATGATTGGCTCAACCGGCCGCACATCGTTGAGTGGTGGGGTGGTGACGAAGAGCGACCGACTCTTGATGAA<br>GTGCTGGAACACTACCTGCCCAGAGCGATGGCGGAAGAGTCCGTAACACCGTACATCGCAATGCTGGGCGAGGAACCGA<br>TCGGCTATGCTCAGTCGTACGTCGCGCTCGGAAGCGGTGATGGCTGGTGGGAAGATGAAACTGATCCAGGAGTGCGAGG<br>AATAGACCAGTCTCTGGCTGACCCGACACAGTTGAACAAAGGCCTAGGAACAAGGCTTGTCCGCGCTCTCGTTGAACTAC<br>TGTTCTCGGACCCCACCGTGACGAAGATTCAGACCGACCCGACTCCGAACAACCATCGAGCCATACGCTGCTATGAGAAG<br>GCAGGATTCGTGCGGGAGAAGATCATCACCACGCCTGACGGGCCGGCGGTTTACATGGTTCAAACACGACAAGCCTTCG<br>AGAGAAAGCGCGGTGTTGCCTAACAACTCATTCAAGCCGACGCCGCTTCGCGGCGCGGCTTAATTCAGGTG
aacA4<br>*AAC(6′)-Ib*	TTAGGCATCACAAAGTACAGCATCGTGACCAACAGCAACGATTCCGTCACACTGCGCCTCATGACTGAGCATGACCTTGC<br>GATGCTCTATGAGTGGCTAAATCGATCTCATATCGTCGAGTGGTGGGGCGGAGAAGAAGCACGCCCGACACTTGCTGACG<br>TACAGGAACAGTACTTGCCAAGCGTTTTAGCGCAAGAGTCCGTCACTCCATACATTGCAATGCTGAATGGAGAGCCGATT<br>GGGTATGCCCAGTCGTACGTTGCTCTTGGAAGCGGGGACGGATGGTGGGAAGAAGAAACCGATCCAGGAGTACGCGGAA<br>TAGACCAGTTACTGGCGAATGCATCACAACTGGGCAAAGGCTTGGGAACCAAGCTGGTTCGAGCTCTGGTTGAGTTGCTG<br>TTCAATGATCCCGAGGTCACCAAGATCCAAACGGACCCGTCGCCGAGCAACTTGCGAGCGATCCGATGCTACGAGAAAG<br>CGGGGTTTGAGAGGCAAGGTACCGTAACCACCCCAGATGGTCCAGCCGTGTACATGGTTCAAACACGCCAGGCATTCGA<br>GCGAACACGCAGTGATGCCTAACCCTTCCATCGAGGGGGACGTCCAAGGGCTGGCGCCCTTGGCCGCCCCTCATGTCAAA<br>CG
aacA5<br>*AAC(6′)-IIb*	TTAGGCAGCACGGAGACACTTCAGCATGCATCCCGGCGTTGTTACTCTGCGTCCGATGACCGAAGACGACATCGGTATGC<br>TTCACGAATGGTTGAATCGGCCGCACATTGTCGAATGGTGGGGTGGTGAGCGGCCCTCGCTCGAAGAGGTGAAAGAGGA<br>CTATCGGCCCAGCGCGTTGGCCGAAGAAGGAGTGACGCCGTACATCGGTTTGCTTGACGGAACTCCATTCGCGTTCGCAC<br>AGTCGTACGTTGCGCTCGGGTCGGGTGGTGGATGGTGGGAGGAAGAGACCGATCCTGGTGTCCGCGGAATCGATCAATCA<br>ATCGCCGATTCCGGGCTTCTCGGAAGAGGTTACGGCACTCGGCTGGTGCAGGCGCTTGTTGATTTGCTGTTCGCCGACCCG<br>CAGGTATCCAAGGTTCAGACGGACCCCTCCCCGAACAACATGCGCGCGATACGCTGCTATGAGAAGGCAGGCTTCCGGA<br>AGGTCAAGGTCGTTTCAACACCGGATGGGCCGGCCATGTACATGTTGCACGAGCGTCCGTTGGTGAACGGTTTGCGCAGT<br>GCGGCCTAACTTTTCGCTCCAACGGACGCTTGACCCGTCGGCCCGGCCGCTGCCGCAGCCGGACCGCCGCGTCAACCGCC<br>GCTGACCTCCGGAG
aacA7<br>*AAC(6′)-Il*	TTAGGCACCAATGGATAGTTCGCCGCTCGTCAGGCCTGTTGAAACTACCGATTCGGCCAGTTGGCTAAGCATGCGCTGTG<br>AGCTGTGGCCAGATGGCACATGTCAAGAGCACCAGTCAGAGATCGCAGAATTTCTGTCCGGAAAAGTCGCCCGGCCTGCT<br>GCTGTCCTCATTGCTGTAGCACCCGACGGAGAAGCACTAGGGTTTGCCGAGCTTTCGATCCGCCCGTATGCGGAGGAGTG<br>CTACTCCGGCAACGTTGCGTTCTTGGAGGGTTGGTACGTTGTGCCAAGTGCGCGGCGTCAGGGCGTAGGTGTAGCTCTGG<br>TAAAAGCCGCCGAGCATTGGGCTCGTGGTCGCGGATGCACCGAATTCGCCTCCGACACTCAACTTACCAACAGCGCAAGC<br>ACCTCGGCGCACCTGGCGGCTGGATTCACGGAGGTTGCTCAAGTACGCTGCTTCCGGAAACCGTTGTGAGGGGCGCCGCG<br>TTGGTGCCTAACAATTCGTTCAAGCCGAACTTGCTTCGTTACACCAAAGCCATGGCAGAATGAGCTTGCCATGGCTTTGGC<br>TCCACTACGCAAGTCGGCTTAACTCAGGCG
aacA8	TTAGGCAGCACAAACTCCGTCCTCATGACATCCAGCATTAGTTTCGTCAAGCTGCGCCTCATGACCGAGCAAGACCTTCC<br>GATGCTCCATGAGTGGCTAAACCGGCCTCACATCGTTGAGTGGTGGGGCGGAGAAGAAGCACGTCCAACACTTGCTGAA<br>GTGCAGGAGCAATACCTGCCAAGCGCCTTGGCGAGAGAGTCCGTCACTCCCTACATCGCAATGCTGGATGAAGAACCGAT<br>TGGGTACGCTCAGTCGTACGTTGCACTCGGAAGCGGTGGCGGATGGTGGGAAGACGAAACGGATCCAGGAGTACGCGGA<br>ATTGACCAGTCCCTGGCAAATCCATCGCAGCTGGGAAAGGGGCTAGGAACCAAGCTTGTTCGCGCGCTCGTTGAGATGCT<br>GTTCAAAGACGCTAAGGTAACCAATATCCAAACGGACCCGTCGCCGAACAACTTGCGCGCAATCCGGTGCTACGAGAAG<br>GCGGGGTTTGTGACGCAAAGAACCATAACCACCCCAGACGGGCCGGCTGTGTACATGGTTCAAACACGTCAGGCGTTCG<br>AGCAGGCGCGCAGTGCTGCCTAACCCTTCCATCGAGAGGGACGTCCAAGAGCTATCGCTCTTGGCCGCCCCTCATGTCAA<br>ACG
aacA16<br>*AAC(6′)-Ip*	TTGGGCTGATTGATTTGTTTGTTCTAGCATTACCTATCTGGAGTTTGTTTTGAACTATTCAATATGCGATATAGCTGAATCA<br>AATGAATTAATCCTTGAAGCAGCAAAGATGCTTAAGAAAAGCTTTCTTGATGCTGGAAATGAATCATGGGGAGATATTAA<br>AAATGCTATTGAAGAAGTTGAAGAATGTATAGAACATCCAAATATATGCTTGGGAATATGTCTGGATGATAAACTGATTG<br>GATGGACCGGATTAAGGCCGATGTACGATAAGACCTGGGAACTTCATCCCATGGTTATAAAAACTGAATATCAAGGCAA<br>GGGTTTTGGGAAAGTACTACTAAGAGAACTAGAGACGAGAGCGAAGAGTAGGGGAATTATCGGAATAGCTCTTGGAACT<br>GATGACGAATATCAGAAAACTAGTTTGTCTATGATTGATATAAACGAACGAAATATCTTCGATGAAATCGGGAATATAAA<br>GAACGTTAATAATCATCCATATGAGTTTTATAAGAAATGTGGTTATATGATCGTTGGAATAATCCCTAATGCTAATGGAAA<br>AAGAAAACCAGATATATGGATGTGGCAGATATTAGCTAGGAAGAACAGCCCAACAATCGCTTCAACCTGACTCAGGGCG<br>CCGTCACGATTTCTGCTAGTTATCCTGGGACGCAGAAATCGCGCCAACGCCCTTCGCAGGTTAAGCGAAGTG
aacA17	TTGGGCTGATTGATTTGTTTGTTCTAGTATTACCTATCTGGAGTTTGTTTTGGACTATTCAATATGCGATATAGCGGAATCA<br>AATGAATTAATCCTTGAAGCAGCAAAAATACTTAGGAAAAGCTTTCTTGATGCTGGAAATGAATCATGGGTAGATATCAA

AAC(6′)-Iq	AAAGGCTATTGAAGAAGTTGAGGATTGTATAGAACACCCAAATCTATGCTTGGGAATATGTCTGGATGATAAACTGATTG<br>GCTGGACCGGATTAAGGCCGATGTACGATAAGACCTGGGAACTTCATCCCATGGTTATAAAAACTGAGTATCAATGCAGG<br>GGTATTGGGAAAGTCTTAATAAAAGAACTAGAGAAGAGAGCGAAGGGTAGGGGAATTATCGGAATAGCTCTTGGAACTG<br>ATGATGAATATCAGAAAACTAGTTTGTCTATGATTGATATAAACGAACGAAACATCTTCGATGAAATCGGGAACATAAAG<br>AACGTTACTAATCATCCATATGAGTTTTATAAGAAATGTGGTTATATGATCGTTGGAATAATCCCTAATGCTAATGGAAAA<br>AGAAAACCAGATATTTGGATGTGGAAAGATATTAGCTAGGAAGAACAGCCCAACAACCGCTTCAACCTGACTCAGGGCG<br>CTGTCACGATTTCTGCAGGTAATTCTGGACGCAGAAATCGCGCCAACGCCCTTCGCAGGTTAAGCGAAGTG
aacA27<br>*AAC(6′)-IIc*	TTAGGCCCGCACGGAATCAACATCTCATGTCCGCCAACAATGCCGCAATAGTTCTACGAGTCATGGCCGAGAACGATCTG<br>CCAATGCTCCATGCTTGGCTGAACCGCCCCCACATAGTCGAGTGGTGGGGCGGCGAGGATGAACGCCCAACTCTTGACGA<br>AGTCTTAGAACACTATTCGCCCGAAGTTCTGGCAAAGCAAGCTGTAGTGCCTTACATCGCAATGCTAGATGACGAACCCA<br>TCGGCTACGCCCAATCCTACATCGCACTTGGAAGTGGCGATGGATGGTGGGAAGACGAAACTGATCCAGGGGTCCGCGG<br>GATTGACCAGTCTTTGGCTAATCCATCACAGTTAAACAAGGGGTTGGGTACAAAGCTCGTACGCTCGCTCGTTGAACTCCT<br>GTTTAGCGACCCGGCCGTAACGAAAATCCAAACCGATCCATCTCCTAGCAACCATCGCGCCATTCGCTGCTACGAGAAGG<br>CCGGGTTCGTTCAAGAAAAAAACATCCTCACACCTGACGGCCCTGCGGTGTACATGGTCCAAACACGCCAGGCGTTCGAA<br>AGCCTGCGCACTGTTCAAAGCTTCAAAATCAAGGGGAAGTGGTCATGAATGGCTGCACCATCTCTACCAACTGGTATCTC<br>GGCGTGTTCTCATCTGCCTTCGCATCTGTCGTCAGCCTAACCGGGCGTTCAACCGGACATCAACGTGCTGCGCACGTTGAT<br>GTCGGTTAACTGGGGCG
aacA28<br>*AAC(6')-Iae*	TTAGCCGGACGCTGCGCGCGAAGAGGTTTTATGAAATACAACATTGTTAATATTAAAGATTCTGAAAAGTATATAACGCA<br>AGCTGCAGAAATTCTATTTGATGTATTTTCACACATAAATTTCGATTCTTGGCCGTCACTCCAAAAGGCTACAGAAACTGT<br>AATAGAATGTATTAGCGCCGAAAACATTTGTATTGGCATTTTAATAAACGATGAATTGTGTGGTTGGGTTGGATTAAGAG<br>AAATGTATAAAAAAACTTGGGAACTACATCCAATGGTTATTAAGAAAAAACATCAAAATAAGGGATTTGGTAAAATACT<br>AATTTTTGAAACAGAAAAGAAAGCGAAAGAAAGAAATTTAGAAGGAATTGTACTTGGAACAGACGATGAAACATTTAGA<br>ACTACATTATCAATGTCAGAATTAAATAATGAAAATATATTCCATGAAATTAAAAATATAAAAAATCTAAAAAATCATCC<br>ATTTGAATTTTATGAAAAATGTGGTTACAGTATTATTGGTGTGATTCCTAATGCAAATGGGAAAAATAAACCTGATATATT<br>AATGTGGAAAAATATAATGTAAAAAAATTTCGGCTAACAACTGCTTCAACTTGATAATTGCTTGAGCAATTACAAGTTAA<br>GCAAATG
aacA29<br>*AAC(6′)-29*	TTAGACGGCTTTGAGCGTTTCGATCTTACCTGTGAAAGAACAAGACGCTGCCGACTGGCTAGCGCTGCGGAATCTTCTTTG<br>GCTCGCGGATGATCACGCCTCGGAGATTGAGCAGTACTTCTCTGGTGGACTTGAGGAGCCTGTAGAAGTGCTCATCGCAC<br>GTGATGCTACCGGCGCGGCTGTTGGGCATGTCGAACTCTCGATAAGACATGACTTGGAAGAACTCCAAGGAATCAAGACC<br>GGCTACATCGAAGGCCTTTATGTGGCCCCAAGCCATCGATCAACAGACCTTGTGAAGCGTTTCTTGCGTGAGTCCGAGAA<br>GTGGGCCCTAGAACAAGGGTGCAGCGCATTTGCCTCAGACAGAAGTGATCGGGTCATCACGCACCGCAAGTTCGCAGGC<br>AGCGCCGTCTAACAACTCGTTCAAGCCGAACCCGCTTCGCTCCGGCAACGGCGTGGCAGGTTAAGCTTGCCACGCCGCCG<br>CCTCCACTATGCGGGTCGGCTTAACTCAGGCG
aacA30<br>*AAC(6')-I30*	TTAGGCTGGCGCGCTTCGCGCGGAAGACTTTATGGCTACTCGGAGACCTTAAATGGCATATGCGTTCTGCGAAATTGGAG<br>AATCAAATGAATATATTATTCAGGCAGCTAGAATCTTAACGAAATCATTCCTTGATATTGGAAATGATTCCTGGCCTGATA<br>TGAAAAGTGCCACCAAAGAAGTTGAAGAATGTATTGAGAAGCCAAACATATGTCTTGGAATACATGAAAACGAAAAACT<br>ACTTGGATGGATTGGTCTTAGGCCCATGTACAAATTAACATGGGAATTACATCCCTTGGTAATAAGTACGCAATATCAGA<br>ATAAAGGTATTGGAAGACTTCTAATAAATGAATTGGAAAAACAAGCAAAGCAAAATGGAATAATCGGAATAGTATTGGG<br>AACTGACGATGAATACTTTAAGACTTCATTATCAGATGTGGATCTTTCCGGGAAAAATATACTTGATGAGATAAGGAATA<br>TTAAAAATATAAGGAATCATCCGTACGAATTCTATCAACGATGTGGTTATTCCATTGTCGGAGTAATACCCGATGCAAAT<br>GGCAAAAGAAAGCCAGATATTTGGATGTGGAAGAAGATTAGTGATTAGGGCAAAAACGACAGCCTAACAATCGCTTTCA<br>CCTCGACAACCCGGCCTGTCACGCAATCTGCTAGTTTGATCCATGAGGCAGATTGCGCGCCAGGCTCGGGTTGCGGGTGA<br>AGCGTATG
aacA31<br>*AAC(6′)-31*	TTAGGCAGCACAAAGACCGTTCTCATGACCACTACCATTAGCTTCGTCACGCTGCGCCTCATGACCGAGCACGACCTTCC<br>GATGCTCCATGACTGGCTAAATCGGCCTCACATCGTTGAGTGGTGGGGCGGAGAAGAAACACGTCCAACACTTGCTGAAG<br>TGCTGGAGCAATACCTACCAAGCGCCCTGGCGAAAGAGTCCGTCACTCCCTACATCGCAATGCTGGATGAAGAACCGATT<br>GGGTACGCTCAGTCGTACATTGCACTCGGAAGCGGTGACGGATGGTGGGAAGACGAAACCGATCCAGGAGTACGCGGAA<br>TAGACCAGTCTCTGGCGAATCCATCGCAGCTGGGCAAGGGCTTGGGAACCAAGCTCGTTTGCGCGCTCGTTGAGATGCTG<br>TTCAAAGACGCTGAGGTAACCAAGATCCAAACGGACCCGTCGCCGAACAACTTACGCGCAATCCGGTGCTACGAGAAGG<br>CGGGTTTTGTGGCGCAAAGAACCATAAACACCCCAGATGGACCGGCCGTATACATGGTTCAAACACGTCAGGCGTTCGAG<br>CAGGCGCGCAGTGCTGTCTAACCCTTCCATCGAGGGGGACGTCCAAGGGCTATCGTCCTTGGCCGCCCCTCATGTCAAAC<br>G
aacA32<br>*AAC(6′)-32*	TTAGGCAGCACAAAAGGACCGTCCCATGTCCCCGAGCAAAACACCCGTTACCTTGCGCCTCATGACCGAGCGCGACCTAC<br>CGATGCTGCATGCATGGCTGAACCGGCCGCACATTGTCGAGTGGTGGGGTGGAGAAGAAGAACGCCCGACTCTTCATGA<br>AGTGGTCAAACACTACCTGCCGAGGGTTTTGGCAGAAGAAGCCGTCACACCATACATCGCGATGTTGGGCGACGAACCC<br>ATCGGCTACGCTCAGTCATACGTCGCACTCGGAAGCGGTGATGGATGGTGGGAGGATGAAACCGACCCAGGCGTACGAG<br>GGATAGACCAATTCCTGTCGAACCATACACAGTTGAACCAGGGCCTAGGTACAAAGCTCGTCCAGGCACTCGTTGAACTG<br>CTGTTCTCAGATCCTACCGTGACGAAGATCCAAACCGACCCGGCGCCAAACAACCATCGAGCGATTCGCTGCTACGAGAA<br>AGCTGGCTTTGTTCAGCAAAACGTCATCACCACACCAGACGGCCCAGCCGTCTACATGGTTCAAACCAGGCAGGCCTTCG<br>AGCGTGTGCGCAGTGCTGCCTAACCCCTCGCTCAAGCGGAGCGCCAACGGCAGGCCACCAGGCCCGGGCCGGTGGTACG<br>CTGTACATTTTCACCGGCCCGGGCCTGGCGTCCTGCCGTTGGCGCCCGCTTAGCTCGAACG
aacA34	TTAGAAGGCCCAGGCTATGCAGTACTCCATTCGCTCGGTTCGTGTTTCGGATACATCTGATTGGTTACGCCTTCGCAATCT<br>CCTGTGGGAAGGGGATGACCACGAAACCGAGATCGCCCAGTTTTTCGCCGGAGCCCTGGCCGAGCCCAACGAAGTGCTG<br>GTAGCCCATGATGATGCGGGGGCCGTTGTTGGGCATGTCGAGTTATCCATCCGCGAGGATGTCGCAGGGCTGGAAGGCAT<br>CAGAGCGGGCTATATCGAAGGCCTGTACATCGAGGAGGCCCATCGCTCGTCCAGCGTCGCGACGCAGTTACTACGGCACT<br>CCGAGCAATGGGCCCAAAGTCAGGGATGCCGGGCGTTTGCATCGGATCGAGAGGATCGCCTGATCATCCATAAGCGGTTT<br>TCTGTGAGTCCGCTTTCTAACCCTTCATTCCAGCGGACCGCCTTCGGCGGCCGCTGAATTTGAACG
aacA35	TTAGGCAGCACAGGGCCACCTTCTTATGCCCTCCCACGATCATCCTGTCACCTTGCGGCTTATGACGGAGCAAGACCTGCC<br>TATGCTCCATGAATGGCTGAATCGGCCGCACATAGTCGAGTGGTGGGGCGGTGAAGAGCAACGTCCGACACTGGCGGAT<br>GTACTGGAACACTACAGGCCCAGAATCTTGGCGCACGAGTCGGTCACTCCATACATCGCCATGCTGGGCGAAGAACCAAT<br>CGGATATGCGCAGTCGTACGTCGCGCTCGGAAGCGGCGATGGATGGTGGGAAGAGGAAACCGACCCAGGAGTACGAGGA<br>ATCGATCAGTCGTTGGCGAATCCTACGCAGTTGAACATGGGCCTGGGAACAGAGCTTGTGCGAGCGCTGGTTGATCGGTT<br>GTTCTCCGACCCAACGGTGACAAAGATCCAGACTGATCCGGCCCCAAACAATCGCCGCGCGATCCGCTGCTACGAGAAG<br>GCGGGCTTTGTGCAGGAGAGAGTCATCACGACACCCGACGGGCAGGCCGTCTACATGACCCAATCTAGGCAAGCCTACG<br>AGCGTGCGCGCGGTGCTGCCTAACCCCTCGCTCAACCCCCGACTCGCTACGGCAGGCGTCGTAAGCCCGGTCCGCGCCAG<br>CCGTAACATCATCGCGTACCGGGCTTACGACACCTGCCTCCGCTCGCGGGGTTAGCTCGAACG
aacA37	TTAACCGCGGCTATGAAGTACTCTTTAAATCCAATCCGTCCCGAAGAATCACACGATTGGCTGCGCCTTCGAGATCTGCTT<br>TGGGAAGCTGATGACCATGCGATAGAAATTGCGAGATTCTTTACTGGCGAGCTAGAGGAAACGGTTGAAGTACTGATCGC<br>ACGCGACCTCGAAGGACGTGCGGTAGGGCACGTTGAGCTTTCCATCCGTGAAGACATAGATGGTCTGAATGGCGTCAAGA<br>CCGGATACATCGAGGGGTTGTACGTTGATGTATTGCATCGCTCAAGCGGTCTGGTCAGGCAGTTTCTGAGAGCATCGGAA<br>CAATGGGCAAAAGATCAAGGCTGTAGCGCATTCGCATCTGATCGGCAAGACCGAGTAATCATCCACAGGCGGTTCTCCGG<br>TGGCTCCGCCTAACAATTCATTCAAGCCGACGCCGCTTCGCGGCGCGGCTTAATTCACGCG
aacA38	TTAGGCAGCACATAACCACCGTCACACCATGCCTACCAAAGCGATCGCTGTCACCTTACGCGCCATGACCGAGGATGATC<br>TTCCGATGCTGTACGACTGGCTCAACCGACCCCACATCGTTGAATGGTGGGGAGGCGAGGGGCAGCGTCCCACCCTCTCA<br>GACGTGGTGGAGCACTACCGGCCACGAGTCTTGGCAGAAGAGCGCGTCACGCCGTACATCGCCATGCTTGGCAATGAAC<br>CAATCGGCTATGCGCAGTCGTACATTGCTCTCGGGAGTGGCGAAGGTTGGTGGCAGGACGAAACTGACCCTGGAGTTCGC

	GGCATCGATCAATCGCTTGCCAATCCCCTCCAGTTGAACAAAGGTCTCGGAACGGAGCTTGTGCGATCACTCGTCGAACT<br>GTTGTTCTTGGATCCCGAGGTAACCAAGATTCAGACTGATCCGGCTCCTACTAACCATCGTGCAATCAGGTGCTACGAAA<br>AAGCAGGGTTTGTGGTGCAGAACACAATCACTACCCCCGATGGTCCGGCCGTCTATATGGTTCAAACTCGTCACGACTTC<br>GAGCGAGCGCGCTGTGCTGCCTAACTGTTTGGTCAAGCTGAACGCCAAAAGCTGCGCTTTTGGTGCCCTCACTCCGCTTCG<br>CTCCTTGCGGCGCAGCTTACCGCGGGCG
aacA39<br>*AAC(6')-Iai*	TTAGCCGGACGCTTCGCGCAAAGAGGTATTATGAAATACACTATTATTGATATTAAAGATTCAGAAACGTACATTACTCA<br>AGCTGCAGAAATATTATTTGATGTATTTTCAGAAATAAGCCCAGAATCATGGCCAACACTCCAAAAAGCAAAAGAAGATG<br>TTATTGAATGTATAGAAGGTGAAAACATTTGCATTGGCATTATAATAAATAAAGAATTAATTGGATGGATTGGATTAAGA<br>GAAATGTATAAAAAAACATGGGAATTACATCCTATGGTTATCAAGAAAACACATCATAATATGGGATTTGGAAAAATACT<br>AATTAATGAAATAGAAAAAAAAGCAAGAGAAAGAAATTTAGAAGGTATTGTACTTGGAACAGATGATGAAACATATAGA<br>ACTTCATTATCAATGATTGAATTAAATAATGAAAATATTTTGCAAGAAATAAAGAATATTAGAAATTTAGAAAATCATCC<br>TTATGAATTTTATAAAAAATGTGGATATTGTATTATTGGTGTAATTCCAAACGCAAATGGAAGAATAAGCCAGATATAT<br>TAATGTGGAAAAATATTATGGAAGAAAATTGCGGCTAACAACTGCTTCAACCTGACTCACCTATTGGCATGGTTTATGCTT<br>TCGCTTCGCTTGGCATAAACCATGCCAATGTACGGTTCGCAGGTTAAGCAAATG
aacA40	TTAGGCAGCACAGTCCAGACTCCGCATGCCCTCCACTGCCCCTGAAGTCACCCAGCGACTCATGGTCAAGCGCGACCTGG<br>TAATGCTTCACGAATGGCTCAATCGCCCTCACATCGTTGAGTGGTGGGGAGGCGAAGAAGCCCGCCCGACGCTGCAAGA<br>AGTGCAATCGCACTACCTACCTCGCGTGTTGGCCGAAGAGGCGGTCACGCCGTATATCGCGATGCTCGGGAGTGAGCCAA<br>TTGGGTACGCGCAGTCTTACGTTGCATTGGGAAGTGGCGATGGGTGGTGGGAGGATGAAACAGACCCGGGTGTGCGCGG<br>AATAGATCAGTCGCTGGCCAATCCAGAACAACTGGGCAAGGGCCTCGGGACCAGGCTTGTTTGTACCTTGGTCAAGACAC<br>TGTTCAACGATCCATCCGTGACCAAGATTCAAACCGACCCGGCGCCAAACAACGTCCGCGCGATCCGCTGCTACGAGAAG<br>GCTGGCTTCAGACAACAGAAGGTCATCACCACGCCGGACGGCCCGGCCGTCTACATGGTTCAAACCCGCGCCTCTTTCGA<br>GGCAGCGCGCGGTGCTGCCTAACCCCTCGCCCAAGGCGCCGACGCGCTACGGCAAGCAGCGTAAGCCTAGTCCGCGGCC<br>TATACCGCATCATCCCGCACTAGGCTTACGCTGCTCGCCTCCGCGCGCAGCTTAGCTCGAACG
<u>*aacA42*</u>	TTAGGCTGACGCGCTTCGCGCGGAAGACTTTATGGCTACTCGGAGACTTTGAATGGCGTATGAGTTCTGCGAAATAGGTG<br>AATCAAACGAATATATTATTCTGGCGGCTAGAATCTTAACGAAATCATTCCTAGATATCGGTAATAATTCCTGGCCTGACA<br>TGAAAAGTGCTACTAAAGAAGTTGAAGAATGCATTGAGAAGCCAAACATATGTCTTGGAATACATGAAAATGAAAAATT<br>GCTTGGATGGATTGGCCTTAGGCCCATGTACAAATTAACATGGGAATTACATCCCTTGGTAATAAGTACTCAATATCAGA<br>ATAAAGGTATTGGAAGACTTTTAATAAATGAATTAGAAAAAAAGCAAAGCAAATTGGAATAATTGGAATAGTATTGGG<br>AACTGACGATGAATACTTTAAAACTTCATTATCAGCTGTTGATCTTTACGGCGAAAATATTCTTGATGAGATAAGGACTAT<br>TAAAAACATAAAAAATCATCCGTACGAATTCTATCAAAAATGTGGGTATTCCATTGTCGGAGTAATACCCGATGCAAATG<br>GAAAAAGGAAGCCAGATATTTGGATGTGGAAGAAGATAAATGATTAGGGTAAAAATGACAGCCTAACAATCGCTTTCAC<br>CTCGACAACCCGGCCTGTCACGCAATCTGCTAGTTTAATCCTTGTGGAAGATTGCGCGCCAGGCTCGGGTTGCGGGTGAA<br>GCGTATG
aacA43	TTAGCCAGACGCTTCGCGCCGAGGACAATTGATGATTTATAACATAATTAACATTGCTGATTCTGAAAAGAACAAGGAAG<br>ACGCTGCACGAATTCTATATTCTGCATTTCGCGGAAAGGGAAAAGATGCTTGGCCTACATTAGATTCAGCTCGTGAAGAA<br>ATAGCAGAGTGCATAGCAAGTCCTAATATTTGCTTGGGCATAACCTTAGATGATCGCTTAGTAGGGTGGGGCGGACTTCG<br>TCCCATGTATGAAACCACATGGGAATTGCATCCCTTAGTAATAGATCCTGATTATCAAGGTAATGGATTAGGAAGACTGC<br>TCCTATCAAAGATTGAGAGCACTGCAACCCACAAATAGAATAATTGGTATAATGCTTGGAACAGATGATGAGACATTGAGT<br>ACAAGTCTTTCAATGACTGATATAGATGAGTCTAATATTTTCCAAGAGATAAAAAATATAATTAATATAAAGAATCATCC<br>ATTTGAATTTTATAAAAAATGCGGGTACATCATTGTCGGTATAGTACCTAACGCAAATGGGTATAGAAAACCTGACATTT<br>GGATGTGGAAGAATCTAGAAAAGAAAAGTGGCTAACAATCGCTTCAAGCGGATAAAACGGCGTGTCACGGTTCTTGCTT<br>ACGCAAGAACACGCGCCACACTCGTTTTACCGCTTAAGCGTATG
aacA44	TTAGGCAGCACAAGACCACCTGTTCATGCCCGCGAACGAAAACACCGTAACCCTACGTCTGATGACTGAGCACAATTGGT<br>GATTAAATGAACATTGACAACAGAACCGTAACCCTACGTCTGATGACTGAGCACGACCTCTCGATGCTCCATGAGTGGCT<br>GAACCGGCCACACATAGTTGAATGGTGGGGCGGTGAGGACGAACGTCCAACACTGGACGAGGTATATGAACACTACCTG<br>CCAAGAGTTCTGGCGCAGGAATCGGTCACCCCGTACATCGCCATGCTCGGCAACGAACCGATTGGCTACGCGCAGTCGTA<br>CGTAGCGCTCGGTAGCGGTGACGGATGGTGGGAAGAGGAAACTGACCCAGGGGTGCGAGGAATCGATCAGTCGCTGGCC<br>AACCCAACGAAACTGAACAAAGGCCTCGGAACGAAGCTTGTACGCGCGCTGGCTGATCTGCTGTTCTCAGATGCGTCAGT<br>GACAAAGATCCAGACCGACCCGGCTCCGGGTAACCATAGAGCTATTCGTTGCTACGAGAAAGCGGGGTTTGAGAGGCAA<br>GGTACCGTAACCACCCCAGATGGTCCAGCCGTGTACATGGTTCAAACACGCCAGGCATTCGAGCGAACACGCAGTGATGC<br>CTAACCCTTCCATCGAGGGGGACGTCCAAGGGCTGGCGCCCTTGGCCGCCCCTCATGTCAAACG
aacA45	TTAAAAGGCTCAGCCAATGCTGTACCTCATCCACCCGGTCAGTAGTTCGGACTCGCTCGATTGGCTTCGCCTTCGAAACCG<br>GCTCTGGACTGGACACGACCACGCGGAAGAAATTGCTGAATTCTTCAACGGAAATTTGGTGGAGCTGGACGAGGTATTGA<br>TCGCACATGACGACACGGGCATGGCCGTAGCTCATGTTGAGTTGTCAATCCGACAAGACATTGTTGGACTGGAGGGAGTC<br>CGAACAGGTTACATCGAAGGGCTCTATATCGATGAATTTCATCGCTCGTCCGGCATCGCACTCCAACTCTTGCGGGCGTCC<br>GAGCTTTGGGCTCTGGATCACGGGTGCCAAGCATTCGCTTCTGACCGCGAAGATCGCATCATCGTTCACAAGCGGTTTCCT<br>GGAACTCCGCCTTCTAACATTTCAGTCGACCGGACCGCCTGCGGCGTCCGGTCACCTTCACGTTGGGCATTAAGGAAAAG<br>TTAATGGCAATCCGAATCTTCGCGATACTTTTCTCCATTTTTTCTCTTGCCACTTTCGCGCATGCGCAAGAAGGCACGCTAG
aacA46<br>*AAC(6')-Iaj*	TTAGGGCGACGCCGCATTCGCGGCGCGTGAAGAAAGAGGATCTTATGGAATATTCAATTATCAATATAGTAGAGCAAAAC<br>AATTATCAGATCGATGCTGCAAGAATTCTTACAAATACTTTTCTTGATATAGGTAATAAAACTTGGCCAACTATTCAAAGC<br>GCAATCGATGAAGTCGAAGAGTGTATTGATCTGCCCAATATATGTATAGGTTTAATTCATAACAATCAATTAATTGGATG<br>GGTCGGATTACGTCCGATGTATGATAAAACGTGGGAATTGCACCCATTAGTTGTAAGAACTGACTATCAAAGTAAGGGGA<br>TCGGTAGTGTATTACTTGCTGAAGTTGAAAAAAGGGCAAGAGAAGTTGGAATAATTGGAATAATATTAGGAACTGATGAT<br>GAATATAACAAAACAAGTCTTTCTGAAATAACTATAGATGAAAATAATATATTCGATGCAATACAAAATATTAAGAATAT<br>ACATAATCATCCATATGAGTTTTACCAAAAAAATGGATATATGATTGTTGGAATAATTCCAAATGCAAATGGACTAAGAA<br>AACCCGATATTTGGATGTGGAAAAGTCTACTCAATTGAATGGAAACAATAATAACTATAGATAAGCTGTAAAAAATACTT<br>TGACGAAATAACTTCGGTTGAAGTTATTATAGAAAAGAATATTAAAAAGAAAATTGTAAAAGAAATAATTTTTTATCAAA<br>AAATATCATTGATTCTTCTGGAAGAAACAGAAAAAGAAATGATTTACTTTGGTAATAATCTACTTAATACACAATAGTTTT<br>ATTCAAAAATAATGTTCATTAGGTTATAGGAAATTTGCCCTAACAACTGTTTCAACCTGACATTGCTATTGTCACACTTTTT<br>GCTTTCGCTTCGCGGCAAAAAGTGCGCCAATTGACGCAATGCAGGTTAAACAAATG
aacA47	TTATGCATCACAGAACCACCATACCTATGTCCGCGATCGACACCCCCGTTACCTTGCGCCTCATGACCGAGCAAGATCTTC<br>CGATGCTCCATGACTGGCTGAACCGACCCCACATATTCGAGTGGTGGGGCGGTGAAGAGGAACGCCCGACTCTTGATGAA<br>GTGCTGGAACACTACCTGCCAAGAGTTCTGGCAGAAGAGTCCGTCACGCCGTACATCGCAATGCTGGGCGAGGAGCCGA<br>TCGGCTACGCCCAGTCTTACGTCGCACTCGGCAGCGGTGATGGATGGTGGGAAGACGAAACTGACCCAGGAGTGAGGGG<br>AATTGACCAGTCTCTGGCTAATCCGACACAGTTGAGCAAGGGTCTGGGAACAAAGCTTGTCCGTGCGCTCGTTGAGCGGT<br>TGTTCTTGGACTCCACTGTGACGAAGATCCAAACCGACCCAACTCCGAACAACCATCGGGCGATCCGTTGCTACGAGAAG<br>GCGGGGTTTGTACGGCAGAAGATAATCACCACGCCTGATGGCCCAGCCGTCTACATGGTTCAAACACGTCAGTCATTCGA<br>GAACGCGCGTAGTACTGCCTAACCAGCCAATGGAGCCGATGCGGTGACGTATTGCGAGGTTTTAGTTCGGTGGCCCGCGC<br>GGCTCATCGGCAACG
aacA48<br>*AAC(6')-Iag*	TTAGGTCCCACTAAACCTGCACCGAGCATGGCGAACACTCCGGTTGGAAACGTCGTGCCATGCAAGACGCCAGATCACCC<br>TGGCTGGCTTGAGTTGCGCCTGCAGCTGTGGCCAGATGGCTCAACCGAGGAGTTCCTTCCCGAGATGGCTGCGGCCTGCG<br>CTGAACCCGACCGCTTCGGCCAGTTTTTGTTCCTGTCGCCGGGCGGCTTGGCGGAGGGCCTGGTCGAGGTGGCGCTTCGC<br>ACGGACTACGTCAACGGCACCGAAAGCTCGCCAGTCGCCTTTCTCGAAGGCGTCTTCGTGGTGCCAGCGAGTCGAGGCCT<br>TGGCATCGCCAGAGCGCTGGTGGCTGCGGCGGAAGGCTGGGCTAGAGATCGTGGCTGCACTGAGTTCGCCTCGGACGCC<br>GAGGTCAGCAACGTTGGTAGTCACGCCATGCACGCCGCTCTTGGTTTCGTCGAAACTGAGCGCGTCGTGTTCTTCCGTAAG<br>GTCGTGGCACCGTGAGACCTAACCCCTCCATAGAGCGGACAGCCAAAAGCTGGCCGCTCATGTCGAACG

aacA49	TTAGCTTGACGCTTCGCGCAGAGGAGAGTTTCAATGAATATACAAATTTTGAACCTAGCAGAATGTACCGAATTTCAAGA<br>GAGTGCAGCAAGAGTCCTATTAGATGGATTTAGAGAGGTTGGCAAGATTGCATGGGCTACCTATGAAGAAGCTATGGTCG<br>AAGTACAGGAATGTACTGAGATTCCTAATATAGCAATATGTGCTGTCGATAATAATAAAGTCGTTGGTTGGGTTGGAATT<br>CGTCCAATGTACGATTACGTTTGGGAACTACATCCAATGATAGTAACCAAGAAATACCAAAAAAAAGGAATAGGACCA<br>AACTACTTAAGGAAATTGAACAGATTGCGAAAGAGAAAGGTTTACTAGGACTGGCTCTTGGAACTGATGATGAAACTGAT<br>AGTACAAGTCTATCGAAATGCGATTTTTCCCGAGATAATATATTGATCGAGATAGCGAATATCAAATCATCTATGTCGCAC<br>CCCTATGCGTTCTATGAAAAGAACGGATACTTTATTGTAGGAGTGATTCCGAATGCGAATGGAAAAAGAAAACCAGACAT<br>TTGGATGTGGAAAGAACTTGAAAGCTAACATCTAGTTCAACTTGACTTATTTAGCCGTCACGGGTTCTTGCTTTCGCAACA<br>ACCGCGGCCAGCCAAAACGCAAGTTAATATAGGCG
aacA50	TTAGACAGCACAAAGACAATTCTCATGATCAACAGCATTAGCCTCGTTACGCTGCGCCTCATGACCGAGCAAGACCTACC<br>GATGCTCCACGACTGGCTAAACCGGCCGCACATCGTTGAGTGGTGGGGTGGAGAAGAGGCACGGCCATCACTCGCTGAG<br>GTACACGAGCAATACCTACCAAGTGTTCTGGCGAAAGAGTCGGTTACTCCATACATCGCAATGCTGAATCAAGAGCCGAT<br>CGGGTACGCTCAGTCATACGTTGCCCTCGGAAGCGGTGACGGATGGTGGAAGACGAAACCGATCCAGGGGTGCGTGGA<br>ATCGACCAGTTCCTCGCGAGTCCACTACAACTTGGAAAAGGCCTTGGAACCAAGCTCGTTCGCGCGCTGGTAGAGACGCT<br>GTTCAAAGATCCCGAGGTAACCAAGATCCAAACGGACCCGTCGCCGAGTAACCTACGCGCGATCCGCTGCTATGAGAAG<br>GCGGGGTTCGTGAGACAAAAGACAGTAGCCACGCCAGATGGGCCAGCTGTGTACATGGTTCAAACACGCCACGAGTTCG<br>GGCGGGCGCGCAGTGCTGCCTAACCCTTCCATCGAGAGGACATGCCCCGGCAAGCCGGGTCATGCCTCTCATGTCAAACG
aacA51	TTAGGCCACAAGGAACCGTCCCAGTATGAGCCCCGGCGTTGTCACCTTGCGGAGCATGACCGAAGAGGATCTCGGTATGC<br>TTCACGACTGGTTGAACCGGCCCCACATCGTCGAATGGTGGGGTGGCGAGCGTCCTTCGCTTGAGGAGGTGCAAGAGCAC<br>TATCACCCTTGTGCCCTTGCAGAAGCCAACGTGACTCCGTACGTCGGGATGCTCGATGGGCGGCCTTTTGCATATGCACAG<br>TCGTATGTCGCCCTGGGATCGGGAGATGGGTGGTGGCAGGACGAAACCGATCCGGGCATACGTGGTATCGACCAGTCAAT<br>TGGCGAGTCCGCGCTTCTAGGGCAAGGTTACGGTACGTTGCTGGTACGCGCGCTCGTTGATCTGCTCTTCGCCGACCCGCG<br>CGTATCGAAAGTTCAGACAGATCCTTCTCCCCGGAACTTGCGCGCCATACGATGCTATGAGAAGGCTGGCTTCCGCAGGA<br>TCAAGACCATTGAGACACCTGATGGACCAGCGATATACATGTTGCACGAGCGCCCATAGCCAGGGAATCCGCACGGTGT<br>GGCCTAACAGATCGCTGCAACGGACAGTTGACCGAGCCGCTCGCGCGCTACCGCACGCGATCGTCCGCGTCAACTGCCGC<br>TGAGCTCCGGAG
aacA52	TTAGGCAGCACAAGATGACCGCGAACGAAAACACCGTAACCCTGCGCCTGATGACTGAGCACGACCTCCCCATGCTCCAT<br>GAATGGCTGAACCGGCCACACATAGTTGAATGGTGGGGCGGTGAGGAAGAACGTCCAACACTGGACGAGGTATATGACC<br>ACTACCTGCCAACAGTTCTGGCGCAGGAATCAGTCACCCCGTACATCGCCATGCTAGGCAACGAACCGATTGGCTACGCG<br>CAGTCGTATGTCGCGCTTGGCAGCGGTGACGGGTGGTGGGAAGAGGAAACCGACCCAGGCGTGCGAGGAATCGATCAGT<br>CGCTGGCCAACCCAACGAAACTGAACAAAGGCCTCGGGACCAAGCTTGTACGCGCGCTGGCTGATCTGCTGTTCTCAGAT<br>GCGTCAGTGACAAAGATCCAGACCGACCCGGCTCCGGGTAACCATAGAGCTATCCGTTGCTACGAGAAGGCAGGCTTTTT<br>GCAGGAAAAGGTCATCACCACACCTGACGGGCCGGCTGTCTACATGGTTCAGTCAAGGCAAACATACGAGCGTGCGCGC<br>GGTGCTGCCTAACCCCTCGTTCAAGCGGACCGCCAACGGCGTGGCGCCTTGGCGGCCGCTTAACTCGAACG
aacA54	TTAGGCCGCACAAAATCAACGCCTTATGTACACCAAGAATGCCGCAATAGTTCTGCGCCTCATGACTGAGAGCGATCTGC<br>CAATGCTCCATGCGTGGCTGAACCGGCCCCACATAGTCGAGTGGTGGGGAGGAGAAGATAAACGCCCCACACTTGGCGA<br>AGTTTTAGAACATTATTCGCCCCGAGTTCTCGCAGAGCAAGCAGTAGTACCCTACATCGCAATGCTAGATGATGAACCCA<br>TTGGCTACGCGCAGTCCTACACCGCCCTTGGAAGTGGCGATGGATGGTGGGAAGACGAAACTGATCCAGGGGTCCGTGGC<br>ATTGACCAGTCATTGGCCAATCCGTCGCAGCTAAACAAGGGCCTTGGAACAACGCTCGTACGCTCACTGGTTGAACTCCT<br>GTTCAGTGATCCGGCCGTGTCGAAGATCCAAACTGATCCTTCTCCTAACAACCATCGCGCTATCCGCTGTTACGAAAAAG<br>CCGGGTTCGCACAAGACAAAATCATCCTGACGCCTGACGGCCCTGCGGTGTATATGGTTCAAACACGCCAAGCGTTCGAA<br>AGCCAGCGCAATGCTGCCTAAAATTGAGGTCAAGGCGGCTCGCGTTCATGAAGAGCCTCATCATCACTCTATCCATTCGC<br>ATCTCGGCGTGTTTTCCTACGCCTTGGCATCTGTCCCTAGCCTAACTGGGCGCTCAACTGGACATCAACGTGCTGCGCACG<br>TTGCTGCCAGTTAACTGGGGCG
aacA56	TTAGCCGGACGCTTCGCGCAGGAGTAAGAATGGAATACAAAATAGTAGATATTGCACTTGATAGTAAGCTGGTGAAAGTT<br>GCAGCAGAGATTTTGTTTTATACTTTTTCTGAAATTAATAATGAATCTTGGCCAACAATTAACTCTGCCACTGAGGAAGTG<br>AAAGAATGTATAGAAGATAAGAATATTTGTATTGGAGTTTTGGTAGAAGACAAGCTGGTAGGTTGGATTGGATTACGTCC<br>AATGTATGAAAATACTTGGGAATTACATCCAATGGTGGTTTTATCAAAATGGCAAGGCAAAGGATTAGGGAAAATATTAA<br>TATTTGAATTAGAAAAAAGAGCAAAAGAACAAGGAATAAACGGAATTGTTTTAGGAACTGACGATGAGACATTTCGAAC<br>ATCTTTATCTATGAAAGAATTGGATAAAAATGATCTATTTGAAGAAATAAAAAACATTAAAAATATTAATCATCATCCGT<br>ATGAGTTTTATCAGAAATGTGGATATAAAATTATTGGAGTAATCCCAGATGCTAATGGTAAGAATAAACCGGATATTTGG<br>ATGTGGAAGAAAATAATGTAGGAAGAACGGCTAACAACGGTTTCAACCTGACTTGCCCTTTGTCACGGTTTGTGCATGAC<br>GCTTCGCGGCTGCGCCGCTTCGCTTCTTCATGCACAAACCGCGCCAATCCGCTTCGCGGAACGGGCTGCGCAGGTTAAAC<br>CAATG
aacA59	TTAGGCAGCACAGAAGCCGCATCCCATGCCCGCGAGCACATCCGTTGTGACCCTGCGCCTGATGACCGAGCACGACCTGC<br>CAATGCTCCACGAATGGCTGAACCGGCCGCACATCGTCGAGTGGTGGGGCGGCGAGGAAGAGCGCCCCAGTCTTGACGA<br>AGTCCGCGAGCACTATCTTCCGAGAGTCTTGGCTGAAGAGGCGGTTACGCCGTACATCGCAATGCTGGACGGCGAGGCAA<br>TTGGCTACGCCCAGTCTTACGTCGCACTGGGAAGCGGCGATGGGTGGTGGGAAGACGAAACTGATCCGGGAGTACGAGG<br>GATTGACCAGTCGCTGGCCAACCCCACACAGTTGAGCCGGGGCCTCGGAACGCAGCTTGTCCGTGCGCTCGTAGAGATGC<br>TGTTCTCCAACCCCGCCGTGACTAAAATTCAGACCGATCCCGATCCCAAGAACCTACGTGCAATCCGGTGCTATGAGAAG<br>GCAGGGTTTGTACAGCAGAAGGTCATCACCACGCCCGATGGCCCCGCCGTCTACATGGTTCAAACGCGCAACGCGTTCGA<br>GAGTTCGCGCAGTGCTGCCTAACACCTCGTTCAACCCCCGACCCGCTACGGCAGGCACTGTAAGCCTGGCCCGAGGTACT<br>CCGTACATTGTCTCGGTCCAGGCTTACAGTGCCCGCCTCCGCGGTCGGGGTTAACTCGAACG
aacA61	TTAGGCAGCACAGGGCCACCGCTTATGTCCTCCAGCGAATCCAAAGTCACGTTGCGCCTCATGACCGAGCACGACCTGCC<br>AATGCTCCACGACTGGCTAAACCGGCCTCACATCGTTGAATGGTGGGGCGGTGAGGATGAGCGCCCTACTCTCGACGAAG<br>TACTCGAGCACTACCTGCCACGAGTAATGGCGGAGGAATCAGTAACTCCATACATTGCCATGCTGGGCGATGAACCGATC<br>GGCTACGCACAGTCTTACGTCGCACTCGGGAGCGGTGATGGATGGTGGGAAGACGAGACCGACGCAGGGGTACGAGGAA<br>TTGACCAATCTCTAGCCAATCCAGCGCAGTTGAGCAAGGGCCTGGGAACACTCCTGGTCCGAACGCTCGTTGAAACCTTG<br>TTCGCGGATCCGGCCGTCACGAAGATCCAAACCGATCCGTCTCCAAACAACTACCGCGCAATCCGCTGCTACGAGAAGGC<br>AGGGTTTGCACAGCAGGGCGTCATCACCACGCCTGACGGGCCGGCGGTCTACATGGTTCAAACACGGCAAGCGTTCGAG<br>CGCGCGCGCGGTGCTGCCTAACAACTCGCTCAACCGGACCCGCTACGGCAGGCCGGTTAGCTCGAACG
aacA64	TTAGCCGGACGCCTTCGGCGCTAGGAATAAAATGGAATATTCAATTGTTAATATTGGCTTAAACGATAATTATATTACCCA<br>AGCAGCTGTTGTTTTATATAATGTATTTAATAATCTTGAAAACCAATCATGGCCAACTATTGATTCTGCCAAAATGGAAGT<br>AAATGAATGCTTAGAAAATAATAACATCTGTATTGGAATGTTGATAGATAATAAAATAGTCGGTTGGGTAGGCCTAAGGC<br>CAATGTATGAAAAAACTTGGGAATTACATCCATTAGTTGTCATTGATGATTATCAAAATATGGGTGTTGGTAAAATATTAA<br>TGAATGAAATAGAAAAAAGGGCAAAAGAAAAGGGAATTATAGGGGTTGTACTTGGAACTGATGATGAGAAATATAAAA<br>CATCGTTATCAAAAGTTGATTTAAATAATAAGAATATATTCACTGAAATAGAAAATATTAAAAATATTAATCATCATCCAT<br>TCGAGTTTTATCAAAAATGTGGATATTTTATTGTTGGTGTAATACCAAATGCAAATGGAAAAAATAAGCCAGATATATGG<br>ATGTGGAAAGAAATACAATAAGAAAGTTCGGCTAACAACTGCTTCAACCTGAGTCGCCTATTGTCATGATTTTTGCTGCTC<br>GCTTCGCTCGTTCACGCAAAAATCACGCCAATTTACGGCTCCCAGGTTAAGCAAATG
aacAX	TTATGCATACAAATCATCACCGTGATTTACTCTTACCGGAAAGCTGAAGAAACAGATAGAGAAGCCATCTACCAATTGTA<br>TTGCTTGGTAATGCGCGGCTTCATTTCTGAAATTTGGGGTTGGGATGAACAGTGGCAAGGAAACGATTTTTCTGCTCACTT<br>TGATACCAAAGGCATTACGTTGGTACACCAAAAACACGAGTTGGTTGGGTATTCCCATGTCGAGAATCGAGGGGCCAGTG<br>TCATAAGAATGATCGTTGTTCATCCTCATCACCAGCGGAAGGGTATCGGGAGAAAACTGCTTGAGTCTGTTATTGCGTCTG<br>GCAATGAGCAATCCAAGGGCATCGGATTGGAGGTATTCAAAATCAATGATGAGGCAAAGAAATTCTATGAAAGATTTGG

	CTTTAATGTTGAAGGTGAAAACCCCACCAGTTACGTCATGGCACATGCATAACCCGGCGCTCAACCTCGCTCCCTTCGGTC<br>GCTGGACTCCGGGCGATAAGGCCGCCCGGAGCCGGTTAGCTCTACG
aacC1<br>*AAC(3)-Ia*	TTAGGTGGCTCAAGTATGGGCATCATTCGCACATGTAGGCTCGGCCCTGACCAAGTCAAATCCATGAGGGCTGCTCTTGA<br>TCTTTTCGGTCGTGAGTTCGGAGACGTAGCCACCTACTCCCAACATCAGCCGGACTCCGATTACCTCGGGAACTTGCTCCG<br>TAGTAAGACATTCATCGCGCTTGCTGCCTTCGACCAAGAAGCGGTTGTTGGCGCTCTCGCGGCTTACGTTCTGCCAAAGTT<br>TGAGCAGGCGCGTAGTGAGATCTATATCTATGATCTCGCAGTCTCCGGCGAGCACCGGAGGCAAGGCATTGCCACCGCGC<br>TCATCAATCTCCTCAAGCATGAGGCCAACGCGCTTGGTGCTTATGTGATCTACGTGCAAGCAGATTACGGTGACGATCCC<br>GCAGTGGCTCTCTATACAAAGTTGGGCATACGGGAAGAAGTGATGCACTTTGATATCGACCCAAGTACCGCCACCTAACA<br>ATTCGTTCAAGCCGAGATCGCTTCGCGGCCGCGGAGTTGTTCGGAAAAATTGTCACAACGCCGCGGCCGCAAAGCGCTCC<br>GGCTTAACTCAGGCG
aacC2<br>*AAC(3)-Ib*	TTAGGTGGCTCAATGAGCATCATTGCAACCGTCAAGATCGGCCCTGACGAAATTTCAGCCATGAGGGCTGTGCTCGATCT<br>CTTCGGCAAAGAGTTTGAGGACATTCCAACCTACTCTGATCGCCAGCCGACCAATGAGTATCTTGCCAATCTTCTGCACAG<br>CGAGACGTTCATCGCGCTCGCTGCTTTTGACCGCGGAACAGCAATAGGTGGGCTCGCCGCCTACGTTCTACCCAAGTTCG<br>AGCAAGCGCGAAGCGAGATCTACATTTATGACTTGGCAGTCGCTTCCAGCCATCGAAGGCTAGGAGTCGCAACTGCCCTG<br>ATTAGCCACCTGAAGCGTGTGGCGGTTGAACTTGGCGCGTATGTAATCTATGTGCAAGCAGACTACGGTGACGATCCGGC<br>AGTCGCTCTCTACACAAAGCTTGGAGTTCGGGAAGACGTCATGCACTTCGACATTGATCCAAGAACCGCCACCTAACAAT<br>TCGTTCAAGCCGAGACCGCTTCGCGGCTCGGCTTAGTGCGGTAACGTGTACCACACCGCCGTGCCGCGCAGCGGTCCGGC<br>TTAACTCAGGCG
aacC3<br>*AAC(3)-Ic*	TTAGGCAGCAGCAGCTAAGATGATCTCTACTCAAACCAAGATTACCCGCCTCAACTCTCAAGACGTTGGTGTAATGCGGG<br>CAATGCTAGGCATGTTCGGCGAGGCTTTTGAGGACGCTGAGAACTATTGCCGCGCTCAACCAAGCGACAGTTACCTACAA<br>GACTTACTGTGTGGCTCTGGCTTCATCGCAATCGCTGCGTTACAGGGGCAAGAGGTCATCGGTGGGCTCGCCGCGTATGT<br>GCTCCCAAAGTTTGAACAACAGCGCAAAGAAATCTATATCTACGACTTAGGCGTGCAGGGAGCCTATCGCCGACGAGGC<br>ATCGCCACAGCCTTGATCAATGAACTCCAGCGTATCGCACATGATATTGGCGCTTATGTAATTTTTGTCCAGGCTGACTAT<br>GGGGACGATCCTGCGGTAGCGCTCTACACAAAACTCGGTATCCGGGAGGACGTGATGCACTTTGACATAGAACCTCAACC<br>TGCTGCCTAACAAGTCGTTCAAGCCGACGCTGCTTCGTGGCAGCAACTGCGTGCATACGCTACGCTAGCACGCGTTGCCG<br>GCACTACCGCAGCGCGGCTTAACTCAAGTG
aacC4	TTAGGTGGCTCACGTATGGGCATCATTCGCACATGTAGGCTCGGCCCTGACCAAGTCCAATCCATGCGGGCTGCTCTTGAT<br>CTTTTCGGTCGTGAGTTCGGAGACGTAGCCACCTACTCCCAACATCAGCCGGACTCCGATTACCTCGGGAACTTGCTCCGT<br>AGTAGGACATTCATCGCGCTTGCTGCCTTCGAGCAAGAAGCGGTTGTTGGCGCTCTCGCGGCTTACGTTCTGCCCAAGTTT<br>GAGCAGGCGCGTAGTGAGATCTATATCTATGATCTCGCAGTCTCCGGCGAGCACCGCCGGCAGGGCATTGCCACCGCGCT<br>CATCAATCTCCTCAAGCATGAGGCCAACGCGCTTGGTGCTTACGTGATCTACGTGCAAGCGGATTACGGTGACGATCCCG<br>CAGTGGCTCTCTATACAAAGTTGGGCATACGGGAAGACGTGATGCACTTTGATATCGACCCAAGTTCCGCCACCTAACAA<br>TTCGTTCAAGCCGAGATCGCTTCGCGGCCGCGGAGTTGTTCGGTAAATTGTCAAAACGCCGCGGCCGCAAAGCGCTCCGG<br>CTTAACTCAGGCG
aacC5<br>*AAC(3)-Id*	TTAGGCATCAGGAGCAGACGAGTGTCAGTCGAAATCATCCATCTCACTGGAAACGATGTTGCGTTGTTGCAGTCAATAAA<br>TGCCATGTTCGGCGAGGCATTCAACGACCAAGATAGTTATGCCCGCAACAAGCCGTCATCAAGCTATCTTCAAAAACTGC<br>TTAGCACTTCTAGTTTTATTGCGTTGGCTGCGGTTGACGAGCAAAAAGTCATTGGCGCTATCGCCGCGTATGAGTTGCAAA<br>AATTCGAGCAGCAAAGAAGCGAGATTTATATCTACGATCTCGCTGTAGCGGCAACCCGCCGCAGAGAAGGCATAGCTAC<br>AGCTCTAATTAAAAAACTCAAGGCTATAGGCGCAGCGCGTGGAGCTTATGTGATTTACGTCCAAGCTGATAAAGGCGTAG<br>AAGACCAACCAGCCATAGAGCTCTATAAAAAACTAGGAACCATCGAAGACGTATTTCATTTCGACATTGCGGTTGAGCAG<br>AGTAAAAATCATGCCTAACAAGGCACTCCAGCCGACGGCTTTGCCTTCGCTGCGCTACGGCAAATCCGCGGCTGAGTTTG<br>GGCG
aacC6<br>*AAC(3)-If*	TTAGGTGGCTCAATGAGCATCACTGCTACCATCAGGATTGGTCCTGATGAGATTCCGGCCATGCGGGCTGTGCTCGACCTG<br>TTCGGCAGAGAGTTTGAGGACATGTCCGCCTACTCGGATCGCCAGCCGACAAATGATTACTTGGCCAAGCTTCTCCGCAG<br>CGAGACGTTCATCGCGCTAGCCGCATTTGACCAAGGAACAGCAATAGGTGGGCTCGCTGCCTACGTTTTGCCCAAGTTCG<br>AGCAAGCGCGTAGTGAGATCTACATTTATGACTTAGCAGTCGCATCCAGCTATCGACGGCAAGGAATCGCAACTTCCCTG<br>ATTAGCCACCTGAAGCGTGAGGCAATCAAGATTGGGGCATATGTGATCTATGTGCAAGCAGACTACGGAGACGACCCCG<br>CGGTGGCTCTCTACACCAAGCTTGGTGTTCGGGAAGATGTCATGCACTTTGACATTGATCCAGGAACCGCCACCTAACAA<br>TTCGTTCAAGCCGAACTTGCTTCGTTACACCAGCGCCGTGGCAGGTTAAGCTTGCCACGTCGCCGGCTACACTTCGCAAGT<br>CGGCTTAACTCAGGCG
aacC11	TTAGGTGGCTCACGTATGGGCATCATTCGCACATGTAGGCTCGGTCCGGACCAAGTGCAATCCATGCGGGCTGCTCTTGAT<br>CTCTTCGGTCGTGAGTTCGGAGACGTAGCCAGCTACTCCCAACATCAGCCGGACTCCGATTACCTCGCGAAATTGCTCCAC<br>AGCAGGACATTCATCGCGCTTGCCGCGTTCGACCAAGAATCGGTTGTTGGCGCTCTTGCCGCCTACGTTTTGCCAAAGTTT<br>GAGCAAGCACGTAGCGAGATCTACATCTATGATCTCGCAGTCTCCGGCGAGCACCGGCGGCAAGGCATAGCCACCGCGC<br>TCATCAATCTCCTCAAGCAAGAGGCCAATGCGCTTGGTGCTTACGTGATCTATGTGCAAGCTGACTACGGTGACGATCCC<br>GCAGTGGCCCTCTACACAAAGTTGGGCATACGGGAAGACGTCATGCACTTTGATATCGACCCAAGTACCGCCACCTAACA<br>ATTCGTTCAAGCCGAACCTGCTTCGTGGCCCGGCTAATGTGCTAGCGTGTAGCACAAAGCCGTGCCACTACGCAGGTCGG<br>CTTAACTCAGGCG
aacC13	TTAGGCATTAGGAGCCGATGAATGTCAGTCAAGATTATTCAACTCACTGAAAAAGATGTTGGCTTAATGCAGTCTATAAA<br>TGCCATGTTCGGTGAGGCATTCGATGATAAAGAGCATTATTCCAGCAACAAGCCGACCTCAAGCTATCTTAAAAAACTGC<br>TTGGCAGTTCAAGTTTTATTGCGTTGGCTGCGGTTCACGAGGAAAAAGTCATAGGCGCTATAGCTGCGTATGAGTTGCAA<br>AAATTTGAGCAGCAAAGAAGCGAAATCTACATCTACGACCTCGCTGTAGCGGCAACCCACCGCAGAGCAGGCGTAGCTA<br>CAGCACTAATTCACAAATTGAAGGCGATAGGCGCCGAGCGCGGCGCTTATGTCATATACGTCCAAGCCGATAAAGGCGTA<br>GAAGACCAACCGGCCATAGAGCTTTATAAAAAACTGGGAACCATCGAAGACGTATTCCACTTCGATATTGCAGTTGAGGA<br>TAATAAAAATGCCTAACAAGGCGTTGCACCGGAAAAATTTGCCTTCGCTTCGCTACGGCAATTTTTCAGGTGAACTTGGG<br>CG
aadA1<br>*ANT(3'')-1*	TTAAACATCATGAGGGAAGCGGTGATCGCCGAAGTATCGACTCAACTATCAGAGGTAGTTGGCGTCATCGAGCGCCATCT<br>CGAACCGACGTTGCTGGCCGTACATTTGTACGGCTCCGCAGTGGATGGCGGCCTGAAGCCACACAGTGATATTGATTTGC<br>TGGTTACGGTGACCGTAAGGCTTGATGAAACAACGCGGCGAGCTTTGATCAACGACCTTTTGGAAACTTCGGCTTCCCCT<br>GGAGAGAGCGAGATTCTCCGCGCTGTAGAAGTCACCATTGTTGTGCACGACGACATCATTCCGTGGCGTTATCCAGCTAA<br>GCGCGAACTGCAATTTGGAGAATGGCAGCGCAATGACATTCTTGCAGGTATCTTCGAGCCAGCCACGATCGACATTGATC<br>TGGCTATCTTGCTGACAAAAGCAAGAGAACATAGCGTTGCCTTGGTAGGTCCAGCGGCGGAGGAACTCTTTGATCCGGTT<br>CCTGAACAGGATCTATTTGAGGCGCTAAATGAAACCTTAACGCTATGGAACTCGCCGCCCGACTGGGCTGGCGATGAGCG<br>AAATGTAGTGCTTACGTTGTCCCGCATTTGGTACAGCGCAGTAACCGGCAAAATCGCGCCGAAGGATGTCGCTGCCGACT<br>GGGCAATGGAGCGCCTGCCGGCCCAGTATCAGCCCGTCATACTTGAAGCTAGACAGGCTTATCTTGGACAAGAAGAAGAT<br>CGCTTGGCCTCGCGCGCAGATCAGTTGGAAGAATTTGTCCACTACGTGAAAGGCGAGATCACCAAGGTAGTCGGCAAATA<br>ATGTCTAACAATTCGTTCAAGCCGACGCCGCTTCGCGGCGCGGCTTAACTCAAGCG
aadA2	TTAGACATCATGAGGGTAGCGGTGACCATCGAAATTTCGAACCAACTATCAGAGGTGCTAAGCGTCATTGAGCGCCATCT<br>GGAATCAACGTTGCTGGCCGTGCATTTGTACGGCTCCGCAGTGGATGGCGGCCTGAAGCCATACAGCGATATTGATTTGT<br>TGGTTACTGTGGCCGTAAAGCTTGATGAAACGACGCGGCGAGCATTGCTCAATGACCTTATGGAGGCTTCGGCTTTCCCTG<br>GCGAGAGCGAGACGCTCCGCGCTATAGAAGTCACCCTTGTCGTGCATGACGACATCATCCCGTGGCGTTATCCGGCTAAG<br>CGCGAGCTGCAATTTGGAGAATGGCAGCGCAATGACATTCTTGCGGGTATCTTCGAGCCAGCCATGATCGACATTGATCT<br>AGCTATCCTGCTTACAAAAGCAAGAGAACATAGCGTTGCCTTGGTAGGTCCGGCAGCGGAGGAATTCTTTGACCCGGTTC<br>CTGAACAGGATCTATTCGAGGCGCTGAGGGAAACCTTGAAGCTATGGAACTCGCAGCCCGACTGGGCCGGCGATGAGCG<br>AAATGTAGTGCTTACGTTGTCCCGCATTTGGTACAGCGCAATAACCGGCAAAATCGCGCCGAAGGATGTCGCTGCCGACT<br>GGGCAATAAAACGCCTACCTGCCCAGTATCAGCCCGTCTTACTTGAAGCTAAGCAAGCTTATCTGGGACAAAAAGAAGAT

	CACTTGGCCTCACGCGCAGATCACTTGGAAGAATTTATTCGCTTTGTGAAAGGCGAGATCATCAAGTCAGTTGGTAAATG<br>ATGTCTAACAATTCGTTCAAGCCGACCGCGCTACGCGCGGCGGCTTAACTCCGGCG
aadA4	TTAGGCATCTTCATGGGTGAATTCTTTCCTGCACAAATTTCCGAGCAGCTATCCCACGCTCGCGGGGTGATCGAGCGCCAT<br>CTAGCTGCAACGCTGGACACAATCCACCTGTTCGGATCTGCGCTCGATGGAGGGTTGAAGCCGGACAGCAACATCGACTT<br>GCTCGTGACCGTCAGCGCCGCACCTAACGATTCGCTCCGGCAGGCACTAATGCTCGACCTGCTAAAAGTCTCATCACCGC<br>CAGGCAATGGCGGACCATGGCGACCGCTGGAGGTGACTGTTGTCGCTCGAAGCGAAGTAGTGCCCTGGCGCTATCCGGC<br>GCGACGTGGGCTTCAGTTCGGTGAGTGGCTCCGCCACGACATCCTCTCCGGAACGTTCGAGCCTGCCGTTCTGGATCACG<br>ATCTTGCGATTTTGCTGACCAAGGCGAGGCAACACAGCCTTGCACTGCTAGGTCCATCCGCAGTCACGTTCTTCGAGCCG<br>GTGCCGAACGAGCATTTTTCCAAGGCGCTTTTCGACACGATTGCCCAGTGGAATTCAGAGTCGGATTGGAAGGGTGACGA<br>GCGGAACGTCGTTCTTGCTCTTGCTCGCATTTGGTACAGTGCTTCAACGGGTCTCATTGCTCCTAAGGACGTTGCTGCCGC<br>ATGGGTATCGGAGCGTTTGCCTGCCGAGCATCGGCCCATCATTTGCAAGGCACGCGCGGCGTACCTGGGTAGCGAGGACG<br>ACGACCTAGCAATGCGCGTCGAAGAGACGGCTGCGTTCGTTCGATATGCCAAAGCAACGATTGAGAGAATCTTGCGTTGA<br>GCACGACGTGCGAAAGTGCATCGACCGGCGCCCAGGCACTTGATGCCTAACTCCGCGTTGAAGTGGAGGCTGCGCCCGCC<br>GCTCAACTATGCG
aadA5	TTAGGCATCATGGGTGAATTTTTCCCTGCACAAGTTTTCAAGCAGCTGTCCCACGCTCGCGCGGTGATCGAGCGCCATCTG<br>GCTGCGACACTGGACACAATCCACCTGTTCGGATCTGCGATCGATGGAGGGCTGAAGCCGGACAGCGACATAGACTTGCT<br>CGTGACCGTCAGCGCCGCACCTAACGATTCGCTCCGGCAGGCGCTAATGCTCGATTTGCTGAAAGTCTCATCACCGCCAG<br>GCGATGGCGGAACATGGCGACCGCTGGAGCTAACTGTTGTCGCTCGAAGCGAAGTAGTGCCTTGGCGCTATCCGGCGCGG<br>CGTGAGCTTCAGTTCGGTGAGTGGCTCCGCCACGACATCCTTTCCGGAACGTTCGAGCCTGCCGTTCTGGATCACGATCTT<br>GCGATTTTGCTGACCAAGGCGAGGCAACACAGCCTTGCGCTTCTAGGCCCATCCGCAGCCACGTTTTTCGAGCCGGTGCC<br>GAAGGAGCATTTCTCCAAGGCGCTTTTCGACACTATTGCCCAGTGGAATGCAGAGTCGGATTGGAAGGGTGACGAGCGGA<br>ACGTCGTTCTTGCTCTTGCTCGCATTTGGTACAGCGCTTCAACTGGTCTCATTGCTCCTAAGGACGTTGCTGCCGCATGGGT<br>ATCGGAGCGTTTGCCTGCCGAGCATCGGCCCCTCATCTGCAAGGCACGCGCGGCGTACCTGGGTAGCGAGGACGACGACC<br>TAGCAATGCGCGTCGAAGAGACGGCCGCGTTCGTTCGATATGCCAAAGCAACGATTGAGAGAATCTTGCGTTGAGCGGCA<br>TGTGCGAAAAGTGCATCGACCCGCGCCGAGGGCATCTGATGCCTAACTCGGCGTTCAAGCGGACGGGCTGCGCCCGCCGC<br>TCAACTATGCG
aadA6	TTAGACATCATGAGTAACGCAGTACCCGCCGAGATTTCGGTACAGCTATCACTGGCTCTCAACGCCATCGAGCGTCATCT<br>GGAATCAACGTTGCTGGCCGTGCATTTGTACGGCTCTGCACTGGACGGTGGCCTGAAGCCATACAGTGATATTGATTTGCT<br>GGTTACTGTGGCTGCACGGCTCGATGAGACTGTCCGACAAGCCCTGGTCGTAGATCTCTTGGAAATTTCTGCCTCCCCTGG<br>CCAAAGTGAAGCTCTCCGCGCCTTGGAAGTTACCATCGTCGTGCATGGTGATGTTGTCCCTTGGCGTTATCCGGCCAGACG<br>GGAACTGCAATTCGGGGAGTGGCAGCGTAAGGACATTCTTGCGGGCATCTTCGAGCCCGCCACAACCGATGTTGATCTGG<br>CTATTCTGCTAACTAAAGTAAGGCAGCATAGCCTTGCATTGGCAGGTTCGGCCGCAGAGGATTTCTTTAACCCAGTTCCGG<br>AAGGCGATCTATTCAAGGCATTGAGCGACACTCTGAAACTATGGAATTCGCAGCCGGATTGGGAAGGCGATGAGCGGAA<br>TGTAGTGCTTACCTTGTCTCGCATTTGGTACAGCGCAGCAACCGGCAAGATCGCACCGAAGGATATCGTTGCCAACTGGG<br>CAATGGAGCGTCTGCCAGATCAACATAAGCCCGTACTGCTTGAAGCCCGGCAGGCTTATCTTGGACAAGGAGAAGATTGC<br>TTGGCCTCACGCGCGGATCAGTTGGCGGCGTTCGTTCACTTCGTGAAACATGAAGCCACTAAATTGCTTAGTGCCATGCCA<br>GTGATGTCTAACAATTCATTCAAGCCGACGCCGCTTCGCGGCGCGGCTTAATTCAGGCG
aadA7	TTAGACATCATGAGTGAAAAAGTGCCCGCCGAGATTTCGGTGCAACTATCACAAGCACTCAACGTCATCGGGCGCCACTT<br>GGAGTCGACGTTGCTGGCCGTGCATTTGTACGGCTCCGCACTGGATGGCGGATTGAAACCGTACAGTGATATTGATTTGCT<br>GGTGACTGTAGCTGCACCGCTCAATGATGCCGTGCGGCAAGCCCTGCTCGTCGATCTCTTGGAGGTTTCAGCTTCCCCTGG<br>CCAAAACAAGGCACTCCGCGCCTTGGAAGTGACCATCGTCGTGCACAGTGACATCGTACCTTGGCGTTATCCGGCCAGGC<br>GGGAACTGCAGTTCGGAGAGTGGCAGCGCAAAGACATCCTTGCGGGCATCTTCGAGCCCGCCACAACCGATTCTGACTTG<br>GCGATTCTGCTAACAAAGGCAAAGCAACATAGCGTCGTCTTGGCAGGTTCAGCAGCGAAGGATCTCTTCAGCTCAGTCCC<br>AGAAAGCGATCTATTCAAGGCACTGGCCGATACTCTGAAGCTATGGAACTCGCCGCCAGATTGGGCGGGCGATGAGCGG<br>AATGTAGTGCTTACTTTGTCTCGTATCTGGTACACCGCAGCAACCGGCAAGATCGCGCCAAAGGATGTTGCTGCCACTTGG<br>GCAATGGCACGCTTGCCAGCTCAACATCAGCCCATCCTGTTGAATGCCAAGCGGGCTTATCTTGGGCAAGAAGAAGATTA<br>TTTGCCCGCTCGTGCGGATCAGGTGGCGGCGCTCATTAAATTCGTGAAGTATGAAGCAGTTAAACTGCTTGGTGCCAGCC<br>AATGATGTCTAACAATTCATTCAAGCCGACGCCGCTTCGCGGCGCGGCTTAATTCAAGCG
aadA9	TTAGACATGATGAGCAACTCTATACACACCGGAATCTCAAGACAGCTTTCACAGGCACGCGATGTAATTAAACGCCATTT<br>GGCATCAACGCTGAAAGCCATACACTTGTATGGTTCTGCAATTGATGGTGGCCTCAAACCATATAGCGACATTGATCTGCT<br>GGTTACCGTGGATGCACGCTTGGATGAAGCTACCAGACGCTCCCTGATGCTCGATTTCTTGAATATCTCGGCACCACCATG<br>CGAAAGCTCAATACTCCGGCCGCTAGAGGTAACTGTTGTTGCATGCAACGAAGTAGTGCCTTGGCGTTATCCGGCACGAC<br>GAGAACTGCAGTTCGGGGAGTGGCTGCGGGAGGATATTCTTGAAGGTGTCTTCGAGCCAGCCGCCTTGGACGCCGACCTT<br>GCAATTCTAATAACGAAAGCTAGGCAACACAGCATCGCTTTAGTAGGTCCAGTGGCTCAAAAAGTCTTCATGCCGGTGCC<br>AGAGCATGACTTTCTCCAGGTGCTTTCCGATACCCTTAAGCTGTGGAATACTCATGAGGATTGGGAAAATGAGGAGCGGA<br>ACATCGTACTCACGTTAGCTCGGATCTGGTATAGCACTGAAACTGGAGGAATCGTCCCCAAGGATGTGGCCGCCGAATGG<br>GTTTTAGAGCGCTTGCCAGCTGAGCATAAGCCAATACTGGTTGAGGCGCGGCAAGCCTATCTTGGGCTTTGCAAGGATAG<br>TCTTGCTTTGCGTGCAGATGAGACTTCGGCGTTCATTGGCTATGCAAAGTCTGCGGTCGCTGATTTGCTCGAAAAGCGAAA<br>ATCTCAAACTTCGCATATTTGCGATGGCGCCAAGAACGTCTAACGTCTAACTATTCATTTAAGCCGAAGCCGCTTCGCGGC<br>TCGGCTTAATTCAGGCG
aadA10	TTAGACATCATGATAAACACAGTGCCCGCCGAGATTTCGGTACAGTTATCACAGGCACTCAACGTCATCGAGCATCATCT<br>GGGATCGACGTTGCTGGCCATGCATTTGTATGGCTCTGCACTCGACGGTGGCCTGAAGCCATACAGTGATATTGATTTGCT<br>GGTTACTGTGACCGCACGGCTCGATGAGAGTGTGCGGCAAGCTCTGTTCGTCGATCTCTTGGGGGTTTCCGTTTTCCCTGG<br>TCAAAGCAGAGTTCTCCGCGCCTTGGAAGTTACCATTGTCGTGCACAGTGACATCGTTCCTTGGCGCTATCCGGACAGACG<br>GGAACTGCAATTCGGGGAGTGGCAGCGCAAAGACATTCTTGCGGGCATCTTCGAGCCTGCGACAACCGATGTTGATCTAG<br>CCATTCTGCTAACAAAAGCAAGGCAACATAGCCTTGCCTTGGCCGGTTCGGCTGGGGAAGATTTCTTCAACCCAGTCCCG<br>GAAAGCGATCTATTCAAGGCACTGGCCGACACCCTGAAACTATGGAACTCACAGCCGGATTGGATAGGTGACGAGCGGA<br>ATGTAGTGCTTACTTTGTCTCGTATTTGGTACAGCGCAGCAACCGGCAAGATCGCGCCGAAGGATGTTGCCGCCAACTGG<br>GTAATGGAGCGTTTGCCAGTTCAACATCAGCCCGTGCTGCTTGAAGCCCGGCAGGCTTATCTTGGACAAGGAGAAGATTG<br>CTTGGCTTCGCTCACGGATCAGTTAGAGGCGTTTGTTCACTTCGTGAAGCATGAAGCCACTAAACTGCTTGGTGCCACGCC<br>AATGATGTCTAACAATTCATTCAAGCCGACGCCGCTTCGCGGCGCGGCTTAATTCAGGCG
aadA11	TTAGACATCATGAGGGAAGCGGTGACCGCCGAAATTTCAACACAACTATCAGAGGTGTTTAGCGTCATCGAGCGCCATCT<br>GGAGCCGACGTTGCTTGCCGTGCATTTGTACGGCTCCGCAGTGGATGGCGGCTTGAAGCCATACAGCGATATTGATTTGCT<br>GGTTACTGTGACCGCAAGGCTTAATGAAGCAACACGGCAAGCTTTGCTCAACGACCTTTTGGAGGCTTCGGCTTTCCCTGG<br>CGAGAGCGAGACTCTCCGCGCTATAGAAGTCACCTTTGTCGTGCACGACGACATCATCCCGTGGCGTTATCCAGCTAAGC<br>GCGAACTGCAATTTGGAGAATGGCAGCGTAATGACATTCTTGCGGGTATCTCCGAGCCAGCCGCGATCGACGTTGATCTG<br>GCTATCTTGCTGACAAAAGCAAGAGAACATAGCGTTGCCTTGGTAGGTCCAGCTGCGGAGGAACTCTTTGATCCCGTTCC<br>TGAACAGGATCTATTCGAGGCACTGAATGAAACCTTGAAGCTATGGAACTCGCAGCCCGACTGGGCCGGCGATGAGCGA<br>AATGTAGTGCTCACGTTGTCCCGCATTTGGTACACCGAAGTAACCGGAAAAATCGTGCCGAAGGATGTCGCTGCCGACTG<br>GGCAATGGAGCGCCTACCTGCCCAGCATCAGCCCGTCTTACTTGAAGCTAGACAGGCTTATCTTGGACAAAAAGAAGATC<br>GCTTGGCCTCGCGCGCAGATCAGTTGGAAGAATTTGTTCACTTCGTGAAAGGCGAGATCACTAAGGTAGTCGGCAAATGA<br>TGTCTAACAATTCGTTCAAGCCGATTCCGCTTCGCGGCACGGCTTAACTCAGGCG
aadA13	TTAGACATCATGAGGGACTCAGTGACCGCCGAAATTTCGACGCAACTATCCAAGGTGCTTAGTGTTATCGAGCACCATCT<br>GGAACCGACGTTGCTTGCCGTACATTTGTACGGCTCCGCAGTGGATGGCGGCCTGAAGCCATACAGTGATATTGATTTGCT<br>GGTTACTGTGACCGCAAGGCTTGATGACACAACGCGGCGAGCTTTGTTCAACGATCTTTTGGAGGTTTCGGCTTTCCCAGG<br>CGAGAGTGAGATTCTCCGCGCTATAGAAGTCACCATTGTCGTGCACGAAGACATTATGCCGTGGCGTTATCCAGCCAAGC

	GCGAACTGCAATTTGGAGAATGGCAGCGCAATGACATTCTTGCGGGTATCTTCGAGCCAGCCACGATCGACATCGATCTG<br>GCTATCTTGCTAACGAAAGCGAGAGAACATAGCGTGGCTTTGGTAGGTCCGGCGGCGGAGGAACTCTTTGATCCAGTTCC<br>TGAACAAGATCTAATCAAGGCGCTGAATGAAACCTTGAAGCTATGGAACTCGCAGCCCGACTGGGCCGGCGATGAGCGA<br>AATGTAGTGCTTACGTTGTCCCGCATTTGGTACAGCGCAGCAACTGGTAAAATCGCGCCGAAGGATGTCGCTGCCAACTG<br>GGCAATGGAACATCTACCTGCCCAGCATCAGTCTGTCTTGCTTGAAGCTAGACAGGCTTATCTTGGGCAAGAGGAAGATC<br>GCTCGGTCTTGCGCGCAGATAAGTTGGAAGAATTTATTCACTTCATGAAAAGCGAGATCACCAAGGTGCTCGGCAATGAT<br>GTCTAACAATGCGTTCAAGCCGATGCCGCTTCGCGGCACGACTTAACTTCGGCG
aadA16	TTAGACATCATGAGCAACGCAGTGCCCGCCGAGATTTCGGTACAGCTATCACAGGCACTCAACGTCATCGAGCATCATCT<br>GGGATCGACGTTGCTGGCCGTGCATTTGTACGGCTCTGCACTCGACGGTGGCCTGAAGCCATGCAGTGATATTGATTTGCT<br>GGTTACTGTGACTGCACAGCTCGATGAGACTGTGCGGCAGGCTCTGTTCGTAGATTTCCTGGAAGTTTCCGCTTCTCCCGG<br>CCAAAGTGAAGCTCTCCGTGCCTTGGAAGTTACCATCGTCGTGTACGGCGATGTTGTTCCTTGGCGTTATCCAGCCAGACG<br>GGAACTGCAATTCGGGGAGTGGCAGCGCAAGGACATTCTTGCGGGCATCTTCGAGCCCGCGACAACCGATGTTGATCTGG<br>CTATTCTGCTAACTAAAGCAAGGCAACACAGCCTTGCCTTGGCAGGTTCGGCCGCGGAAGATTTCTTCAACTCAGTCCCG<br>GAAAGCGATCTATTCAAAGCACTGGCCGACACCTTGAAACTATGGAACTCACAACCGGATTGGGCAGGCGACGAGCGGA<br>ATGTAGTGCTTACTTTGTCTCGCATTTGGTACAGCGCAGCAACCGGCAAGATCGCGCCGAAGGATGTAGCTGCCAACTGG<br>GTAATGGAACGCCTGCCCGTCCAACATCAGCCCGTGCTGCTTGAAGCCCAGCAGGCTTACCTTGGACAAGGGATGGATTG<br>CTTGGCCTCACGCGCTGATCAGTTGACTGCGTTCATTTACTTTGTGAAGCACGAAGCCGCCAGTCTGCTCGGCTCCACGCC<br>AATGATGTCTAACAGTTCATTCAAGCCGACGCCGCTTCGCGGCGCAGCTTAATTCAGGCG
aadA24	TTAGACATCATGAGGGACGCAGTGATCGCCGAAATTTCGACACAACTGTTAGAGGTGCTTAGTGTCATTGAGCGCCATCT<br>GGAGCCGACGTTGCTGGCCGTGCATTTGTACGGCTCCGCAGTGAATGGCGGCCTGAAGCCATACAGCGATATTGATTTGC<br>TGGTTACTGTGACTGTAAGGCTTAATGAAACAACGCGGCGAGCTTTGCTCAACGACCTTCTGGAGGTTTCGACTTTCCCCG<br>GCGAGAGTGAGGCTCTCCGCGCTATAGAAGTCACCATTGTCGTGCACGACGACATCATTCCGTGGCGTTATCCAGCTAAG<br>CGAGAACTGCAATTTGGAGAATGGCAGCGCAATGACATTCTTGCGGGTATCTTCGAGCCAGCCACGATCGACATTGATCT<br>GGCTATCTTGCTGACAAAAGCAAGAGAACATAGCGTTGCCTTGGTAGGTCCAGCGGCGGAGGAACTCTTTGATCCGGTCC<br>CTGAACAGGATCTATTCGAGGCACTAAATGAAACCTTGAAGCTATGGAACTCGCAGCCCGACTGGGCTGGCGATGAGCG<br>AAATGTAGTGCTTACGTTGTCTCGTATTTGGTACAGCGCAGTAACCGGCAAAATCGCGCCGAAGGATGTCGCTGCCGACT<br>GGGCAATGGAGCGCCTACCGGCCCAGTATCAGCCCGTCTTGCTTGAAGCTAGACAGGCTTATCTTGGACAAGAAGAAGAT<br>CGCTTGGCCTTGCACGCTGATCAGTTGGAAGAATTTGTTCACTACGTGAAAGGCGAGAGCACCAAGGTAGTCGGCAAATG<br>ATGTCTAACAATTCGTTCAAGCCGACGCCGCTTCGCGGCGCGGCTTAACTCGAGCG
aadA28	TTAGACATCATGAGGGACTCAGTGACCGCCGAAATTTCGACGCAACTATCCAAGGTGCTTAGTGTTATCGAGCACCATCT<br>GGAACCGACGTTGCTTGCCGTACATTTGTACGGCTCCGCAGTGGATGGCGGCCTGAAGCCATACAGTGATATTGATTTGCT<br>GGTTACTGTGACCGCAAGGCTTGATGACACAACGCGGCGAGCTTTGTTCAACGATCTTTTGGAGGTTTCGGCTTTCCCAGG<br>CGAGAGTGAGATTCTCCGCGCTATAGAAGTCACCATTGTCGTGCACGAAGACATTATGCCGTGGCGTTATCCAGCCAAGC<br>GCGAACTGCAATTTGGAGAATGGCAGCGCAATGACATTCTTGCGGGTATCTCCGAGCCAGCCATGATCGACGTTGATCTG<br>GCTATCTTGCTGACAAAAGCAAGAGAACATAGCGTTGCCTTGGTAGGTCCAGCTGCGGAGGAACTCTTTGATCCCGTTCC<br>TGAACAGGATCTATTCGAGGCACTGAATGAAACCTTGAAGCTATGGAACTCGCAGCCCGACTGGGCCGGCGATGAGCGA<br>AATGTAGTGCTTACGTTGTCCCGCATTTGGTACAGCGCAATAACCGGCAAAATCGCGCCGAAGGATGTCGCTGCCGACTG<br>GGCAATAAAACGCCTACCTGCCCAGTATCAGCCCGTCTTACTTGAAGCTAAGCAAGCTTATCTGGGACAAAAAGAAGATC<br>ACTTGGCCTCACGCGCAGATCACTTGGAAGAATTTATTCGCTTTGTGAAAGGCGAGATCATCAAGTCAGTTGGTAAATGA<br>TGTCTAACAATTCGTTCAAGCCGACCGCGCTACGCGCGGCGGCTTAACTCCGGCG
aadA29	TTAGACATCATGAGTAACGCAGTACCCGCCGAGATTTCAGTACAGCTATCACAGGCACTCAACGTCATCAAGCGTCATCT<br>GGAATCAACGTTGCTGGCCGTGCATTTGTACGGCTCCGCACTGGACGGCGGCCTGAAACCATACAGTGATATTGATTTGC<br>TGGTTACTGTGGACACACGGCTCGATGAGACCGTAAGGCAAGCTCTGTTCGTCGATCTGTTGGAGATTTCGGCTTTCCCTG<br>GCCAAAGCAAAGTGCTCCGCGCCTTGGAAGTCACCATCGTCGTGTACAGCGACGTCGTCCCCTGGCGTTATCCGGCCAGA<br>CGGGAACTACAGTTCGGAGAGTGGCAGCGCAAAGACATTCTTGCGGGTATCTTCGAGTCCGCCACAAATGATGTCGATCT<br>GGCGATCCTACTAACAAAAGCAAGGCAACATAACATCGCATTGGTGGGTTCGGCTGCGGAGGATTTCTTCAACCCAGTTC<br>CGGAAAGCGATCTATTCAAGGCACTGGCCGGCACCCTGAAACTATGGAACTCGCAGCCGGATTGGGAAGGTGACGAGCG<br>TAATGTAGTGCTTACTTTGTCTCGTATTTGGTACAGCGCAGCAACTGGTAAGATCGCGTCCAAGGATGTCGCTTCTAACTG<br>GGCAATGGAGCGTCTGCCAGCTCAACATCAGCTTGTGCTATTTGAAGCCCGGCAGGCTTATCTTGGAGATGGAGGAGATT<br>GCTTGGCTTCGCGCGCAGATCAGTTGACGGCGTTCGTTCACTTTGTGAAGTATGAAGCCGGCAGACTCCTTGGCTCCACAC<br>CAATGATGTCTAACAGTACATTCAAGCCGACGCCGCTTCGCGGCGCGGCTTAATTCAAGCG
aadA34	TTAGACATCATGAGTAACGCAGTACCCGCCGAGATTTCGGTACAGCTATCACTGGCTCTCAACGCCATCGAGCGTCATCT<br>GGAATCAACGTTGCTGGCCGTGCATTTGTACGGCTCTGCACTGGACGGTGGCCTGAAGCCATACAGTGATATTGATTTGCT<br>GGTTACTGTGGCTGCACGGCTCGATGAGACTGTCCGACAAGCCTTGGTCGTAGATCTCTTGGAAATTTCTGCCTCCCCTGG<br>CCAAAGTGAAGCTCTCCGCGCCTTGGAAGTTACCATCGTCGTGCATGGTGATGTTGTCCCTTGGCGTTATCCGGCCAGACG<br>GGAACTGCAATTTGGAGAATGGCAGCGCAATGACATTCTTGCAGGTATCTTCGAGCCAGCCACGATCGACATTGATCTGG<br>CTATCTTGCTGACAAAAGCAAGAGAACATAGCGTTGCCTTGGTAGGTCCAGCGGCGGAGGAACTCTTTGATCCGGTTCCT<br>GAACAGGATCTATTTGAGGCGCTAAATGAAACCTTAACGCTATGGAACTCGCCGCCCGACTGGGCTGGCGATGAGCGAA<br>ATGTAGTGCTTACGTTGTCCCGCATTTGGTACAGCGCAGTAACCGGCAAAATCGCGCCGAAGGATGTCGCTGCCGACTGG<br>GCAATGGAGCGCCTGCCGGCCCAGTATCAGCCCGTCATACTTGAAGCTAGACAGGCTTATCTTGGACAAGAAGAAGATCG<br>CTTGGCCTCCCGCGCAGATCAGTTGGAAGAATTTGTTCACTACGTGAAAGGCGAGATCACCAAGGTAGTCGGCAAATAAT<br>GTCTAACAATTCGTTCAAGCCGACGCCGCTTCGCGGCGCGGCTTAACTCAAGCG
aadB<br>*ANT(2'')-Ia*	TTAGGCCGCATGGACACAACGCAGGTCACATTGATACACAAAATTCTAGCTGCGGCAGATGAGCGAAATCTGCCGCTCTG<br>GATCGGTGGGGGCTGGGCGATCGATGCACGGCTAGGGCGTGTAACACGCAAGCACGATGATATTGATCTGACGTTTCCCG<br>GCGAGAGGCGCGGCGAGCTCGAGGCAATAGTTGAAATGCTCGGCGGGCGCGTCATGGAGGAGTTGGACTATGGATTCTT<br>AGCGGAGATCGGGGATGAGTTACTTGACTGCGAACCTGCTTGGTGGGCAGACGAAGCGTATGAAATCGCGGAGGCTCCG<br>CAGGGCTCGTGCCCAGAGGCGGCTGAGGGCGTCATCGCCGGGCGGCCAGTCCGTTGTAACAGCTGGGAGGCGATCATCT<br>GGGATTACTTTTACTATGCCGATGAAGTACCACCAGTGGACTGGCCTACAAAGCACATAGAGTCCTACAGGCTCGCATGC<br>ACCTCACTCGGGGCGGAAAAGGTTGAGGTCTTGCGTGCCGCTTTCAGGTCGCGATATGCGGCCTAACAATTCGTCCAAGC<br>CGACGCCGCTTCGCGGCGCGGCTTAACTCAGGTG
aphA15<br>*APH(3')-XV*	TTAGACCGCTATGACAGTCGCCCTCGACGAAGTATCTGAACTAAAGAATTTGCTTTCACCCTTGTTGGATGAATGCACTTT<br>TGAAGAAGTTGAGTATGGTCAGTCAGATGCTCGAGTGATTCGAGTTCTATTTCCTGATCGCAATACCGCGTATCTAAAGTA<br>CGCCTCCGGATCTTCTGCTCAAGAAATTCTTCAAGAGCATCAGCGCACTAGATGGCTCAGAACACGAGCTCTCGTACCGG<br>AAGTGATCTCATATGTCTCGACTTCAACTGTCACCATCCTGTTGACAAAAGCATTGATTGGCCACAATGCCGCTGACGCCG<br>CAGATGCAGATCCAGTTATTGTTGTTGCAGAGATGGCACGAGCGTTACGCGACCTCCATTCGATCTCGCCTGACGATTGCC<br>CATTCGACGAAAGGCTCCACCTGCGACTGAAGCTGGCTTCGGGCCGTTTGGAAGCCGGGTTAGTTGATGAGGAGGACTTT<br>GATCACGCAAGGCAAGGCATGCTGGCGCGGGATGTTTACGAGCAACTTTTTATACAAATGCCTGGAGCGGAGCAGCTGGT<br>AGTCACACATGGCGACGCCTGTCCCGAGAACTTCATCTTCCAAGGTAATGCCTTCGTCGGCTTCATAGACTGCGGTCGGGT<br>CGGGCTTGCCGATAAGTATCAAGACCTGGCGCTTGCATCGAGAAACATTGACGCGGTATTTGGACCAGAACTCACTAACC<br>AGTTCTTCATCGAGTATGGAGAGCCAAATCCGAACATAGCTAAGATTGAGTACTACCGGATCTTGGATGAGTTCTTCTAA<br>GCGCGGTCTAACAATTCGTTCAAGCCGAGATCGCTTCGCGGCCGCGGAGTTGTTCTGTAAATTGTCACAACGCCGCGGCC<br>GCAAAGCGCTCCGGCTTAACTCAGGCG
aphA16	TTAGCTTGACGCTCCGCGCAGGAAAGAGAAAATGGATAAACTTCCAAATTTCATATTTGAAAACTATAGTGACTTAAAAA<br>TTGAACGAGATACTGAGGGTTGGTCTCCGGCTGAGGTATATAGTGTTACAACCAAGAAAAAAAGGTGGTTTCTAAAAAGA<br>AGTCATACGAGATACAATAAAACTACATATAATGTAAGAAGAGAGAAAGAAATTATTGAATGGGCCTATCCAAAGTTTA<br>GAGTACCACAAATAATACATTATGAAGAAGCAAAAGAATATAATTCATTACTAATGAATCACATTGGCGGCTCTAGCCTT

	GAAATGCTTGGTCCATCAATTACTTTAGAGAAATATATCGATTACTATGTACAATCCTTAAAATTAATGCAGTCCATTAAC<br>ATCGAAAACTGTCCATACAATAATTGTATAAAGAATAGAATAATCGAATTAGAATATTTATTAGAGAATGATCTAGCAGA<br>TATTAATTCAAATAACTGGGAAGAAGATACGCGTGAGCATTTCAGAAACGGTAAAGACTTATTTAATTATATAGTAAATA<br>ATAAGCCAAATGAAGACTTAGTTTTCTCACATGGAGATATGACAAACTCTAACATATTTATTGAAAACGAAGAAGTTGGA<br>TTTATAGATCTCGGTCGATGTGGATTAGCTGATAAGTGGGTTGATATTGCATTTTGTGTTAGAGATATAAGAGAAATAAGT<br>AATGAAAATAAATGGATAAAGATGTTATTTGATAAATTAGAAATAGTACCCAATTGGGATAAAATGAGATACTACATTTT<br>GTTAGATGAGCTGTTCTAAGAATAAGCTAACAACCGCTTCAACCTGGCAACGCTACTGTCACGGTTTATGCTACTCGCTTC<br>GCTCGCTTACGCATAAACCGCGCCAGTTTACGCGTTGCAGGTTAAGCGAATG
sat2	TTAGGCGTCATATGAAGATTTCGGTGATCCCTGAGCAGGTGGCGGAAACATTGGATGCTGAGAACCATTTCATTGTTCGT<br>GAAGTGTTCGATGTGCACCTATCCGACCAAGGCTTTGAACTATCTACCAGAAGTGTGAGCCCCTACCGGAAGGATTACAT<br>CTCGGATGATGACTCTGATGAAGACTCTGCTTGCTATGGCGCATTCATCGACCAAGAGCTTGTCGGGAAGATTGAACTCA<br>ACTCAACATGGAACGATCTAGCCTCTATCGAACACATTGTTGTGTCGCACACGCACCGAGGCAAAGGAGTCGCGCACAGT<br>CTCATCGAATTTGCGAAAAAGTGGGCACTAAGCAGACAGCTCCTTGGCATACGATTAGAGACACAAACGAACAATGTAC<br>CTGCCTGCAATTTGTACGCAAAATGTGGCTTTACTCTCGGCGGCATTGACCTGTTCACGTATAAAACTAGACCTCAAGTCT<br>CGAACGAAACAGCGATGTACTGGTACTGGTTCTCGGGAGCACAGGATGACGCCTAACAATTCATTCAAGCCGACACCGCT<br>TCGCGGCGCGGCTTAATTCAGGAG
arr2	TTATGCAGCCAAATCCCAACAATTAAGGGTCTTAAAATGGTAAAAGATTGGATTCCCATCTCTCATGATAATTACAAGCA<br>GGTGCAAGGACCGTTCTATCATGGAACCAAAGCCAATTTGGCGATTGGTGACTTGCTAACCACAGGGTTCATCTCTCATTT<br>CGAGGACGGTCGTATTCTTAAGCACATCTACTTTTCAGCCTTGATGGAGCCAGCAGTTTGGGGAGCTGAACTTGCTATGTC<br>ACTGTCTGGCCTCGAGGGTCGCGGCTACATATACATAGTTGAGCCAACAGGACCGTTCGAAGACGATCCGAATCTTACGA<br>ACAAAAAATTTCCCGGTAATCCAACACAGTCCTATAGAACCTGCGAACCCTTGAGAATTGTTGGCGTTGTTGAAGACTGG<br>GAGGGGCATCCTGTTGAATTAATAAGGGGAATGTTGGATTCGTTAGAGGACTTAAAGCGCCGTGGTTTACACGTCATTGA<br>AGACTAGTCCTTTGCATAACAAAGCCATCAAACCGGACGCCAGAGATTCCGCGCCTGTTGCGCATGGCTTCGCCATTTTAT<br>GCGCAATAGGCGCGCCACCCTGTCGCCGTTTATGGCGGCG
arr5	TTAGGCAACAAACCAATCTCTTTCGCTACATGGAACAAACGATGACGGTAGACTGGATCCCCATTTCGCACGACAACTAC<br>CATCAAGTGCGTGGCCCGTTTTATCACGGAACAAAAGCCGAACTCGCCATTGGCGACTTAATTTCAACCGGATTTATTTCT<br>CACTTTGAGCGGGACAGAGCACTAAAGCATGTGTACTTTTCCGCGCTGATGGAGCCAGCAATCTGGGGGGCCGAGCTCGC<br>TGTAGCACTCTCTGGCTCTGACGGGCCAGGCCATATTTACATCATTGAGCCAACCGGCCCGTTTGAAGACGACCCCAATCT<br>CACAAACAAACGATTCCCTGGCAATCCAACACAGTCCTATCGCACATGCCACCCACTTAAAATTGTTGGCATACTGCGGG<br>AGTGGGAGCGCCATTCTCCTGAAGCATTGAAGACCATGCTAGATTCTCTGGCAGACCTCAAGCGACGCGGCTTGGCCATC<br>ATTGAAGAATGAAATTGTTGCCTAACAATTCATTCAAGCCGACGCCGCTTCGCGGCGCGGCTTAATTCAGGCG
arr6	TTAGGCAGCAACCCCATTCTCGCCCCATGGAACCTAAGAAATGTCGAGTGACTGGACTCCCATCTCACATGAGAATTGCC<br>AGCAGGTGCGTGGGCCGTTCTATCACGGCACCAAAGCCCATCTATCGATTGGCGACTTGATAACAACTGGGCATCTCTCC<br>CACTTTGAAGATGGACGCGCTCTTAAACACGTCTACTTTTCAGCTTTGATGGAGCCTGCCATTTGGGGGGCGGAACTTGCA<br>ATGTCGTTGTCACGCCTAGATGGCCGTGGCTACATATACATCGTCGAACCAACTGGACCGTTTGAGGACGACCCGAATCT<br>TACGAACAAAAGATTTCCTGGAAATCCAACAAAGTCCTATCGCACGTGCGATCCGCTACGAATTGTCGGGTCAGTCGAAG<br>ACTGGCAAGGGCATCCCGCTGATGTGCTGCAACAGATGTTGGAGTCTTTAGAGGACCTAAAGCGCCGTGGTCTTGCCATC<br>ATCGAGGATTAGAACTGCTGCCTAACAATTCATTCAAGCCGACGCCCCTTCGGGGCGCGGCTTAATTCAGGCG
arr7	TTAGGCAGCAAACCACCTTCCTCGCTAGACGGAACCAACGAATGCCGAATGACTGGATTCCCACCTCGCACGAAAACTGC<br>TCGCTCGTGCCGGGGCCGTTCTACCACGGCACCAAAGCAAAACTCGCAATAGGTGACTTGCTTTCGCCTGGACACCCGTC<br>TCACTTTGAGCAAGGCCGTAGGCTCAAACACATCTATTTTGCCGCACTGATGGAGCCAGCCATCTGGGGTGCTGAGCTTG<br>CAATGTCATTGTCACGCCAAGAGGGGCGCGGTTACATTTACATTGTTGAACCGCTCGGGCCGTTTGAGGACGACCCAAAC<br>CTTACAAACAAAAAATTTCCGGGCAATCCAACCAAGTCCTACCGCACCAGTGAGTCGCTACGGATTGTGGAGGTAGTAGA<br>GGACTGGCAAGGCCACTCACCGGATGTGCTGCAGGGCATGTTGGCATCACTGGAGGATCTTCAGCGTCGCGGCCTCGCAA<br>TCATTGAGGACTAGAAATTGCTGCCTAACAATTCGTTCAAGCCGAACTTGCTTCGTTCCACCAAAGCCATGGCAGATTAA<br>GCTTGCCATGGCTTTGGCTCCACTACGCAAGTCGGCTTAACTCAGGCG
arr8b	TTAGGCTGCCAAACCTTTTTCCAACCTTCGGAGTGAAGAATGATGAAAGATTGGGTTCCAATCACGCATGCAAATTGTAA<br>AGATATGCAGGGACCGTTTTATCATGGTACCAAAGCTAAATTATCGGTAGGTGAACTCCTAACAACTGGGTTCAACACTC<br>ATTTTGAAGAGGGTCGCACACTCAAACACGTTTATTTTTCAGCTATGCTTGAGCCAGCAATTTGGGGCGCTGAACTTGCTG<br>TTTCACTGTCAGGTCTAGATGGCCGGGGATACATATACTTAGTTGAACCAACTGGACCTTTTGAGGATGACCCTAATCTTA<br>CCAATAAGAAATTTCCAGGGAATCCAACAATGTCCTATCGAACTTCCGAACCCCTCCAGATTGTTGGGCTCGTTGAAGAG<br>TGGGAGGGACACTCTGCTGAAGCCCTGAAAACGATGCTGGATTCCTTGGAGAATCTAAAGCGCAATGGTCTTCATGTCAT<br>ATATGATTAAGCGGAGCCTGCCTAACAAATCGTTCCAGCAACGTGCGCTTCGCGCACTCGACAGTCTGTAAGTCGCCTTTT<br>TGTGGTTTTGCTACGCAAAAGTATTCCACAAAAAACCAACTTACAGACTGCGGCTGAACTTAGCG
catB2	TTAGGCGACGCGTGGAGTCGCTCTAGAATTTTCGGGTACAAATTTTATGACGAATTATTTTGAGAGTCCCTTCAAAGGGAA<br>GCTTCTGACTGAGCAGGTGAAGAATCCGAACATCAAGGTAGGGCGGTATAGCTACTATTCCGGCTATTACCATGGGCACT<br>CGTTTGATGATTGTGCTCGCTACCTTCTACCAGACCGTGATGACGTTGATCAGCTGATTATCGGCAGCTTCTGCTCCATCG<br>GATCAGGCGCAGCTTTTATTATGGCTGGGAATCAAGGCCACCGATATGATTGGGTCTCTTCTTTCCCTTTCTTCTACATGA<br>ACGAGGAGCCCGCGTTTGCAAAATCAGTCGATGCATTCCAGCGGGCTGGCGACACAGTTATAGGAAGTGATGTGTGGATC<br>GGTTCGGAGGCCATGATCATGCCCGGGATCAAGATCGGGCATGGAGCGGTGATAGGTAGCCGCGCTTTGGTTGCCAAAG<br>ACGTGGAACCCTACACCATAGTGGGGGGAAACCCTGCAAAGTCGATTAGGAAGCGCTTTTCTGAAGAAGAAATTTCTATG<br>CTTTTAGATATGGCTTGGTGGGATTGGCCGCTGGAACAAATCAAGGAAGCAATGCCTTTTCTTTGTTCGTCTGGCATTGCC<br>AGCCTGTATCGTCGCTGGCAAGGCACAAGCGCCTAACAATACGCTACACACGGACAAATTACTCGCTGCGCTCCTAATTT<br>GCCGGTGAGCGTGGCG
catB3	TTAGACGGCAAAGTCACAGACCGCGGGATCTCTTATGACCAACTACTTTGATAGCCCCTTCAAAGGCAAGCTGCTTTCTG<br>AGCAAGTGAAGAACCCCAATATCAAAGTTGGGCGGTACAGCTATTACTCTGGCTACTATCATGGGCACTCATTCGATGAC<br>TGCGCACGGTATCTGTTTCCGGACCGTGATGACGTTGATAAGTTGATCATCGGTAGTTTCTGCTCTATCGGGAGTGGGGCT<br>TCCTTTATCATGGCTGGCAATCAGGGGCATCGGTACGACTGGGCATCATCTTTCCCGTTCTTTTATATGCAGGAAGAACCT<br>GCATTCTCAAGCGCACTCGATGCCTTCCAAAAAGCAGGTAATACTGTCATTGGCAATGACGTTTGGATCGGCTCTGAGGC<br>AATGGTCATGCCCGGAATCAAGATCGGGCACGGTGCGGTGATAGGCAGCCGCTCGTTGGTGACAAAAGATGTGGAGCCT<br>TACGCTATCGTTGGCGGCAATCCCGCTAAGAAGATTAAGAAACGCTTCACCGATGAGGAAATTTCATTGCTTCTGGAGAT<br>GGAGTGGTGGAATTGGTCACTGGAGAAGATCAAAGCGGCAATGCCCATGCTGTGCTCGTCTAATATTGTTGGCCTGCACA<br>AGTATTGGCTCGAGTTTGCCGTCTAACAATTCAATCAAGCCGATGCCGCTTCGCGGCACGGCTTATTTCAGGCG
catB5	TTAGACGGCAAGAAAAGGTTCCACGAACTCTGATGAAAAACTACTTTGACAGCCCTTTCAAAGGGGAGCTTCTTTCTGAG<br>CAAGTGAAAAATCCAAACATCAAAGTAGGCCGTTATAGCTATTACTCTGGCTACTATCACGGCCACTCATTTGATGAATG<br>CGCGCGATACTTGCATCCAGATCGTGATGACGTTGATAAATTGATCATTGGCAGCTTTTGTTCTATAGGAAGCGGGGCTTC<br>CTTCATCATGGCTGGCAATCAGGGGCATCGGCATGACTGGGCATCATCCTTCCCCTTCTTCTATATGCAAGAGGAACCTGC<br>TTTCTCAAGCGCACTCGATGCCTTCCAAAGAGCAGGTGATACCGCCATTGGCAATGATGTCTGGATAGGCTCGGAGGCAA<br>TGATTATGCCCGGAATCAAAATTGGAGACGGTGCCGTGATAGGTAGTCGCTCGTTGGTGACAAAAGATGTAGTGCCTTAT<br>GCCATCATCGGAGGAAGTCCCGCAAAGCAAATTAAGAAGCGCTTCTCCGATGAGGAAATCTCATTGCTCATGGAGATGGA<br>GTGGTGGAACTGGCCACTGGATAAAATTAAGACAGCAATGCCTCTGCTGTGCTCGTCAAATATTTTTGGTCTGCATAAGTA<br>TTGGCGCGAGTTTGTCGTCTAACAATTCATTCAAGCCGACGCCGCTTCGCGGCACGGCTTAATTCTGGCG
catB6	TTAGACGGCAGAATAAATTTTTGCGATCTCTTATGGAAAATTACTTTGACAGTCCCTTCAAAGGGAAACTACTTTCAGAGC<br>AAGTGACTAACCGCAACATCAAAGTTGGTCGGTACAGCTACTACTCTGGTTACTATCACGGGCATTCATTTGATGACTGCG<br>CACGATACTTGCTCCCAGACCGTGATGACGTTGACAAACTAATCATCGGCAGCTTTTGCTCCATCGGAAGCGGGGCTTCTT<br>TCATCATGGCGGGCAATCAGGGTCACCGGCATGACTGGGTAACATCTTTCCCTTTCTTCTACATGCAAGAAGAGCCAGCTT

	TTTCAAGTTCAACGGACGCCTTTCAAAAGGCCGGTGACACCATCGTCGGCAATGATGTCTGGATAGGATCAGAGGCAATG<br>ATTATGCCCGGCATCAAGATTGGAGATGGCGCGGTAATAGGCAGCCGATCGTTGGTGACGAGAGATGTAGAACCCTATAC<br>CATCATTGGCGGAAACCCTGCAAAGCAAATTAAAAAGCGATTCTCTGACGAGGAGATTTCATTACTCATGGAAATGGAGT<br>GGTGGAACTGGCCGTTAGATAAAATCAAAACAGCTATGCCCCTTCTCTGCTCTTCAGACATTTTTGGTCTGCACAGGCATT<br>GGCGTGGGATTGCCGTCTAACAAGCGCCAGCACCATCGCCTGCGGCGCTGGACTCGCAACAAGTTGCTCGCCCGTGTGGC<br>GGGCG
catB10	TTAGACGGCAAGAAATACGCTCCGTGAAAATCCCATGACCAACTATTTTGAAAGTCCATTTAAAGGCAAACTGCTGGCCG<br>ACCAGGTAAAGAACCCGAACATCAAAGTCGGACGGTATAGCTATTATTCCGGCTATTACCATGGCCATTCGTTTGACGAG<br>TGCGCTCGCTTTCTCTTGCCAGATCGCGATGACATCGACCAACTGATCGTTGGTAGCTTCTGTTCCATCGGCACGGGCGCC<br>TCCTTCATCATGGCCGGAAATCAGGGGCACCGTTATGACTGGGCGTCTTCTTTTCCCTTCTTCTACATGAAAGAGGAGCCA<br>GCATTCTCGGGCGCACTTGATGCATTCCAAAAAGCCGGTGACACAGTCATCGGAAGTGATGTCTGGATAGGCTCTGAGGC<br>CATGATCATGCCCGGCATCAACGTCGGTCATGGCGCTGTGATTGGAAGCCCGCGCTTTGGTCACGAAAGATGTGGAGCCGT<br>ACACTATCGTTGGCGGAAATCCCGCCAAACCGATCAAGAAACGCTTCTCCGACGAGGAGATCGCCATGCTTTTGAAAATG<br>AATTGGTGGGATTGGCCAACTGAAAAAATTGAGGAAGCAATGCCTTTGCTATGCTCATCCAACATCGTTGGGCTGCATCG<br>ATACTGGCAAGGCTTTGCCGTCTAACAATTTATTCAAGCCGACTCCGCTTCGCGGCGCGGCTTAATTCAGGCG
catB11	TTAGGCAACGGTGTTGTTGCAGATTTTTGGTGTGGAATTATATGAAGAACTATTTTGAGAGCCCGTTTAAGGGAAAACCTC<br>TCGTCGAACAGGTAAAGAACCCAAATATCAAGGTGGGCCGTTATAGCTATTATTCAGGCTATTACCACGGGCATTCATTT<br>GATGATTGCGCTCGCTACCTCCTGCCTGATCGTGATGACGTTGATAAATTGATAATTGGAAGCTTTTGCTCCATAGGGACG<br>GGTGCATCTTTTATCATGGCTGGAAATCAAGGTCACCGATATGATTGGGTTACATCATTCCCTTTTTTCTATATGAATGAG<br>GAACCGGCATTTTCGGAATCAGTTGATGCTTTCCAGGCGGCAGGTAATACCGTCATAGGAAGCGACGTGNGGATTGGCTC<br>TGAAGCAATGATTATGCCTGGAGTAAAGGTTGGCCATGGAGCGGTAATTGGCAGCCGGGCTTTGGTTACCAAAGATATAG<br>AGCCATACACAATTGTTGGTGGCAACCCTGCAAAAGAGATAAAGAAGCGCTTTCAGAACAGGAAATTTCAATGTTGCTA<br>GATATGAAGTGGTGGGATTGGCCGTTGGAGCAAATTAAAGAAGCAATGCCTCTTTTGTGCTCGTCTGATATTGCAGGCTT<br>GTACCATTTTTGGCAGCGTTCAAGTGCCTAACAAACGGCTGTTGTCGCCCCTTCGGGGCTGGGACGGCCTTTCCGCCGCTT<br>TGCGGCTACAAGTCCGCCCCAAAGCCGGGCG
cmlA1	TTGGGCGCACAATAAGGCTCCTTGCAGAGTTGCTTGAAAGTTGTTACGATTCAAATTCAATCATGAGATAGTCAGCAGAT<br>GAGCACTTCCAAGAACGCAGACAAGTAAGCCGCAGCAACCTTCATTTTTCGGTTGTTGCGGCGTTCTCATGAATCCTTTTG<br>CTCTACGGGAGCGCCGCCAAATCCTTCGTTCAAGGAGATGGTTTCGTGAGCTCAAAAAACTTTAGTTGGCGGTACTCCCTT<br>GCCGCCACGGTGTTGTTGTTATCACCGTTCGATTTATTGGCATCACTCGGCATGGACATGTACTTGCCAGCAGTGCCGTTT<br>ATGCCAAACGCGCTTGGTACGACAGCGAGCACAATTCAGCTTACGCTGACAACGTACTTGGTCATGATTGGTGCCGGTCA<br>GCTCTTGTTTGGACCGCTATCGGACCGACTGGGGCGCCGCCCCGTTCTACTGGGAGGTGGCCTCGCCTACGTTGTGGCGTC<br>AATGGGCCTCGCTCTTACGTCATCGGCTGAAGTCTTTCTGGGGCTTCGGATTCTTCAGGCTTGTGGTGCCTCGGCGTGCCTT<br>GTTTCCACATTTGCAACAGTACGTGACATTTACGCAGGTCGCGAGGAAAGTAATGTCATTTACGGCATACTCGGATCCAT<br>GCTGGCCATGGTCCCGGCGGTAGGCCCATTGCTCGGAGCGCTCGTCGACATGTGGCTTGGGTGGCGGGCTATCTTTGCGTT<br>TCTAGGTTTGGGCATGATCGCTGCATCTGCAGCAGCGTGGCGATTCTGGCCTGAAACCCGGGTGCAACGAGTTGCGGGCT<br>TGCAATGGTCGCAGCTGCTACTCCCCGTTAAGTGCCTGAACTTCTGGTTGTACACGTTGTGTTACGCCGCTGGAATGGGTA<br>GCTTCTTCGTCTTTTTCTCCATTGCGCCCGGACTAATGATGGGCAGGCAAGGTGTGTCTCAGCTTGGCTTCAGCCTGCTGTT<br>CGCCACAGTGGCAATTGCCATGGTGTTTACGGCTCGTTTTATGGGGCGTGTGATACCCAAGTGGGGCAGCCCAAGTGTCTT<br>GCGAATGGGAATGGGATGCCTGATAGCTGGAGCAGTATTGCTTGCCATCACCGAAATATGGGCTTTGCAGTCCGTGTTAG<br>GCTTTATTGCTCCAATGTGGCTAGTGGGTATTGGTGTCGCCACAGCGGTATCTGTGGCGCCCAATGGCGCTCTTCGAGGAT<br>TCGACCATGTTGCTGGAACGGTCACGGCAGTCTACTTCTGCTTGGGCGGTGTACTGCTAGGAAGCATCGGAACGTTGATC<br>ATTTCGCTGTTGCCGCGCAACACGGCTTGGCCGGTTGTCGTGTACTGTTTGACCCTTGCAACAGTCGTGCTCGGTCTGTCTT<br>GTGTTTCCCGAGTGAAGGGCTCTCGCGGCCAGGGGGAGCATGATGTGGTCGCGCTACAAAGTGCGGAAAGTACATCAAA<br>TCCCAATCGTTGAGAGAATGTGGCAAGCTATCGCCCAACAAATCGCTGCAGCCGACCCAAAACCGCTACGCGGTTTCGGT<br>CGGCTGAGCTCAGGCG
cmlA2	TTGGGCGAACAAAAAGATTCTTCGCGGAATTGCTTGAGAATTGTTACGATTCAAATTCAGCCATGAGATAGTCAGCAAAT<br>GAGCACTTCCAAGAAAGCAGACAAGTAAGCCGCAGCAACCTTCATTTTTCGGTTGTTGCGGCGTTCTCATGAACCCTTTTG<br>CTCTACGGGAGCGCCGCCAAATCCTTCGTTCAAGGAGATGGTTTCGTGCGCTCAAAGAACTGTAATTGGCGGTATTCCCTT<br>GCCGTCACTGTGTTGTTGTTATCACCTTTCGATTTACTGGCATCACTCGGCATGGACATGTACTTGCCAGCGGTGCCTTTCA<br>TGCCACATGCTCTTGGTACGACAGCGGGCACAATTCAGCTTACGCTGACAACGTATTTGGTCATGATAGGGGCCGGTCAG<br>CTTTTGTTTGGGCCACTGTCGGACCGGCTGGGACGTCGTCCCGTGCTACTGGCGGGCGGTGCCGCCTACGTTGCGGCCTCA<br>ATCGGCCTCGTCGTCACGTCATCGGCTGGAGTATTTCTGGGTTTTCGGATTCTCCAAGCTTGTGGTGCCTCGGCATGCCTTG<br>TTGCCACATTTGCAACAGTGCGTGATATCTACGCAGGTCGCAAGGAAAGTAACGTCATCTACGGCTTGCTTGGCTCTATGC<br>TTGCTATGGTTCCGGCGATAGGCCCATTGCTGGGAGCGGTCATAGACACCTGGTTCGGGTGGCGGGCGATCTTTGCGTTCT<br>TGGGATTGGGAATGATCGCTGCATTGACAGCAGCGTGGCGGCTCTGGCCTGAGACCCGGGTGCAGCGACCAGCAGCTTTG<br>CAATGGTCACAACTTCTGCTTCCCATCAAGCACCTTAACTTCTGGTTGTACACAGTGTGTTATGCCGCAGGAATGGGCAGC<br>TTCTTCGTCTTCTTCTCCATAGCGCCCGGATTGATGATGGGTAGGCAAGGCATGTCCCAGTTTGGCTTCAGTCTGTTGTTCG<br>CAACAGTGGCAATTGCGATGATGCTTGCGGCCCGCTTCATGGGGCGCGTAATCGCCAAGTGGGGCAGCCTGAGTGCCTTG<br>CGAATGGGGATGGGCTGCCTGATAGCAGGCGCAGTCTTGCTTGTCATCACCGAGCTATGGATTCCGCAGTCCGTGTTGGG<br>CTTTATTGCCCCAATGTGGCTAGTGGGCGTCGGCGTCGCGACAGCGGTATCCGTTGCACCCAATGGTGCGCTTCGAGGGTT<br>CGACCATATTGCAGGAGCCGTTACGGCAGTCTACTTCTGCTTGGGGGGGCTGCTGCTGGGGAGTGTTGGAACGCTCATCA<br>TTTCGCTGTTGCCGCGCGACACGGCCTGGCCAGTTATCGCGTATTGTTTGGTTCTTGCAACAATCGTGCTTGGACTGTCGT<br>GTGTTTCCCGAGCGAGAGACCTTCGCGGTCACGGGGAGTATGATGCGGTTGCACGCACATAGTGCGGAAAACACACCAA<br>ATCCCGATCACTGGGAATATGGCAAGTTGTCGCCCAACAAATCGCTGCAGCCGACCCAAAACCGCTACGCGGTTTCGGTC<br>GGCTGAGCTCAAGCG
ereA1	TTATGCTCTGTGAGCCGGGTTATTGGCGAAGCGAACGTATGACGATTACAGCAATAAACGCAAAGGTAAAAAAATGACA<br>TGGAGAACGACCAGAACACTTTTACAGCCTCAAAAGCTGGACTTCAATGAGTTTGAGATTCTTACTTCCGTAATTGAGGG<br>CGCCCGAATTGTCGGCATTGGCGAGGGCGCTCATTTTGTCGCGGAGTTTTCACTGGCTAGAGCTAGTCTTATCCGCTATTT<br>GGTCGAAAGGCATGAGTTTAATGCGATTGGTTTGGAATGTGGGGCGATTCAGGCATCCCGGTTATCTGAATGGCTCAACT<br>CAACAGCCGGTGCTCATGAACTTGAGCGATTTTCGGATACCCTGACCTTTTCTGTGTATGGCTCAGTGCTGATCTGGCTGA<br>AATCATATCTCCGCGAATCAGGAAGAAAACTGCAGTTAGTCGGAATCGACTTACCCAACACCCTGAACCCAAGGGACGA<br>CCTAGCGCAATTGGCCGAAATTATCCAGCTCATCGATCACCTCATGAAACCGCACGTTGATATGTTGACTCACTTGTTGGC<br>GTCCATTGATGGCCAGTCGGCGGTTATTTCATCGGCAAAATGGGGGGAGCTAGAAACGGCTCGGCAGGAGAAAGCTATCT<br>CAGGGGTAACCAGATTGAAGCTCCGCTTGGCGTCGCTTGCCCCCGTCCTGAAAAAACACGTCAACAGCGATTTGTTCCGA<br>AAAGCCTCTGATCGAATAGAGTCGATAGAGTATACGTTGGAAACCTTGCGTATAATGAAAACTTTCTTCGATGGTACCTCT<br>CTTGAGGGAGATACTTCCGTACGTGACTCGTATATGGCGGGCGTAGTAGATGGAATGGTTCGAGCGAATCCGGATGTGAA<br>GATAATTCTGCTGGCGCACAACAATCATCTACAAAAAACTCCAGTCTCCTTTTCAGGCGAGCTTACGGCTGTTCCCATGGG<br>GCAGCACCTCGCAGAGAGGGTGAATTACCGTGCGATTGCATTCACCCATCTTGGACCCACCGTGCCGGAAATGCATTTCC<br>CATCGCCAAAAAGTCCTCTTGGATTCTCTGTTGTGACCACGCCTGCCGATGCAATCCGTGAGGATAGTATGGAACAGTAT<br>GTCATCGACGCCTGTGGTACGGAGAATTCATGTCTGACATTGACAGATGCCCCCATGGAAGCAAAGCGAATGCGGTCTCA<br>AAGCGCCTCTGTAGAAACGAAATTGAGCGAGGCATTTGATGCCATCGTCTGTGTTACAAGCGCCGGCAAGGACAGCCTGG<br>TTGCCCTATAGGAAACCGGAAATGAAAATGAGGGAGCATAACCTGCGAATCCACCGGACGGTTTTCAACCGCCGGTGATC<br>AGCGCG
ereA2	TTATGCTCTGTGAGCCTGGTTATTGGCGAAGCGAAAGTATGACGATTTCAGCAATAAACGCAAAAGGATAAAAAAATGAC<br>ATGGAGAACGACCAGAACACTTTTACAGCCTCAAAAGCTGGAGTTCAATGAGTTTGAGATTCTTAATCCCGTAGTTGAGG<br>GCGCCCGAATTGTCGGCATTGGCGAGGGTGCTCACTTTGTCGCGGAGTTCTCACTGGCTAGAGCTAGTCTTATTCGCTATT

	TTGTCGAGAGGCATGATTTTAATGCGATTGGTTTGGAATGTGGGGCGATTCAGGCATCCCGGCTATCTGAATGGCTCAACT<br>CAACAGCCGGTGCTCATGAACTTGAGCGATTTTCGGATACCCTGACCTTTTCTTTGTATGGCTCAGTGCTGATTTGGGTTA<br>AATCATATCTACGCGAATCAGGAAGAAAACTGCAGTTAGTCGGAATCGATTTACCCAACACCTTGAATCCAAGGACGAC<br>CTAGCGCAATTGGCCGAAATTATCCAGGTCATCGACCACCTCATGAAACCCCACGTTGATGCGCTGACTCAGTTGTTGAC<br>GTCCATTGATGGCCAGTCGGCGGTTATTTCATCGGCAAAATGGGGGGAGTTGGAAACGGCTCAGCAGGAGAAAGCTATCT<br>CAGGGGTAACCAGATTGAAGCTCCGTTTGGCGTCGCTTGCCCCTGTCCTGAAAAATCACGTCAACAGCGATTTTTTCCGAA<br>AAGCCTCTGATCGAATAGAGTCGATAGAGTATACGTTGGAAACCTTGCGTGTAATGAAAGCTTTCTTCGATGGTACCTCTC<br>TTGAGGGAGATACTTCCGTACGTGACTCGTATATGGCGGGCGTGGTGGATGGAATGGTTCGAGCGAATCCGGATGTAAGG<br>ATAATTCTGCTGGCGCACAACAATCATTTACAAAAAACTCCAGTTTCCTTTTCAGGCGAGCTTACGGCTGTTCCCATGGGA<br>CAGCATCTCGCAGAGAGGGAGGAGGGGGATTACCGTGCGATTGCATTCACCCATCTTGGACTCACCGTGCCGGAAATGCA<br>TTTCCCATCGCCCGACAGTCCTCTTGGATTCTCTGTTGTGACCACGCCTGCCGATGCAATCCGTGAGGATAGTGTGGAACA<br>GTATGTCATCGATGCCTGTGGTAAGGAGGATTCATGCCTGACATTGACAGATGACCCCATGGAAGCAAAGCGAATGCGGT<br>CCCAAAGCGCCTCTGTAGAAACGAATTTGAGCGAGGCATTTGATGCCATCGTCTGCGTTCCCAGCGCCGGCAAGGACAGC<br>CTGGTTGCCCTATAGGAAACCAGAAATGAAATGAAGGAGCATAACCTGCCAATCCACCGGACGGTTTTCAACCGCCGGTG<br>ATCAGCGCG
ereA3	TTAGAATCACTGAAAACTAAAAATAATTGCACAATCCGGCTCTACAGGTGGCAGAAGAAGTTATGACGGCATTGAATGGT<br>TGAAAAAATTAAGACAATTCAGGACAATGAAATTATTGGCGAAGCGAACGCATGACAGCAATGAGCGCAAAGGCTAAAA<br>AAATGACATGGAGAACTACCAGAACACTTTTACAGCCTCAAAAGCTGGACTTCAATGAGTTTGAGATTCTTACTCCCCTG<br>GTTGAGGGCGCCCGAATTGTCGGCCTTGGCGAGGGCGCTCACTTTGTCGCGGAGTTTTCACTGGCTAGAGCTAGTCTTATT<br>CGCTATTTGGTCGAGAGGCATGATTTTAATGCGATTGGTTTGGAATGTGGGGCGATTCAGGCATCCCGGCTATCTGAATAC<br>CTCAACTCAACAGCCGGTGCTCATGAACTTGAGCGATTTTCGGATCCACTGACCTTTTCTTTGTATGGCTCAGTGCTGATTT<br>GGATTAAATCATATCTACGCGAATCAGGAAGAAAACTGCAGTTAGTCGGAATCGATTTACCCAACACCTTGAATCCAAGG<br>GACGACCTGGCACAATTGGCCGAAATTATCAAGGTCATCGATCACCTCATTAAACCGCATGTTGATGAGCTGACTCACTT<br>GTTGGCATCCATTGATGGTCAGTCGGCGGTTATTTCATCGGCAAAATGGGGGGAGATGGAAACGGCTCAGCAGGAGAAA<br>GCTATCTCAGGGGTAACCAGATTGAAGCTACGTTTGGCATCTCTTGCCCCTGTCCTGAAAAAACATGTCAACAGCGATTTG<br>TTCCGAAAAGCCTCTGATCGAATAGAGTCGATAGAGTATACGTTGGAAACCTTGCGTATAATGAGAACTTTCTTCGATGG<br>TACCTCTCTTGAGGGAGATACTTCCGTACGTGACTCGTATATGGCGGGCGTAGTGGATAGAATGGTTCGAGCAAATCCGG<br>ATGTGAAGATAATTCTGCTGGCGCACAACAATCATTTACAAAAAACTCCAGTCTCCTTTTCGGGCGAGCTTACGGCTGTTC<br>CCATGGGGCAGCACCTCGCAGAGAGGGAGGAGGAGGATTACCGTGCGATTGCATTCACCCATCTTGGATCCACCGTGCCG<br>GAAATGCAATTCCCATCGCCCGGCAGTCCTCTTGGATTCTCTGTTGTGACCACGCCTGCCGATGCAATCCGTGAGGATAGT<br>ATGGAACAGTATATCATCGATGCCTGTGGTACGGAGGATTCATGTCTGACATTGACAGATGCCCCCATGAAAGCAAAGCG<br>AATGCGGTCCCAAAGCGCCTCTGTAGAAACGAATTTGAGCGAGGCATTTGATGCCATCGTCTGCGTCCCAAGCGCCGGCA<br>AGGACGGCCTGGTTGACCTATAGTAAACCGGTAGATGAAAATGATGGAGAATAACCTGCCAATCCACCGGACGGTTTTCA<br>ACCGCCGGTGATCAGCGCG
fosC2	TTATGTTCGCTGAGGAGAGTCAGTGTTACGAGGATTGAATCATATTACTATTGCAGTAAGTGACCTTGAACGTTCCGTGGA<br>GTTCTATACGCGTCTATTAGGAATGAAGGCACATGTCCGCTGGGATAGTGGGGCATATCTGAGCTTGGAGGCTACTTGGA<br>TTTGCTTGTCTTGTGACGAAGTGCATCCGAGCCAAGATTACTGTCACATCGCGTTTGATGTTTCCGAAGAGAATTTCGAAC<br>CAGTTACTAAAAAGCTTCGCGAAGCACATGTCGTTGAATGGAAACAAAATAGAAGCGAAGGACTTTCTTTATACTTGCTC<br>GATCCTGACGGCCATAAATTGGAAATCCATAGCGGTAGCCTACAAAGTCGTTTGGAATCGTTGAAGTCTAAACCCTATCA<br>AGGGTTAGTATGGCTATAAGCAAACATAACAAGTCAATTAACTACGCGCCTGCGGCGCCGGACGTGCTAGCGCACGCCG<br>GTTATTTCGGGCG
fosE	TTAGCGCCCATGGAAGGTATCAGCCACATCACGCTTATTGTCCGCGACCTCTCGCGCATGACCACCTTCCTTTGCGATGGT<br>CTCGGTGCGCGAGAGGTTTATGACAGTGCTGGCCACAATTACTCGCTTTCCCGCGAGAAATTCTTTGTCCTTGGTGGCGTT<br>TGGTTGGCCGCTATGGAGGGAGTGCCGCCATCTGAGCGCTCCTATCAGCATGTCGCCTTTCGGGTGAGTGAGTCAGATCTT<br>GCCGTATATCAGGCAAGACTTGGGTCGCTGGGCGTGGAGATTCGCCCACCCAGGCCACGCGTGAATGGAGAGGGGCTGT<br>CCCTGTACTTCTATGATTTTGACAACCATCTGTTTGAGCTGCACACCGGCACATTGGAGCAGCGCCTTGCCAGGTACGGAG<br>CTGGGCGCTAACAATTCATTCAAGCCGAAGCCGCTTCGCGGCTCGGCTTAATTCAGGCG
fosF	TTACGTTTTCTCAAGGTTCTCTGCATGATTACCGGCATCAATCACATCACATTTTCGGTTCGGGACCTGCGGGCATCGATT<br>GAGTTCTACCGTGATCTTCTGGGAATGAAGTTGCACGTATTCTGGGACACAGGTGCTTATCTCACTGCAGGCAATACGTGG<br>TTATGTTTGAGTTTGGGGCAGCCCGAACCCGCCAAGGACTACACACACGTCGCTTTCAGTGTCCGCGAAGGGGAGCTCCT<br>GGAGTTGCGAGCTAAACTAAAGCAGGCTGGCGTTGAAGAGTGGAAGCAGAATACCAGTGAGGGTGACTCCATCTATTTA<br>CTTGACCCAAATGGGCATCGCCTTGAACTGCATTGCGGAACACTGGCCACTCGCTTAGCTGAGCTGGAAAGCTCGCCTTA<br>TAAGGGGCTGGTGTGGAGCTGAAACGTAACAATCGGCTGTTGTCGCCCCTTCGGGGCTGGGACGGCCTTTTCGCCGCTGC<br>GCGGCTACAAGTCCGCCCCAAAGCCGGGCG
fosG	TTATGTTTGTTAAGGTAGATTTGTGCTCCGAGGATTGAACCACATCACCATCGCTGTAAGCGATTTAGGCCGTTCTCTCGC<br>CTTTTATACTGATATCGTCGGTATGCTCGCTCACGTACGCTGGGATAACGGTGCTTACCTTAGTCTAGGCGGTGTTTGGTTT<br>TGTCTTTCCTGTGACAAGGTGATGCCAAGTAAGGATTATTCTCATATTGCCTTAGATATTTCAGAAGATGACTTTGCATCA<br>TTTTTGGAGAAACTGAGGAGAGCCGATGTCACTGAGTGGAAACAAAATTCAAGTGAAGGCTATTCGGTGTATTTCTTAGA<br>TCCTGATGGAAATAAACTAGAAGCGCATAGCGGCTCGTTACAATCTCGTTTAAGTTCTTTAAAAGACAAACCTTATCCGG<br>GCTTAGTATGGCTTTAAACAAACATAACAAGCGCCATCAAAACGCCGCTTCGCGGCTCGACTCGCAACAAGTTGCTCGCG<br>TTTGTGGCGGGCG
fosH	TTAAAGCTCATCATGGGAATTCTTGGCATTAGTCATCTGACATTCGTGGTCCGCGATGTAGAGCGCACTGCGAGACTGGTC<br>TGCGAAGGACTTGGGGCGGAAGAGGTATACGACAGCAAAGCCAAAAACTTTTCGCTGTCACGAGAGAAGTTTTTTCTCTT<br>GGGCGGCGTATGGCTTGCTTTCATGGAAGGAGTGCCATCGGAGCGGTCCTATCGGCACGTCGCTTTTGAAGTGACCGAAG<br>AGGAAATTGCAAGATATGAGGCTAGCCTTAGAAACCTCGGTGTGGAAGTCAGAGAGCCGCGGCCAAGAGTGGCTGGAGA<br>GGGGCTGTCACTGTATTTCTACGACTATGACAACAACTTATTCGAGCTACATGCGGGAACACTGGCGCAACGACTTGAAA<br>GGTATACGCAATGAGTTCTAACAATCCGCTCCAGTGGACCGCCTGCGGCGTCCGCTGAGCTATCTCG
fosI	TTAGCTCCCATGAAAGGCATCAGCCACATCACATTCATCGTCCGCGACCTGAATCGTATGGCCGCACTTCTCTGTGAGGG<br>ACTGGGTGCGCGTGAGGTGTATGACAGCTCAAACCAGAACTTCTCGTTGTCCCGCGAAAAGTTCTTTGTGCTTGGTAGTAC<br>GTGGCTAGCTGCAATGGAAGGTGAACCGCCCGCCGAGCGTTCATATCAGCATGTTGCCTTTGCGGTGAGTGAGACGGACT<br>TGCCTGCGTATCAAGCCAGACTTGAGGCACTCGGCGTTGAGATTCGGCCACCGCGTAGCCGTGTTGACGGTGAGGGTCTC<br>TCCCTGTACTTCTACGACTTTGACAATCATTTATTTGAACTTCATTCAGGTACTTTAGAGCAGCGCCTTGTCCGGTATCAGG<br>CGGGGCGCTAACAATTCATTCAAGCTGACACCGCTTCGCAGCGCAGCTTAACTCAAGCG
fosK	TTACGTTTTAGGATCAGTCGTATGATCACTGGTATCAATCACATCACCTTTTCCGTCAGGGACTTGAGCTCTTCAATCGAG<br>TTCTATCGTGACTTGCTGGGAATGAGGCTGCACGTGACCTGGGAAGCAGGTGCTTATTTTACAGCGGGTGATACGTGGGT<br>ATGTCTGAGCGTCGGGGAACCTAAACCCGCCAACGACTACACGCATGTGGCATTCAGTGTTGGCGAAAGAGAGCTTGTTG<br>AGCTGCACGCTAGGCTAAAAGAAGCCGGGGTTGAGGAGTGGAAGCAGAATACAAGTGAGGGTAACTCCGTGTATCTGCT<br>TGATCCAAACGGCCATCGCATTGAGCTTCACTGCGGAACGTTGGCAACCCGCTTAGCTGAGTTGGAGAAGTCGCCCTATA<br>AAAGGTTGGTCTGGTGCTGAAACGTAACAATCGGCTGTTGCCGCCCCTTCGGGGCTGGGACGGCCTTTCCGCCGCTCCGC<br>GGCTACAAGTCCGCCCCAAAGCCGGGCG
fosL	TTAGCGCTCACTATGCAGATCGAGGGCATTAGTCACGTAACTTTCGTCGTAAAGGACCTAGAACGTGCATCCCGATTTTTC<br>TGTCAGGGTCTGGGAGCAACGGAGGTATACGACAGCAAGGGCAGCAACTTCTCTCTTTCACGAGAGAAGTTCTTTTTGGT<br>TGGTGGTGTCTGGGTTGCGGCAATGGAGGGCACACCACCCGCCACCGCCTCTTATCAGCACTTGGCGTTCAAGGTGGCGC<br>CCGAAGACTTGCCGCAGTTTGAGGCTCGTCTCCGCGCAATTGGTGTTGCCATCTCACCGCCGCGGGCGCGTGTCCAAGGC<br>GAGGGCTTGTCGCTGTACTTCCACGACTTCGACAATCACCTCTTCGAGCTCCACACTGGAACGCTTACTGAGCGCCTTCAA

	GCCTATGCAGCCCCGCGCTAACAAGTCCGTCAACGGGACGCCAAAATGCTGCGCATTTTGGTTCCCTCCGCTGCGCTCCG<br>GCGCCCGTTACGTCCAACG
fosM	TTAGGCCCACATAAACAAGCGCCCAAGAATATGCCAGTCAGTGGGGTTAGCCATATCACGTTCATGTCGCGCGACTTGGC<br>ACGCACAGCAAGAATATGGACCTATGGATTGGGTGCCACTGAGGTCTACGACTCGGGTGAGACGACGTTCTCACTGTCTC<br>AAGAGAAATTCTTCTTACTTGGGAGCACATGGATTGCGGTCATGCTTGGCGAGCCGGCACCAAGGTCGTATCACCACGTC<br>GCGTTTGAAGTAGACGACATTGACCTGCCCTCAATTGAGACGAAGCTAAAGGAGCTAGGTGTCGAGTTCATGCCCCCGCG<br>GGCCCGAGTCGATGGAGAAGGCAAATCGCTTTACTTCTATGACTTCGATGACAACCTGCTCGAGCTCCATGCAGGAACCC<br>TCCAACAGCGACTTGAGCGCTACAAGAAGGGGAAAAATGTCTAGTACGGAACGTCCTATCCTCATGTCGCAGCGCCTAAC<br>CGTTTGGTCAAGCCGACGCCCACAAGCCTCGCTTGTGGGTACCCTCCGTGCTACGCACTACGGCGCGGCTTACCGCGAGC<br>G
fosN	TTACGTTTTTTGAGGGACTGCATGATCACCGGAATAAACCACATTACTTTTTCTGTCAGTGATCTGGACTCATCGATCCAG<br>TTCTATCGTGGTTTCCTGGGCATGAAGCTTCACGTTCTTTGGGACACGGGTGCGTATCTGACAGCCGGTGATACATGGCTT<br>TGCTTGAGTTTGGGAGAGCCCGACCCCGCTAAAGACTATACGCATGTGGCCTTTAGTATCAGTGAAAACGCGCTCTCGGA<br>GCTACGCGCCAAACGGGGCGAGATGGGATTCAAGGAATGGAAGCAAAACACTAGTGAGGGCGAATCGCTGTACCTACTT<br>GATCCCAACGGCCATCGCCTAGAGCTTCACTGTGGCACTTTGGCAACTCGCTTGGCGGAGTTGGAGAACTCACCTTACAA<br>GGGGCTGGTATGGTGCTGAAACGTAACAATCGGCCGCACTGCGACCGCTTTTCCGCCGCTTCGCGCCTCCAAACCGGCGC<br>GTGAGCCGGGCG
foso	TTAGCGCTCACGATGCAGATCGAAGGCATTAGTCACGTTACTTTTGTCGTTAAGAGCTTGGCACGTGCTGCCGAATTCTTC<br>TGCCAAGGTCTCGGAGCAACTGAGGTCTACGACAGCGGGGGGCAGAACTTCTCGCTCTCTCGAGAGAAGTTCTTCTTAGT<br>AGGTGGTGTCTGGGTGGCGGCAATGGAGGGCATCCCACCTGCTGCCCGTTCCTATCAACACTTGGCGTTCAAAGTCGCGC<br>CCGAAGACTTGCCAAAGTTTGAGGCTCGTCTCCGCGCAATCGGCGTTGAGATTTCCCCGCCTCGACCGCGGGTCCAAGGA<br>GAGGGGTTGTCGCTGTACTTCCACGACTTCGACAACCACCTCTTCGAACTCCACACCGGAACGCTGACAGAGCGCCTGAA<br>CACCTATGCAGCCCCGCGCTAACAAGTCCGTCAACGGGACGCCAAACTGCTGCGCAGTTTGGTTCCCTCCGCTGCGCTCC<br>GGCGCCCGTTACGTCCAACG
lnu(F)1	TTGTGCGTAAGAATAAAACAAAAGCTGCGTATACCTTTCCGCAGAATCAGTGGCTTCAATAAAAGGATGTTTCAATGCTT<br>CAGCAGAAAATGATCGAACGCTTCAAGGAAGCTTGCCATGAGGATGCACGAATAATCGCGGCGCTGATGTTCGGCTCATT<br>TGCTATCGGAGAGGGTGACGAGTTCTCTGATATCGAATTTGCAGTGTTCATCCAGAATAATCATTTTGAAAATTTCGATCA<br>GCGCTCGTGGCTTAATGCTGTAAGTCCGGTTGCAGCTTACTTTCCGGATGACTTCGGCCACCACACCGCGCTTTTTGAAAA<br>CGGCATTCGCGGTGAATTCCATTTCATGCGAAAATCGGACATACCGGTCATTTCCACTTGGCAAGGCTACGGGTGGTTTCC<br>CTCGCTTGAGGAGGCTGTTTTGTTGGACCGATCAGGAGAGTTGTCAAGGTACGCGAGTGCTCTCGTGGGCAGTCCCCCGA<br>AACGTGAAGGCGCGCCGCTGGTGGAAGGACTTGTATTGAACCTCATCAGCCTGATGCTCTTTGGGGCAAATCTTTTAAAT<br>CGGGGAGAGTATGCTCGCGCCTGGGCTTTGCTCAGCAAAGCACATGAAAACTTACTCAAGTTGGTTCGCCTCCATGAAGG<br>GGCAACAGACCACTGGCCGACACCTTCACGCGCGCTCGAAAAGGATGTCTCGGAGGACTCGTATAATCGCTACCTGGCAT<br>GCACAGGCAGCGCGGAACCAAAAGCACTATGTGTAGCCTATCATGAAACGTGGAAGTGGAGTCTCGAATTGTTCAGGAG<br>TGTGGCTGGACCTCTGAATATCGAGCTTCCGAGAATTGTAATTGCGCAGACAAAAAGGTTGCTAAATGAATCTGCGACGC<br>CGCACAACAAGTAAATCCAGCGGACGCATAAAAGCGCGCCGCTGATTAACGCG
lnu(F)2	TTGTGCTGACGAAAAAAAACAAAAATCTGCGTATACCTTTCCTCATAATCTGTGGCTTCAATAAAAGGATATTTCTATGCT<br>TCAGCTGAAAATGATCGAACTCTTCAAGGAAGGTTGTCATGAGGATGCACGAATAATCGCGGCATTGATGTTCGGCTCAT<br>TTGCTATCGGAGAGGGTGACGAGTTCTCTGATATCGAATTCGCAGTGTTCATCCAGGATGACCATTTTGAAAATTTCGATC<br>AGCGCTCGTGGCTTAATGCCGTAAGTCCGGTTGCTGCTTACTTTCCGGACGACTTCGGCCACCACACCGCACTTTTTGAAA<br>ACGGCATTCGCGGTGAATTCCATTTCATGCGAAAATCGGACATACCGGTCATTTCCACTTGGCAAGGCTATGGGTGGTTTC<br>CCTCGCTTGAGGCGGCTGTTTTGTTGGACCGATCAGGAGAGTTGTCAAGGTACGCAAGCGCTCTCGTGGGCGGTCCCCCG<br>ATACGTGAAGGCGCGCCGCTGGTGGAAGGGCTTGTGTTGAACCTCATCAGCCTGATGCTCTTTGGGGCCAATCTTTTAAAT<br>CGGGGAGAGTACGCTCGCGCCTGGGCTTTGCTCAGCAAAGCACATGAAAACCTACTCAAGCTGGTTCGACTCCACGAAGG<br>GGCAACAGACCACTGGCCGACACCTTCACGCGCGCTCGAAAAGGATATCTCGGAGGACTCGTATAATCGCTATCTGGCAT<br>GCACAAGCAGTGCAGAACCAAGAGCACTATGTGCAGCCTATCATCAAACGTGGACGTGGAGTCTCGAATTGTTCAAGAG<br>CGTGACAGAACCTCTGAATATCGAGCTTCCGAGAACTGTAATTGCGCAGGCAAAAAGGTTGCTCAATGAGTCTGCGACGC<br>CGCACAACAAGTAAATCCAGCGGACGCATAAAAACGCGCCGCTGATTTTGACG
smr1	TTAGCCCCCACAATCGGAGATCGTTATGGGTTGGATATATCTCATTCTCGCTGGCGTCTTTGAAGTTGGTTGGCCAGTCGG<br>GCTCAAGATGGCGCAGACACCGGAGACTCGCTGGAGCGGCATCGGAGTGGCGGTTGCATTTATGACTGTGAGTGGGTTTT<br>TACTCTGGCTTGCGCAGCGGCAGATCCCCATTGGCACCGCATACGCGGTGTGGACAGGCATTGGTGCTGCCGGCACCTTT<br>TTCGTAGGCGTGCTGTACTACGGCGATCCGACTTCGTTCTTTCGGTACATGGGCGTCGCCCTGATAATAGCGGGCGTTATT<br>ACCCTCAAGTTGGCTCATTAGAGGTGTGCGCTAACAATTCATTCAAGCCCGACGCCCGCTTCGCGGCGCGGCTTAATTCA<br>GGCG
smr2	TTAGCGCCCACGAACGGAGATCACGATGGCCTGGATATACCTGATCCTCGCTGGACTCTTTGAGATTGGCTGGCCCGTCG<br>GACTGAAGATGGCGCAGGTACCGGAAACCCGATGGAGTGGTGTTGGCATTGCGGTCGCGTTTATGGCCGTGAGTGGCTTT<br>CTGCTATGGCTGGCTCAGCGGCACATTCCCATCGGTACTGCCTACGCTGTTTGGACGGGCATCGGTGCGGCTGGCACCTTC<br>CTCGTCGGCGTTCTTTACTACGGAGATCCAACATCAGTTGCGCGGTACTTTGGCGTGGCGCTCATTGTTGCCGGGGTCATC<br>ACGCTCAAGCTCGCGCACTGAGGTGTGGGCGCTAACAATTCATTCAAGCCGAAGCCGCTTCGCGGCGCGGCTTAATTCGG<br>GCG
smr3	TTAGACGCCCACGGAACGGGAGATCACGAGGGCCTGGATGGACCTGATCCTCGCTGGACTCTTTGAGATTGGCTGGCCCG<br>TCGGACTGAAGATGGCGCAGGTACCGGAAACCCGATGGAGTGGTGTTGGCATTGCGGTCGCGTTTATGGCCGTGAGTGGC<br>TTTCTGCTATGGCTGGCTCAAGCGGCAACATTCCCATCGGTACTGCCTAACGCTGTTTGGACGGGCATTCGGTGCGGCTGG<br>CACCTTCCTCGTCGGCGTTCTTTACTACGGAGATCCAACATCAGTTGCGCGGTACTTTGGCGTGGCGCTCATTGTTGCCGG<br>GGTCATCACGCTCAAGCTCGCGCACTGAGGTGTGGGCGCTAACAATTCATTCAAGCCGAAAGCCGCTTCGCGGCGCGGCT<br>TAATTCGGGCG
qacE	TTAGATGCACTAAGCACATAATTGCTCACAGCCAAACTATCAGGTCAAGTCTGCTTTTATTATTTTTAAGCGTGCATAATA<br>AGCCCTACACAAATTGGGAGATATATCATGAAAGGCTGGCTTTTTCTTGTTATCGCAATAGTTGGCGAAGTAATCGCAAC<br>ATCCGCATTAAAATCTAGCGAGGGCTTTACTAAGCTTGCCCCTTCCGCCGTTGTCATAATCGGTTATGGCATCGCATTTTA<br>TTTTCTTTCTCTGGTTCTGAAATCCATCCCTGTCGGTGTTGCTTATGCAGTCTGGTCGGGACTCGGCGTCGTCATAATTACA<br>GCCATTGCCTGGTTGCTTCATGGGCAAAAGCTTGATGCGTGGGGCTTTGTAGGTATGGGGCTCATAGTTAGTGGTGTAGTA<br>GTTTTAAACTTGCTTTCCAAAGCAAGTGCCCACTAATAAACTCAGTCATCTAACAAGTCGTTGCAGCACCGCTCCAGCACT<br>TCGTGCCTGCGCTGGACAGTTTTTAAGTCGCGGCTTTATGGTTTTGCTGCGCAAAAGTATTCCATAAAATCACAACTTAAA<br>AACTGCCGCTGAACTCGGCG
qacEΔ-sul1	TTAGATGCACTAAGCACATAATTGCTCACAGCCAAACTATCAGGTCAAGTCTGCTTTTATTATTTTTAAGCGTGCATAATA<br>AGCCCTACACAAATTGGGAGATATATCATGAAAGGCTGGCTTTTTCTTGTTATCGCAATAGTTGGCGAAGTAATCGCAAC<br>ATCCGCATTAAAATCTAGCGAGGGCTTTACTAAGCTTGCCCCTTCCGCCGTTGTCATAATCGGTTATGGCATCGCATTTTA<br>TTTTCTTTCTCTGGTTCTGAAATCCATCCCTGTCGGTGTTGCTTATGCAGTCTGGTCGGGACTCGGCGTCGTCATAATTACA<br>GCCATTGCCTGGTTGCTTCATGGGCAAAAGCTTGATGCGTGGGGCTTTGTAGGTATGGGGCTCATAATTGCTGCCTTTTTG<br>CTCGCCCGATCCCCATCGTGGAAGTCGCTGCGGAGGCCGACGCCATGGTGACGGTGTTCGGCATTCTGAATCTCACCGAG<br>GACTCCTTCTTCGATGAGAGCCGGCGGCTAGACCCCGCCGGCGCTGTCACCGCGGCGATCGAAATGCTGCGAGTCGGATC<br>AGACGTCGTGGATGTCGGACCGGCCGCCAGCCATCCGGACGCGAGGCCTGTATCGCCGGCCGATGAGATCAGACGTATT<br>GCGCCGCTCTTAGACGCCCTGTCCGATCAGATGCACCGTGTTTCAATCGACAGCTTCCAACCGGAAACCCAGCGCTATGC<br>GCTCAAGCGCGGCGTGGGCTACCTGAACGATATCCAAGGATTTCCTGACCCTGCGCTCTATCCCGATATTGCTGAGGCGG<br>ACTGCAGGCTGGTGGTTATGCACTCAGCGCAGCGGGATGGCATCGCCACCCGCACCGGTCACCTTCGACCCGAAGACGCG<br>CTCGACGAGATTGTGCGGTTCTTCGAGGCGCGGGTTTCCGCCTTGCGACGGAGCGGGGTCGCTGCCGACCGGCTCATCCT

	CGATCCGGGGATGGGATTTTTCTTGAGCCCCGCACCGGAAACATCGCTGCACGTGCTGTCGAACCTTCAAAAGCTGAAGT<br>CGGCGTTGGGGCTTCCGCTATTGGTCTCGGTGTCGCGGAAATCCTTCTTGGGCGCCACCGTTGGCCTTCCTGTAAAGGATC<br>TGGGTCCAGCGAGCCTTGCGGCGGAACTTCACGCGATCGGCAATGGCGCTGACTACGTCCGCACCCACGCGCCTGGAGAT<br>CTGCGAAGCGCAATCACCTTCTCGGAAACCCTCGCGAAATTTCGCAGTCGCGACGCCAGAGACCGAGGGTTAGATCATGC<br>CTAGCATTCACCTTCCGGCCGCCCGCTAGCGGACCCTGGTCAGGTTCCGCGAAGGTGGGCGCAGACATGCTGGGCTCGTC<br>AGGATCAAACTGCACTATGAGGCGGCGGTTCATACCGCGCCAGGGGAGCGAATGGACAGCGAGGAGCCTCCGAACGTTC<br>GGGTCGCCTGCTCGGGTGATATCGACGAGGTTGTGCGGCTGATGCACGACGCTGCGGCGTGGATGTCCGCCAAGGGAACG<br>CCCGCCTGGGACGTCGCGCGGATCGACCGGACATTCGCGGAGACCTTCGTCCTGAGATCCGAGCTCCTAGTCGCGAGTTG<br>CAGCGACGGCATCGTCGGCTGTTGCACCTTGTCGGCCGAGGATCCCGAGTTCTGGCCCGACGCCCTCAAGGGGGAGGCCG<br>CATATCTGCACAAGCTCGCGGTGCGACGGACACATGCGGGCCGGGGTGTCAGCTCCGCGCTGATCGAGGCTTGCCGCCAT<br>GCCGCGCGAACGCAGGGGTGCGCCAAGCTGCGGCTCGACTGCCACCCGAACCTGCGTGGCCTATACGAGCGGCTCGGAT<br>TCACCCACGTCGACACTTTCAATCCCGGCTGGGATCCAACCTTCATCGCAGAACGCCTAGAACTCGAAATCTAA
qacF	TTAGATGCCAGATTTGGCGTTGCGTATCTCACAGAAAATCGATAGCCGCAAGACTGTTTTTGGCAACTCATAGCCACTACA<br>ATTTCTCCTTCATACCGTAGAGGAGATTGCGCGTGAAGAACTGGATATTTCTGGCTGTTTCAATCTTTGGCGAGGTCATCG<br>CAACTTCCGCACTGAAGTCTAGCCATGGATTCACTAGGTTAGTTCCTTCCGTTGTAGTTGTGGCTGGCTACGGGCTTGCGT<br>TCTATTTCTTGTCTCTCGCGCTCAAGTCCATTCCGGTCGGTATTGCTTACGCTGTATGGGCTGGGCTTGGCATCGTGCTTGT<br>GGCAGCTATTGCTTGGATTTTCCATGGCCAAAAACTAGACTTCTGGGCGTTCATTGGCATGGGACTTATCGTCAGTGGCGT<br>CGCCGTTCTAAACCTGCTATCCAAGGTCAGCGCACATTGACCGGGTTGGCATCTAACAATTCATTCAAGCCGACGCCGCTT<br>CGCGGCGCGGCTTAATTCAGGCG
qacG	TTAGATGCTTTGCTGTGCGCACAAATTTCGGCCAGCAACAAGACTGTTTTTTTTCTTAAATCGAACCTAAAATTTCTTCGCG<br>GAACTCCATGGAGAAATATTTTGAAAAATTGGTTATTTCTGGCTACGGCCATTATTTCTGAGGTCATTGCAACCTCTGCGC<br>TCAAGTCTAGTGAGGGCTTTACTAGGTTAGTACCGTCTTTTATCGTCGTAGCGGGATACGCTGCTGCTTTTTATTTCCTGTC<br>GCTGACACTCAAATCGATTCCTGTTGCAATCGCCTACGCAGTTTGGTCGGGCCTCGGGATCGTCTTGGTCACTGCGATTGC<br>ATGGGTTTTGCATGGTCAAAAACTAGATATGTGGGGATTTGTTGGTGTCGGCTTCATTATCAGCGGCGTTGCTGTGCTCAA<br>CTTGCTATCTAAGGCAAGTGTTCACTAAAACGGTCGCATCTAACCATTCCGTCGAGAGGGACCGCCCACAAGCTGCGCTT<br>GCGGGTTCCCTTCGCGGCTTCGCCGCTACGGCGGCCCCTCACGTCAAACG
qacH	TTAGATGCCAGATTTGGCGTTGCGTATGCTCACAGAAAATCGACAGCCGCAAGACTGTTTTTGGCAACTCATAGCCACCA<br>CTATTTATCCTTCATACCGTAGAGGAGATTGCACGTGAAGAACTGGCTCTTTCTGGCTATTGCAATATTTGGTGAGGTCGT<br>CGCAACTTCCGCACTGAAGTCCAGCCATGGATTCACCAAGTTAGTTCCTTCTGTTGTAGTTGTGGCTGGCTACGGGCTTGC<br>GTTCTATTTCCTCTCTCTCGCAATCAAGTCCATCCCGGTCGGCATTGCTTATGCTGTTTGGGCTGGCCTCGGCATCGTACTT<br>GTGGCAGCTATCGCTTGGATCTTCCATGGCCAGAAACTAGACTTGTGGGCGTTCGTTGGCATGGGACTTATCGTTAGTGGC<br>GTCGCCGTTCTAAATCTGCTATCCAAGGTCAGCGCACATTGATCGGGCTGGCATCTAACAATTCATTCAAGCCGACGCCG<br>CTTCGCGGCGCGGCTTAATTCAGGTG
qacK	TTAGCATGTAATTAACCGGAAAGTTGCGCACGAATAATTTTTTCTTCAAGACTACAATTTTAAGAGAAAAAGACACATA<br>CTCCCTTCGAATCATATTGGAGGCGGATCATGAAAAGCTGGTTATTTCTTACTATTTCGATTCTTGGAGAGGTAGTTGCAA<br>CATCTGCATTAAAGTCCAGTGAAGGTTTTACGAAGCTCGTACCATCTTTCATCGTAATTGTTGGCTATGGAATTGCATTTT<br>ATTTCCTTTCATTGGTCTTGAAGTCTATTCCTGTAGGGGTGGCCTATGCAGTTTGGTCTGGCCTTGGCGTAGTATTAGTCAC<br>TGCTTTTGCATGGGGGCTTTACGGGCAAAAGATTGATGCATGGGGTTTCGTGGGTATTAGTTTAATAGTTTGCGGTGTATT<br>GGTTTTAAATTTGCTTTCTAAGGCAAGCGTTCATTAAGTGGCACTGGCATCTAACAATCGGCAGCAGGCGGACAGCCTAC<br>ACTACGCGGTTTTGTGTTACTCGCTGCGCTCAAACATTAACACAAAACCGCTCCGTTACGGCTGCCGCTGTGCCGGGCG
qacL	TTGGGCGTCTCAATACTGAGGATCACCGCAAATGCACTGGATACTTCTGACCGCAGCGATTATTAGTGAGGTCATCGGCA<br>CATCGGCCTTGAAGGCATCGGAAGGTTTCTCGCGTCTATGGCCTTCAGTTATTGTCACCATTGGCTACGCGCTGGCCTTCT<br>ACTTCCTCTCACTCACGCTCAAAACAATTCCAGTTGGGGTGGCCTACGCAATCTGGTCCGGAATTGGCATCGTTCTAATTG<br>CATTGGTTGCGTGGGTTCTCTATGGGCAAGCTCTTGATCTTCCGGCAATCATAGGCATGTCGCTCATCGTGGCTGGCGTTG<br>TTGTTCTCAATCTGTTTTCCAAGTCCGTCTCTCACTCATGAACCGCCCAACCCATCATTCCACCGGACCTGCGCAAAAAGC<br>CGCGCAGTCCGGTGAATTCAAACG
qacM	TTAGATGCCAGTGGTAGTTCCCGTAGGCGCACAGCTTTAGAGACAATACAGGCCTGCCGATTTTGAGATACAAGCCACAT<br>AATTGGCCGCCCATTATTTTGGAGTGAACTCATGAAAGCTTGGATCTATCTTGCCGTCGCGATAACCGCCGAAGTGGTCGC<br>AACTTCAGCGCTCAAATCGAGTGAAGGATTCACCAAACTTGCCCCGTCTGCCGTAGTTGTCATCAGCTACGGTGTAGCTTT<br>CTATTTTTGTCGCTCGTCTTGAAGACCGTTCCAGTCGGCGTTGCCTACGCTGTTTGGTCTGGCCTCGGCATCGTGCTGATC<br>GCAGCAGTTGCGTGGCTCTTTCATGGACAAAAGCTCGACACATGGGCCTTCGTTGGCATGGGACTGATAGTTAGCGGAGT<br>AGCGGTTCTCAACGTACTCTCCAAAACGAGCGCCCACTGATCCGGTGCTGGCATCTAACAGTTCATTCAAGCCGATACCG<br>CTTCGCGGCACGGCTTAACTCATTCG
qnrVC1	TTATGTGCTTTCTCTAAAACAAGCCAGATTCACTGAAACTTTCTTCTGGCTGGCCGCATCGGTCGTTTCGAATTAGCCACCT<br>TTAAAATGCCGTTTTGATGCCTTATTTTGGCTAAAACGGGGTGTTTTAAGTTTCTGTTTTTATTGGTTTTGTTTTTGTTGAGA<br>ACTTTCAGGTAAATGATAGTCTTCAAATCAAATGTTTTTTGGAGCCACAGCATGGAAAAATCAAAGCAATTATATAATCA<br>AGTGAACTTCTCACATCAGGACTTGCAAGAACATATCTTTAGCAATTGTACTTTTATACATTGTAATTTTAAGCGCTCAAA<br>CCTCCGAGATACACAGTTCATTAACTGTACTTTCATAGAGCAGGGGGCATTGGAAGGGTGCGATTTTTCTTATGCTGATCT<br>TCGAGATGCTTCATTTAAAAACTGTCAGCTTTCAATGTCCCATTTTAAGGGGGCAAATTGCTTTGGTATTGAACTGAGAGA<br>TTGTGATCTTAAAGGAGCAAATTTTAGTCAAGTTAGTTTTGTAAATCAGGTTTCGAATAAAATGTACTTTTGTTCTGCATA<br>CATAACAGGTTGTAACTTATCCTATGCCAATTTTGAGCAGCAGCTTATTGAAAAATGTGACCTGTTCGAAAATAGATGGAT<br>TGGTGCAAATCTTCGAGGCGCTTCATTTAAAGAATCAGATTTAAGCCGTGGTGTTTTTTCGGAAGACTGCTGGGAACAGTT<br>TAGAGTACAAGGCTGTGATTTAAGCCATTCAGAGCTTTATGGTTTAGATCCTCGAAAGATTGATCTTACGGGTGTAAAAAT<br>ATGCTCGTGGCAACAGGAACAGTTACTGGAGCAATTAGGGGTAATCATTGTTCCTGACTAAGCGCAAGATTCGGCTACGC<br>ACATAACAAACGCTTTAAGACGGATTCGCAACGTTTGGCGGTTTTAGTTTGAATTGGCTTTTGTATTTACGGTGTAATAAT<br>TGAGTGTTGTGGTAGCGTTGCTCACCACTTAAGCGGGCG
qnrVC6	TTATGTGCTTTCTCTAAAACTCACCAGGTTCACCAAAACTTTTTTCTAGCTGGCCGCATCGCTTATTTTGAATTAGCACCCT<br>TTAAAATGCCATTTTGATGCCTTATCTTGGCTAAAACGGGGTGTTTTAAGTGTCTGTTTTTATTGGTTTTGTTTTTGTTGACA<br>ACTTTCAGGTAAATGGTAGTCTTCAAATCAAATGTTTTTTGGAGCTACAGCATGGAAAAATCAAAGCAATTATATAATCA<br>AGTGAACTTCTCACATCAGGACTTGCAAGAACATATCTTTAGCAATTGTACTTTTATACATTGTAATTTTAAGCGCTCAAA<br>CCTTCGAGATACACAGTTCATTAACTGTACTTTCATAGAGCAGGGGGCACTGGAAGGGTGCGATTTTTCTTATGCTGATCT<br>TCGAGATGCTTCATTTAAAGATTGTCAGCTTTCAATGTCCCATTTTAAGGGGGCAAATTGCTTTGGTATTGAACTGAGAGA<br>TTGTGATCTTAAAGGGGCAAATTTTAGCCAAGTTAGTTTTGTAAATCAGGTTTCGAATAAAATGTACTTTTGCTCTGCATA<br>CATAACAGGTTGTAACTTATCCTATGCCAATTTTGAGCAGCAGCTTATTGAAAAATGTGACCTGTTCGAAAATAGATGGAT<br>TGGTGCAAATCTTCGAGGCGCTTCATTTAAAGAATCAGATTTAAGTCGTGGCGTTTTTTCAGAAGACTGCTGGGAACAGTT<br>TAGAGTACAAGGCTGTGATTTAAGTCATTCAGAGCTTTATGGTTTAGATCCTCGAAAGATTGATCTTACAGGTGTAAAAAT<br>ATGCTCGTGGCAACAGGAGCAGTTACTGGAGCAATTAGGGGTAATCATTGTTCCTGACTAAGCGCAAGATTCGGCTACGC<br>ACATAACAAAGCGTTTAAGACAGATTCGCAACGCTGGGCATTTTCGGTTTGCTTTGAATTTAGTGTTTACGGCACAATGGT<br>TTAGGTTGGGTGGCATGTTGCTCACTACTTAACGCGGCG
sul4	TTAGCTTGACCCTTCGGGCGGAGGAGAAAATGTCATCAATTTATAATAAAACTAAAAGTGGATTTTGTCGGATTTGTGGA<br>AATTGTAAGAAAGACAGCGTTTTTCTTCCAGTTTTGAAATTGGGCGTAAACCCACTTTTAAATGGGGGCCGCGCCTCATCG<br>ACATCGACATCCTTTTCTATGACAACATGATCATGAACGACGATATCCTGGCTATTCCTCATCCCTTCGTCGATGAACGTG<br>CTTTTGTCCTGGCACCCCTGGCTGACATTGCGCCCGATTACGAACATCCCCAGACCCAAAAGTCTGTAGCCCAAATGCTGG<br>CTGACGTATCCTCATCCGTAGATGAGGCCACAACGGCCGTTTACCGTTTACCGGATCAACCAAACTTCATGGGCACTTAA<br>CCCCCACCCTAACCCTCCCCCTAAAATGGGGAGGGAACATCTCCCTCTCCCGGTGGGAGAGGGCTGGGGAGAGGGTGAA<br>AAAAATTTTCAAGGAACTGCTATTGATGTCAACCACACTAACCAGCTTCAAATGGGGTGAACGCACCTACATCATGGGCA

TCCTCAACGTCACTCCAGACAGCTTTTCTGGAGATGGCGTTATGGTTGAAGAAGATGTCATCGCCAAAGCGGTAGCCCAG
GCCAAACAATTTGTAGCCGACGGCGCAGACATCATCGACATTGGCGGCGAGAGTACCCGCCCTGGCAGCTCACCTATAAG
CGCAGAGGAAGAACTGGCGCGGGTGCTGCCGGTGGTGCAGGCCGTACGCCAGGCTGTGGACGTCGTTATTTCCATCGACA
GCTACCGCGCTTCCGTGGCCGAAGCGGCCCTGGCGGCAGGCGCCAGCTGGCTCAACGACGTCTGGGGGCTGCGCATGGA
CCCGGACATGGCCGGCCTGGCAGCACAAGCCGGCTGCCCCATCGTCCTTATGCACAACCGCAGCAAACCAAAGAACATA
GCGCAAGAAAAAAAGCTGGGCGGGCGCTTCATCGGGGTAAAATACGACGACCTCATCACCGACGTTAAACGTGAATTAC
AAGAAAGCATCGACATCGCCTTAAAAGCCGGCGTAAAAGAGTCCCAAATTATCCTGGATCCCGGCATCGGCTTCGGTAAA
ACCGTCGAGCAAAGTTTGCAACTGCTCGACCAGATTAATCAGTTCAAAACAATGGGATTTCCCATCTTAATAGGTCCGTC
GCGCAAATCATTTATTGGCTATACGCTCGATTTGCCGCCAGACCAGCGCATAGAAGGAACGGCGGCCACCGTCGCCATTG
GCATTGACCGAGGAGCCGACGTTGTGCGCGTCCATGACGTCAAAGCAATCGTTCGGGTCGCCCGTATGACAGATGCAATC
GTGAGACGTTAAAGGATTTCAGTGCTATCAGTCACTAGTGAAATGCGTAAAACGTAATGCGTAAGGTTTATTACGCATTA
CGCATTACGAATCAAACCACAAACACACTGAATTTAGACACATTTTATAGGAGCTGCACATGATCGATTTTGAACTAGAC
CAGGAACAAAAGATGCTAACCGATGCCATCCGCCGCCTGGCGGAAGAGCGCATACGGAATCTGTAGCAACAAGGCAGGT
GGTTCCGTTCTCAGTGCCACCTTCAAGTCAGGGAGTGAACATGAGGTTCGGCAGCTTCGTTCTCAGTGGCATCAACAAGTC
CAGCTTGGTCATCAGCTTTGGCGCCCTGTCATGCTTCGTTTTGCCTCCGGCTTCGCTTTCGCTCCGCAAATTTCGCTGCTTC
AAGCTCAGTGTGCTGGCCCATAACAAGGGCAATCAGTTCGCGCCTGGCGGCGCCGGACGCTCCCTGCGGTCGCGCCGCTG
TTGCCGTCG

Supplementary Table 3. Antibiotic discs used in this study.

Reference	Antibiotic (Ab.)	Ab. content (μg)
CT0026B	Kanamycin (K)	30
CT1897B	Streptomycin (S)	300
CT0024B	Gentamicin (CN)	10
CT0056B	Tobramycin (TOB)	10
CT0107B	Amikacin (AK)	30
CT0545B	Apramycin (APR)	15
CT0033B	Neomycin (N)	30
CT0003B	Ampicillin (AMP)	10
CT0623B	Ciprofloxacin (CIP)	1
68042	Amoxycillin (AMO)	20
66178	Amoxycillin Clavulanic Acid (AMC)	20/10
CT0149B	Cefaclor (CEC)	30
CT0166B	Cefotaxime (CTX)	30
CT0119B	Cefoxitin (FOX)	30
CT0412B	Ceftazidime (CAZ)	30
CT0455B	Imipenem (IPM)	10
CT0774B	Meropenem (MEM)	10
CT1761B	Ertapenem (ETP)	10
CT0264B	Aztreonam (ATM)	30

Supplementary Table 4. Oligos used in this study.

Oligo	Sequence 5´→ 3´	Description
Int R bb	CTTTGTTTTAGGGCGACTGC	plasmid linearisation
GFP F bb	TTAGGCGTCGACGCTGCA	plasmid linearisation
gBlock F	GCAGTCGCCCTAAAACAAAG	ARC amplification
gBlock R	GCTGCAGCGTCGACGCCTAA	ARC amplification
Int F	AACGCAATTACAGAAATG CCTCGACTTCGC	Sequencing
GFP R	AAAGCGCTGTCTAGACTATT	Sequencing
GFP 2.0 R	CTCGATTCTATTAACAAGGG	Sequencing

Supplementary Table 5. Antimicrobial compounds used in this study.

Reference	Trader	Antimicrobial
T7883-5G	Sigma Aldrich	Trimethoprim
K4000-5G	Sigma Aldrich	Kanamycin sulfate
S6501-25G	Sigma Aldrich	Streptomycin sulfate salt
G1914-5G	Sigma Aldrich	Gentamicin sulfate
PHR1079-1G	Sigma Aldrich	Tobramycin
A1774-1G	Sigma Aldrich	Amikacin
A2024-1G	Sigma Aldrich	Apramycin sulfate salt
A8523-5G	Sigma Aldrich	Amoxicillin
C6895-1G	Sigma Aldrich	Cefaclor
CDS020667-50MG	Sigma Aldrich	Ceftazidime
901967-H	MSD	INVANZ (Ertapenem)
PZ0038-25MG	Sigma Aldrich	Aztreonam
P5396-1G	Sigma Aldrich	Phosphomycin disodium salt
E5389-1G	Sigma Aldrich	Erythromycin
R3501-1G	Sigma Aldrich	Rifampicin
C0378-25G	Sigma Aldrich	Chloramphenicol
B6295-100G	Sigma Aldrich	Benzalkonium chloride
282227-1G	Sigma Aldrich	Chlorhexidine
H6269-100G	Sigma Aldrich	CTAB (Hexadecyltrimethylammonium bromide)
S7507-10G	Sigma Aldrich	Sulfamethoxazole

Supplementary Figure 1. Resistance characterisation of *aa* ARCs by agar diffusion test. Antimicrobial resistance to several aminoglycosides is shown as a reduction in growth inhibition halo (mm) in comparison with the parental strain in the presence of these antibiotic discs. Dark red correlates with higher resistance (smaller inhibition halo), while lighter red denotes lower resistance. Blank cells represent a variation in inhibition halo ≤5 mm (as observed for control antibiotic discs of ampicillin and ciprofloxacin). A phylogenetic tree showing sequence homology between the encoded proteins is shown in the left side of the graph.

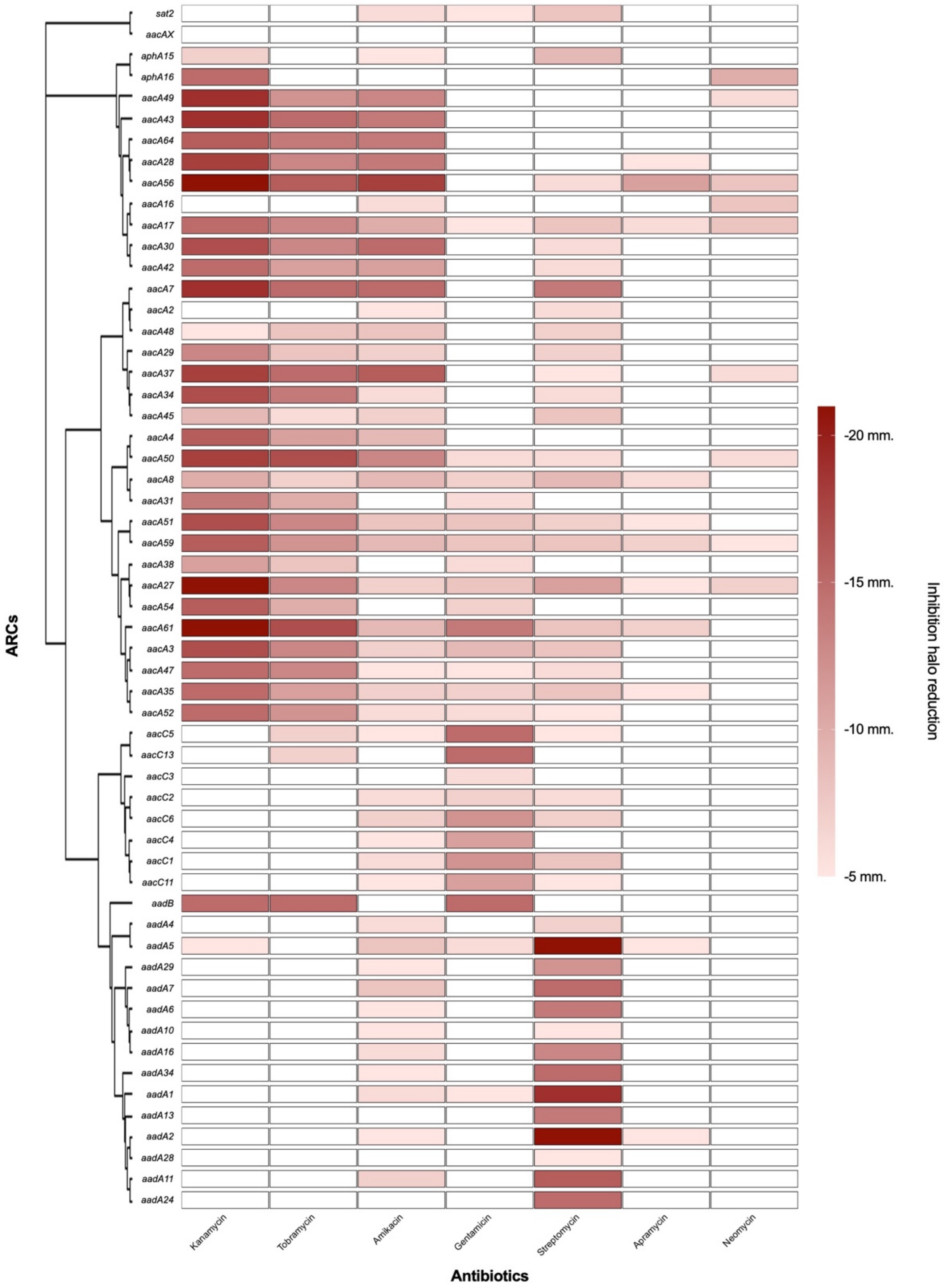

Supplementary Figure 2. Resistance characterisation of *bla* ARCs by agar diffusion test. Antimicrobial resistance to several beta-lactams is shown as reduction in growth inhibition halo (mm) in comparison with the parental strain in the presence of these antibiotic discs. Dark blue correlates with higher resistance (inhibition halo decrease), while light blue denotes lower resistance. Blank cells represent a variation in inhibition halo ≤4 mm (as observed for control antibiotic discs of kanamycin and ciprofloxacin). A phylogenetic tree showing sequence homology between the encoded proteins is shown in the left side of the graph.

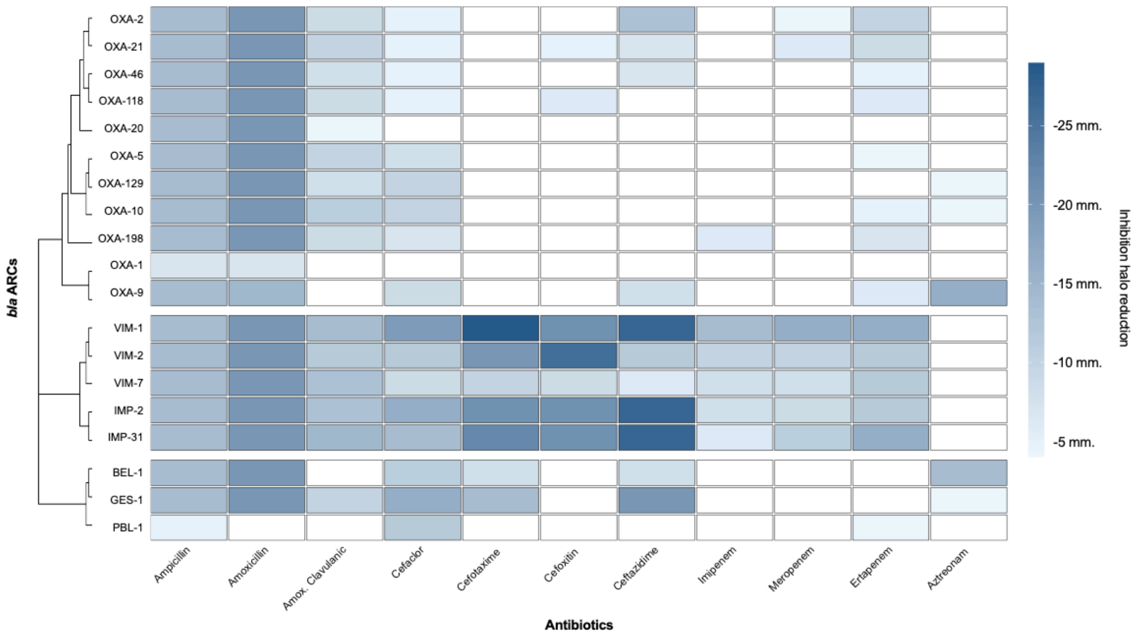

Supplementary Figure 3. Schematic representation of the cloning region in pMBA derivates. Each ARC was cloned in its native environment in the first position of the array (integron attachment site or *attI* site) and followed by a GFP gene. The primers used both for backbone and ARCs amplifications are represented in orange.

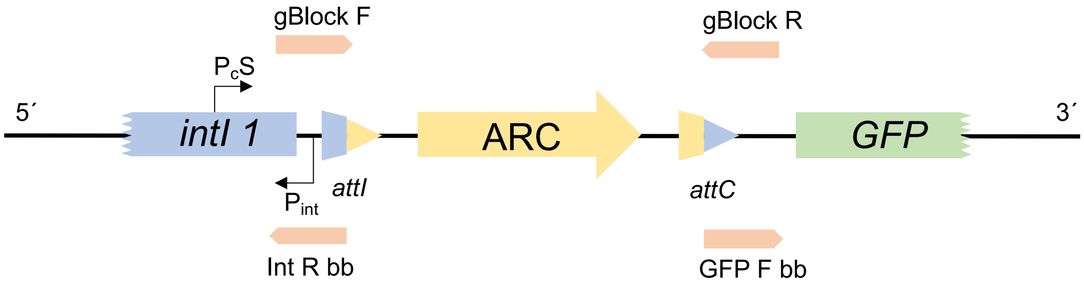

Supplement: Supplementary file 1 — Supplementary Material [file 44259_2023_14_MOESM1_ESM.pdf]
